# Wavelength-Specific UV-C Inactivation of Viruses in Liquids: Dose–Response, Mechanistic Insights, and Structural Integrity—A Systematic Review and Meta-Analysis

**DOI:** 10.3390/v18030276

**Published:** 2026-02-24

**Authors:** Roland Hetényi, Dániel Hanna, Zoltán Kopasz, József L. Szentpéteri, Péter Szabó, Balázs Antal Somogyi, Krisztián Bányai, Edina Szabó-Meleg

**Affiliations:** 1 National Laboratory of Virology, University of Pécs, 7624 Pécs, Hungary; roland.hetenyi@aok.pte.hu (R.H.); daniel.hanna@aok.pte.hu (D.H.); kopasz.zoltan@pte.hu (Z.K.); szabo.peter3@pte.hu (P.S.); somogyi.balazs@pte.hu (B.A.S.); 2 Virological Research Group, Szentágothai Research Centre, University of Pécs, 7624 Pécs, Hungary; 3 RoLink Biotechnology Ltd., 7624 Pécs, Hungary; 4 Institute of Transdisciplinary Discoveries, Medical School, University of Pécs, 7624 Pécs, Hungary; szentpeteri.jozsef@pte.hu; 5 Department of Medical Biology, Medical School, University of Pécs, 7624 Pécs, Hungary; 6 Department of Pharmacology and Toxicology, University of Veterinary Medicine, 1078 Budapest, Hungary; 7 Department of Biophysics, Medical School, University of Pécs, 7624 Pécs, Hungary; edina.meleg@aok.pte.hu

**Keywords:** wavelength, inactivation, biosafety, disinfection, virus, integrity, biomolecules, pathogens

## Abstract

This study evaluates fragmented data on ultraviolet-C (UV-C, 100–280 nm) irradiation for viral inactivation in liquid media, supporting advances such as whole-pathogen vaccine development and downstream research. Included studies reported viral strain identification, baseline titers (PFU or TCID_50_), UV-C wavelength, dosage, and log reductions, excluding studies employing alternative treatments. We searched (PubMed, Ovid Medline, Scopus, Embase, Web of Science; 10 April 2024) and identified 2813 records, of which 33 met the inclusion criteria. Risk of bias was assessed using ROBINS-I V2 to evaluate methodological rigor and inform improved reporting. Narrative synthesis summarized findings across viruses, while meta-analysis focused on 16 SARS-CoV-2 studies with standardized reporting. Meta-regression revealed a strong dose–response relationship (log_dose β = 3.38, 95% CI [2.95, 3.82], *p* < 0.001) with low heterogeneity (I^2^ = 15.1%). Strain and wavelength-specific efficacy peaked at 267 nm (β = 6.42) and 275 nm (β = 3.78), while 253.7 nm offered structural preservation for downstream applications. Limitations included inconsistent dose reporting, matrix effects, and assay sensitivity. We propose a refined reporting framework and standard definitions for ‘inactivation’, ‘‘disinfection,’ and ‘complete inactivation.’ Our findings support reproducible UV-C evaluation, regulatory alignment, and safe implementation in pathogen control, biosafety, and clinical applications.

## 1. Introduction

Understanding the effects of ultraviolet radiation ranging from 100 to 280 nm (UV-C) on viral inactivation is critical not only for biosafety in microbiological and biomedical laboratories but also for infection prevention in clinical environments such as operating rooms, ICUs, and dialysis units [1,2,3,4,5,6]. Furthermore, such chemical-free pathogen inactivation may protect animals and the environment, reinforcing their interconnection within the One Health framework, and help prevent outbreaks [7,8,9,10]. Although UV-C has long been widely applied even before chemical inactivation, evidence for its effectiveness is often incomplete or inconsistent and meets skepticism [11,12,13]. As an example, organism-specific variability complicates UV-C effectiveness, as claims of ‘99.9% or 99.99%’ pathogen inactivation often assume fixed doses and overlook key factors such as viral structure, genome type, initial load, and repair or resistance mechanisms. A starting concentration of 10^7^, common for certain viruses, would demand 99.99999% reduction for complete inactivation. In parallel, broader concerns about scientific credibility persist, for example, recent replication efforts in Brazilian biomedical research found that only 21% of experiments were reproducible, with original studies often reporting inflated effect sizes and reduced variability [14]. Such claims risk being misleading and underscore the need for stronger scientific and regulatory caution at a time when public skepticism toward science continues to rise [15,16,17]. These issues reflect limited attention to the type and quality of evidence required for reliable inactivation and preserved biomolecules, as only a few studies have achieved intact viral proteins, with results that remain difficult to replicate. In light of all this, critically evaluating UV inactivation is essential; it remains the collective responsibility of the virology and microbiology community to ensure proper assessment, implementation, and use through an open review of its strengths and especially its limitations. Undoubtedly, if UV-C is consistently applied for such purposes, it could yield substantial benefits by preserving essential biomolecules, thereby supporting research applications.

No study has clearly mapped the trade-off between viral inactivation and biomolecule preservation across UV-C wavelengths in liquids. However, challenges in science often present opportunities for advancement and discovery. During preliminary research, we noted that a variety of experimental setups with differing depths of data reporting aimed to preserve viral structures. Interestingly, UV-C at 253.7 nm can inactivate viruses while preserving nucleic acids and proteins, making it a promising and enabling approach that biotechnology and healthcare could strategically leverage [18,19,20,21]. Thus, our study investigated viral inactivation in liquids relevant to clinical applications to inform molecular, pathogenic, and epidemiological strategies for controlling, researching, and inactivating viruses of medical importance.

We aimed to understand how UV-C inactivation can be applied to preserve the integrity of viral biomolecules. In other words, we hypothesize that the spectral efficacy of UV-C irradiation in liquid media is wavelength- and dose-dependent, such that intermediate wavelengths enable viral inactivation while preserving proteins and viral structures, whereas higher-efficacy wavelengths achieve stronger inactivation at the expense of targetable structural preservation. By addressing the aforementioned unresolved questions, we seek to elucidate the underlying mechanisms of UV-C irradiation [22,23,24,25]. With the high-quality but fragmented literature in mind, we explore data reporting, dose–response relationships, wavelength-specific mechanisms, and semantic distinctions between ‘inactivation’ and ‘disinfection’ and propose a consistent definition to establish a rigorous, standardized criterion for virology and biosafety. In line with recent editorials calling for scientifically sound and policy-relevant research, we propose operational definitions to support regulatory harmonization and the development of inactivated viruses for subsequent use in diagnostic, therapeutic, and other healthcare applications [26,27,28,29,30,31]. Our meta-analysis consolidates fragmented evidence into the first wavelength- and dose-resolved roadmap showing where viral inactivation and biomolecular integrity may overlap, while providing a standardized reporting framework and quantitative parameters needed to guide and validate future structure-preserving UV-C experiments.

## 2. Materials and Methods

### 2.1. Protocol and Registration Details

This study adhered to PRISMA, MECIR, and GRADE guidelines to ensure transparent and rigorous reporting [32,33,34]. Our systematic review and meta-analysis were registered with PROSPERO, last edited on 22 August 2024. Available at Registration ID: CRD42024537243 or https://www.crd.york.ac.uk/PROSPERO/view/CRD42024537243 (accessed on 20 September 2025). The review protocol can be accessed in the PROPSPERO registration. Modifications prior to data extraction occurred, where aerosolized and surface procedures were excluded because of their potential impact on the sample being treated, which occurred during the date of reviewer training.

### 2.2. Eligibility Criteria

This systematic review identifies gaps in UV-C-based viral inactivation, while the meta-analysis applies strict criteria to achieve reliable statistical synthesis within a defined virus strain–dose–wavelength framework for SARS-CoV-2, where data quality and quantity allowed meaningful analysis. Inclusion criteria required studies to specify viral strains (1), report initial Tissue Culture Infectious Dose 50% (TCID50) or Plaque-Forming Unit (PFU) concentrations (2), data on UV-C devices (100–280 nm) (3), applied dosages (4), and viral load reductions (5). We only included observational studies reporting viral inactivation using UV-C in liquid media (6). Exclusion criteria ruled out studies lacking viral propagation data (1), using dry surfaces or alternative inactivation methods (2), combining multiple UV wavelengths (3), or omitting key operational details such as operational time of UV-C device (4). Randomized controlled trials (RCTs) were not part of our inclusion criteria, as our primary aim was to evaluate the technical efficacy of UV-C radiation in in vitro settings, not clinical effectiveness.

### 2.3. Search Strategy and Information Sources

To ensure transparency and reproducibility, the literature search followed the PICO framework (Population, Intervention, Comparison, Outcomes). This framework allows us to define and align the research question with both relevant MeSH terms and keywords, ensuring accuracy and comprehensive retrieval of studies across databases; see Table 1 [35].

We jointly searched Pubmed, Ovid Medline, and Journals@OVID full text, Scopus, Embase, and Web of Science on 10 April 2024, at 10:00, UTC 1, retrieving 2813 total results. We included only English-language studies from the past five years to capture recent, high-quality biomedical research reflecting current UV-C technologies, protocols, and advances in virology and inactivation methods. Grey literature sources such as books, dissertations, and conference proceedings were excluded to enhance reliability and validity. For the extensive search strategy, see Supporting Information 01.

### 2.4. Selection and Data Collection Process—Data Items

After duplicate removal, reviewers and a senior author held a training session (7 August 2024, 1:55 pm UTC 2) to resolve discrepancies and apply inclusion–exclusion criteria by simulating reviews on 10 virus-related articles. Full-text screening criteria were finalized during data collection here to ensure consistency. The second screening used abstracts resembling our prior work, with Power BI desktop (2.150.2455.0) and the PubMed API retrieving missing entries via PubMed IDs [36]. Both automatically and manually retrieved abstracts were verified by at least two reviewers. With the screening of titles, we had 618 articles remaining, and consequently, the screening of abstracts resulted in 308 articles, which were sought and screened in the full-text form gradually, first checking for reported TCID50 or PFU-s, appropriate UV-C and Viruses, surfaces, and solutions, and later for study types. After developing the workflow in training, two reviewers independently screened articles using a shared Excel database (Microsoft^®^ Excel^®^ for Microsoft 365 MSO, Version 2503 Build 16.0.18623.20116, 64-bit) linked to Microsoft Forms, allowing real-time tracking of reviewer actions; see Table 2 and Supporting Information 02.

All data conversions were cross-checked; see Supporting Information 02. Viral strains were identified using GenBank or GISAID IDs, with sample handling, storage, and suspension media assessed; 25 °C was assumed if temperature data were missing [37,38,39].

To improve data completeness, authors were contacted via email and ResearchGate, with a 20% response rate, resulting in one eligible study. Inconsistencies in virus strain labels and UV wavelengths (e.g., 253.7 nm vs. 254 nm) were standardized. Most often, the low-pressure mercury (LPM) lamp emits a narrow spectral line centered at 253.7 nm, with a reported full width at half-maximum (FWHM) of approximately ≤ 2.64 nm, consistent with values [40]. Extreme values (e.g., doses far above complete inactivation thresholds) were excluded to avoid sigmoid curve distortion. No imputation or further filtering was applied.

### 2.5. Study Risk of Bias Assessment

Given the heterogeneity in UV-C devices (LEDs, low- and high-pressure lamps), exposure distances, solution compositions, and irradiance conditions, we applied ROBINS-I V2 to strengthen analytical reliability. Two reviewers conducted joint screening, with a third resolving discrepancies [32,33,34,41,42]. We built custom Excel visuals based on Cochrane’s tool to show risk of bias [43].

### 2.6. Data Analysis

#### 2.6.1. Effect Measures

UV doses were log-transformed [32,33,34]; strain and wavelength were categorized for structured analysis. For each study, we recorded: (a) UV dose (mJ/cm^2^), log_10_ reduction, wavelength (nm), and strain metadata; (b) effect estimates as mean differences in log_10_ reduction per unit log_dose with 95% CIs from meta-regression and ANOVA, plus BCa bootstrapped intervals when applicable. log_reduction was the primary outcome, modeled as change per unit log_dose using regression coefficients (β). Virus strain and wavelength were added as categorical moderators with mean differences and CIs as effect measures. We extracted raw data or computed missing values such as survival fractions, dose, and log reductions from available information (e.g., PFU or TCID_50_ values). These were harmonized to create a consistent statistical framework for dose–response modeling; see Supporting Information 03 and 04. Outcomes were defined as log_10_ reductions in viral titers measured by TCID_50_ or PFU. Eligible exposures included specific UV wavelengths and dosages, while outcomes were quantifiable viral load reductions. For the statistical exploration and meta-analysis, we utilized IBM SPSS Statistics (v29.0.2.0) [44,45,46]. Sixteen SARS-CoV-2 studies met consistency criteria by reporting UV-C parameters and viral load reductions, making them suitable for meta-analysis and enabling robust dose–response modeling across independent, comparable experiments. We used Power BI for cross-thematic and qualitative word occurrence analysis; see Supporting Information 05. Figures were exported from SPSS and converted to Excel for easier editing. Illustrative figures were created using Microsoft PowerPoint (Microsoft^®^ PowerPoint^®^ for Microsoft 365, Version 2601, Build 16.0.18628.20166).

#### 2.6.2. Synthesis Methods

We applied random-effects meta-regression using REML with a truncated Knapp-Hartung adjustment to account for methodological heterogeneity due to differences in virus strains, UV wavelengths, and dose–response patterns. Moderators included virus strain, UV wavelength (categorical), and log_dose (continuous), with interaction terms (e.g., UV wavelength × log_dose) to explore effect modification. Subgroup differences were tested using univariate ANOVAs and contrast analyses (Helmert, polynomial). We conducted subgroup analyses based on UV wavelength and virus strain. Differences in inactivation were assessed using ANOVA and interaction terms in the meta-regression model. Virus strain and wavelength were treated as categorical moderators to assess differential susceptibility and efficacy. Heterogeneity was evaluated with τ^2^, I^2^, and the Q-statistic, and residual variance was reassessed post-modeling. Reliability was strengthened using robust HC3 standard errors and BCa bootstrap resampling (1000 samples). Even after filtering, issues like heteroscedasticity and non-linearity persisted, highlighting design and reporting limitations.

To test robustness, we re-estimated models excluding low-quality or incomplete records, and extreme outliers from dose–response tails (e.g., full inactivation points), confirming consistent effects, particularly for log_dose and UV wavelengths of 267/275 nm. Pairwise comparisons and residual plots supported a good model fit. The final model demonstrated a strong overall effect, primarily driven by the log_dose predictor, with R^2^ = 95.2% and I^2^ = 15.1%. However, the high F-statistic (F = 17.881, *p* < 0.001) should be interpreted cautiously, as most categorical moderators were not statistically significant and may reflect overfitting due to sparse data across virus strain levels. Given our analysis aimed at explaining heterogeneity, variations in liquid media (Phosphate-buffered saline, Dulbecco’s Modified Eagle Medium, Eagle’s Minimum Essential Medium) likely contributed to between-study differences; however, after filtering, residual heterogeneity was reduced to I^2^ = 15.1%. This level is classified as low (0–25%), indicating that results were broadly consistent and that dose–response relationships remained robust despite unavoidable media-specific confounding. Because all included studies focused on SARS-CoV-2, the medium type may be considered technical noise. Further stratification by medium would have risked overfitting the limited dataset. Thus, treating media differences as residual variability was justified.

#### 2.6.3. Study Risk of Bias Assessment and Certainty Assessment

**Figure 1:**
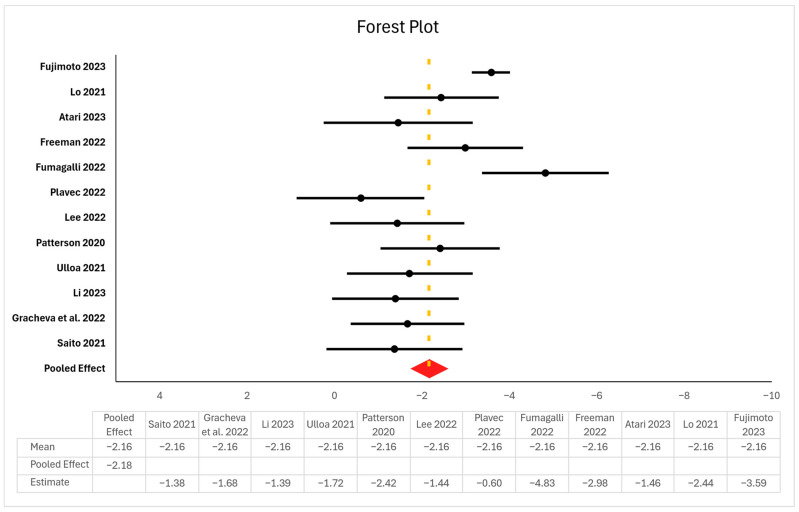
Trimmed Forest plot of 14/16 cleaned studies from a random-effects meta-analysis. Black dots show effect sizes with 95% CIs; the dashed yellow line represents the mean estimated overall effect size. The red diamond indicates a significant pooled effect of −2.18 [CI: −2.61, −1.74]. Moderate heterogeneity (I^2^ = 55.2%, *p* = 0.01). Each point represents the mean effect size of an individual study (Fujimoto et al., 2023 [47]; Lo et al., 2021 [18]; Atari et al., 2023 [48]; Freeman et al., 2022 [49]; Fumagalli et al. [50], 2022; Plavec et al., 2022 [51]; Lee et al., 2022 [52]; Patterson et al., 2020 [53]; Ulloa et al., 2021 [54]; Li et al., 2023 [55]; Gracheva et al., 2022 [56]; Saito et al., 2021 [57]).

We used ROBINS-I V2 instead of V1 because it improves transparency by refining domains into clearer questions, aligning with randomized trial standards, supporting automation, and harmonizing with RoB 2. Studies showed minimal missing data, and those with critical bias or implausible results were excluded. Certainty was further supported by convergence of diagnostics: reduced τ^2^ and I^2^, robust standard errors, BCa bootstrapped CIs, and consistent findings across all sensitivity models for key predictors like log_dose. After filtering, 16 studies showed excessive heterogeneity as non-comparable experimental designs, or extreme treatment protocols, preventing quantitative pooling; see Figure 1.

We extracted uses of ‘inactivation’ and ‘disinfection,’ categorizing ‘complete inactivation’ definitions inductively by context (e.g., log_10_ thresholds, culture confirmation) from all included studies. Frequency of occurrence and definitional diversity were recorded; see Supporting Information 02, reviewer data extraction notes, and 06, qualitative definitions from included inactivation studies, revealing variability in terminology and a lack of standardized definitions across studies.

## 3. Results

### 3.1. Study Selection

**Figure 2:**
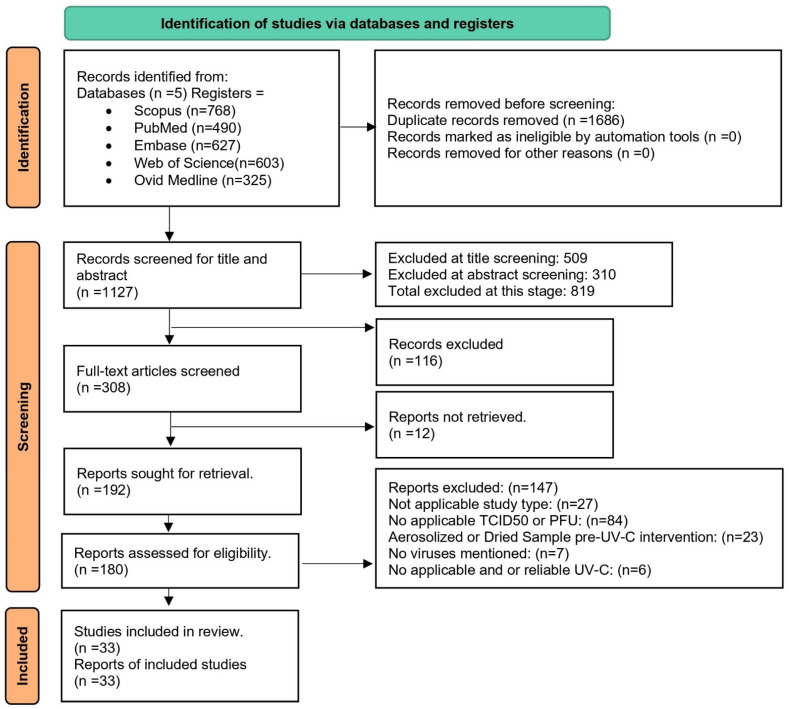
PRISMA Flow Diagram Description [34].

Initially, *n* = 2813 records were identified across five databases, of which 1686 duplicates were removed before screening. The remaining 1127 records underwent title and abstract screening. During this process, 509 records were excluded at the title screening stage, and a further 310 records were excluded following abstract screening, leaving 308 records for subsequent assessment. Of these, 116 records were excluded prior to full-text retrieval, resulting in 192 full-text reports sought for retrieval, of which 12 could not be retrieved. Consequently, 180 studies were assessed for eligibility. Of these, 147 studies were excluded due to an unsuitable study design, lack of relevant viral quantification measures (TCID_50_/PFU), or absence of an applicable and reliable UV-C intervention. The final synthesis included *n* = 33 studies; see Figure 2 [34].

Study-level metadata, PRISMA flow details, and a tabulated record of exclusion rationales are presented in Supporting Information 02, relocated to avoid redundancy and maintain readability.

### 3.2. Study Characteristics and Results of Individual Studies

Our synthesis of proteomic and structural damage patterns across UV-C wavelengths provide the mechanistic basis for the meta-analytic model see Supporting Information 07. Several studies initially meeting the inclusion criteria were later excluded because of experimental deviations, such as dried samples or the application of non-UV-C frequencies [58,59,60,61,62].

In short, low-pressure mercury lamps (LPMLs) were the most frequently applied UV-C source (16 studies), followed by UV-C LEDs and germicidal lamps (5 each). Less common were excimer lamps, UV sterilizing ovens, microplasma UV lamps, medium-pressure mercury lamps, and lasers (1 study each). Direct single-lamp exposure at 253.7 nm predominated. Complex or enclosed multilamp designs have rarely been reported and are generally associated with higher resource requirements or transfusion medicine. Similarly, flow-through reactor systems occur only twice, each involving circulating animal blood [63,64,65,66,67].

Inactivation outcomes varied across studies, similar to findings in research on suspensions, radiation penetration, and viral structure [68,69,70,71,72]. During narrative analysis, we repeatedly found that the 222 nm targeted envelope, 253.7 nm, damaged the genome. The evidence in the included studies suggests that 222 nm radiation poses a lower risk to human exposure and is considered more ‘effective’ than 253.7 nm radiation [73]. 222 nm is reported to induce more extensive structural disruption in pathogens, thereby enhancing inactivation efficacy; however, comprehensive safety evaluations across diverse application contexts remain limited [49,61].

Articles have focused largely on SARS-CoV-2 and surrogates (Phi6, Rotavirus), although susceptibility varies markedly [50,67,74,75]. MS2 bacteriophage studies demonstrated wide variation in UV reactivity even under similar strain and medium conditions [66,76,77,78]. The matrix composition (Phosphate-buffered saline vs. Dulbecco’s Modified Eagle Medium) and stirring affected the apparent susceptibility through absorption and mixing effects [55]. Together, these factors significantly affect the apparent UV susceptibility. Inactivation kinetics predominantly follow biphasic or shoulder-tail models, particularly for viruses exhibiting partial resistance, often inactivated in plasma, highlighting the presence of resistant protective matrices [79,80].

Interestingly, our meta-regression and thematic analyses yielded converging evidence on key predictors of UV-C efficacy. Log_dose emerged as a consistently strong inactivation predictor (β = 3.38, 95% CI [2.95, 3.82], *p* < 0.001, I^2^ = 15.1%), with this relationship remaining robust across all sensitivity tests, including HC3 standard errors and BCa bootstrapping. The UV wavelength significantly moderates the outcomes, with 267 nm (β = 6.42) and 275 nm (β = 3.78) resulting in the highest predicted efficacy and 222 nm resulting in the lowest, whereas 253.7 nm, although most frequently used, was less effective but preserved structural integrity in some cases. At 253.7 nm, damage is localized primarily to nucleic acids via pyrimidine dimer formation [65], whereas 222 nm irradiation produces mainly envelope damage [74,75]. Protein damage and structural deformation depend on the wavelength, fluence, and exposure duration. While UV-based methods can preserve epitope integrity, which is relevant for diagnostic and vaccine contexts, incomplete protein inactivation under various experimental setups limits comparability. In contrast to 222 nm UV-C, 253.7 nm UV-C is advantageous for experimental settings that prefer intact viral structures, such as vaccine development, diagnostic assay design, and pharmaceutical inactivation studies. Although not unheard of, strain-specific differences further influence outcomes, with certain SARS-CoV-2 variants, such as P.1 and Omicron BA.2, showing significantly lower susceptibility than the reference strain USA-WA1/2020 in our analysis; see Table 3 [81,82,83].

### 3.3. Risk of Bias in Studies

The risk of bias was evaluated across seven ROBINS-I V2 domains. Among the 33 nonrandomized intervention studies, 6 were rated “Low,” 12 “Moderate,” 13 “Serious,” and 2 “Critical,” the latter of which were excluded from the synthesis see Supporting Information 08. Critical risk arises from selective reporting and unverifiable outcome measures [84,85]. Most “Serious” ratings stemmed from confounding (Domain 1) and selective reporting (Domain 7). Low-risk studies presented clear methodologies and adequate confounder control, reflecting overall moderate methodological quality. Tabulated domain-specific judgments are relocated due to length.

### 3.4. Results of Syntheses, Certainty of Evidence, and Reporting Biases

**Figure 3:**
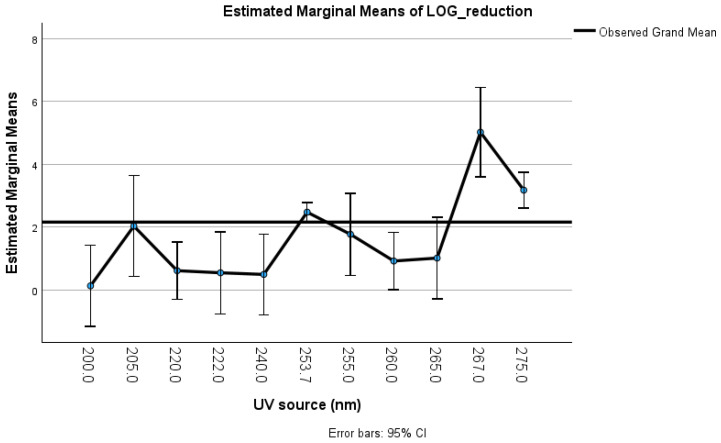
This plot shows estimated marginal means of log_reduction across different UV wavelengths, evaluated at a fixed log_dose (~0.65). Error bars represent 95% confidence intervals. The highest log_reduction is observed near 267 nm, exceeding the overall grand mean line. Covariates in this plot are visualized at the LOG_DOSE value of ≈0.65.

**Figure 4:**
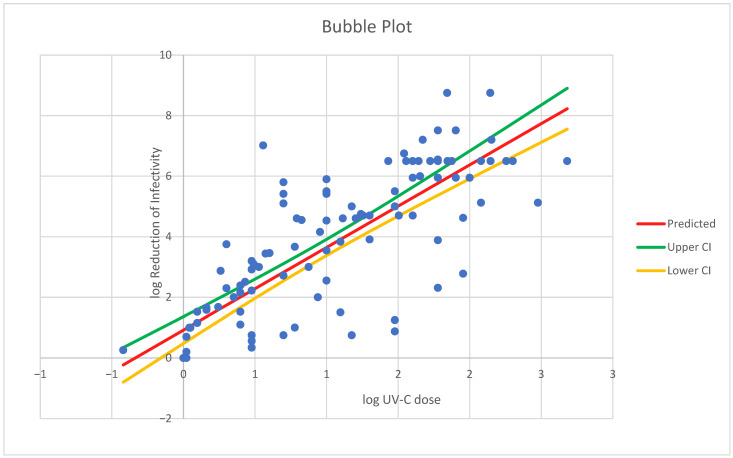
Bubble plot illustrating the dose–response relationship between log-transformed UV dose (LOG_DOSE) and log_10_ viral reduction (LOG_reduction) based on the random-effects meta-regression model. The solid line represents the fitted regression, and the bands indicate the 95% confidence interval around the mean predicted response. A strong and statistically significant positive association is observed (β = 3.38, *p* < 0.001), indicating that increasing UV-C dose is consistently associated with greater viral inactivation.

Meta-regression showed a significant positive dose–response (log_dose β = 2.57, 95% CI [2.29, 2.84], *p* < 0.001) with moderate heterogeneity (I^2^ = 43.5%, τ^2^ = 0.77). After robust filtering, β = 3.38 (95% CI [2.95, 3.82], *p* < 0.001) and I^2^ = 15.1%, indicating improved model stability. The strongest inactivation effects occurred at 267 and 275 nm, with higher doses consistently enhancing log_10_ reduction; see Figure 3 and Figure 4.

Moderator analyses confirmed significant contributions from the virus strain and wavelength. Strains P.1 and Omicron BA.2 presented lower log reductions (β = −3.0 to −4.3, *p* < 0.001), whereas nCoV-FIN-29-Jan-2020 was more susceptible. Several regional strains (e.g., JPN/TY41-702/2022, BRA/SP02 cc/2020, and Liverpool strain/2020) presented reduced susceptibility compared with the USA-WA1/2020 reference strain [47,48,50,51,53,54,85]. However, most Israeli and alpha variants presented nonsignificant differences [53]. These findings persisted under HC3 robust errors and BCa bootstrap resampling (1000 samples). The wavelength effects were strongest at 267 nm (β = 6.42, 95% CI [3.15, 8.85]) and 275 nm (β = 3.78, 95% CI [3.37, 4.29]), *p* < 0.001. The residual heterogeneity decreased from I^2^ = 43.5% to 15.1%, indicating a reliable model fit.

**Figure 5:**
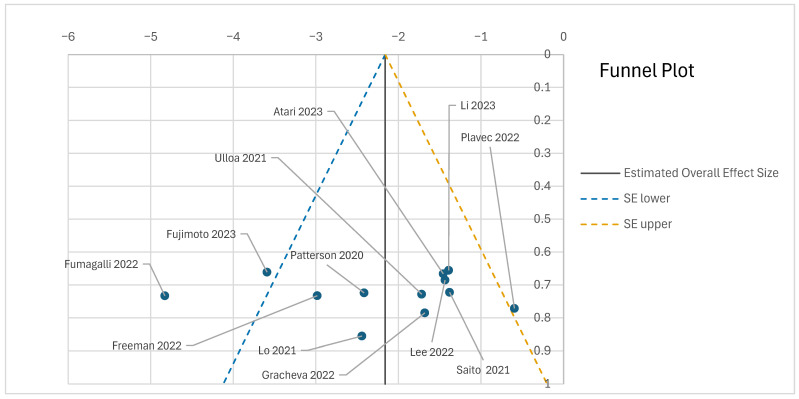
The funnel plot presents the distribution of effect sizes from the 17 SARS-CoV-2-focused studies included in the meta-analysis. The relative symmetry and clustering around the central effect estimate suggest a low risk of publication bias. This supports the robustness of the meta-regression findings regarding UV-C efficacy in viral inactivation. Each point represents an individual study (Fujimoto et al., 2023 [47]; Lo et al., 2021 [18]; Atari et al., 2023 [48]; Freeman et al., 2022 [49]; Fumagalli et al., 2022 [50]; Plavec et al., 2022 [51]; Lee et al., 2022 [52]; Patterson et al., 2020 [53]; Ulloa et al., 2021 [54]; Li et al., 2023 [55]; Gracheva et al., 2022 [56]; Saito et al., 2021 [57]).

The certainty of evidence for subgroup effects was moderate and limited by imprecision and data sparsity at rare wavelengths. Sensitivity and bootstrap analyses showed no effect reversal. Publication bias was minimal (see Figure 5), and reporting bias assessments remained low to moderate.

The corresponding GRADE judgments and evidence profiles appear in Supporting Information 09, which was moved there for conciseness and to avoid duplication of the tabulated criteria. The interaction terms UV-C per dose were significant (F = 10.79, *p* < 0.001; η^2^ = 0.286), indicating structural heterogeneity. According to the GRADE criteria, UV-C disinfection demonstrated strong desirable effects with moderate certainty evidence, notably at 253.7, 267, and 275 nm. Undesirable outcomes (e.g., partial protein alteration) were context-specific and did not offset efficacy in the primary use of viral inactivation. The evidence supports high acceptability and feasibility across clinical and industrial applications.

## 4. Discussion

**Figure 6:**
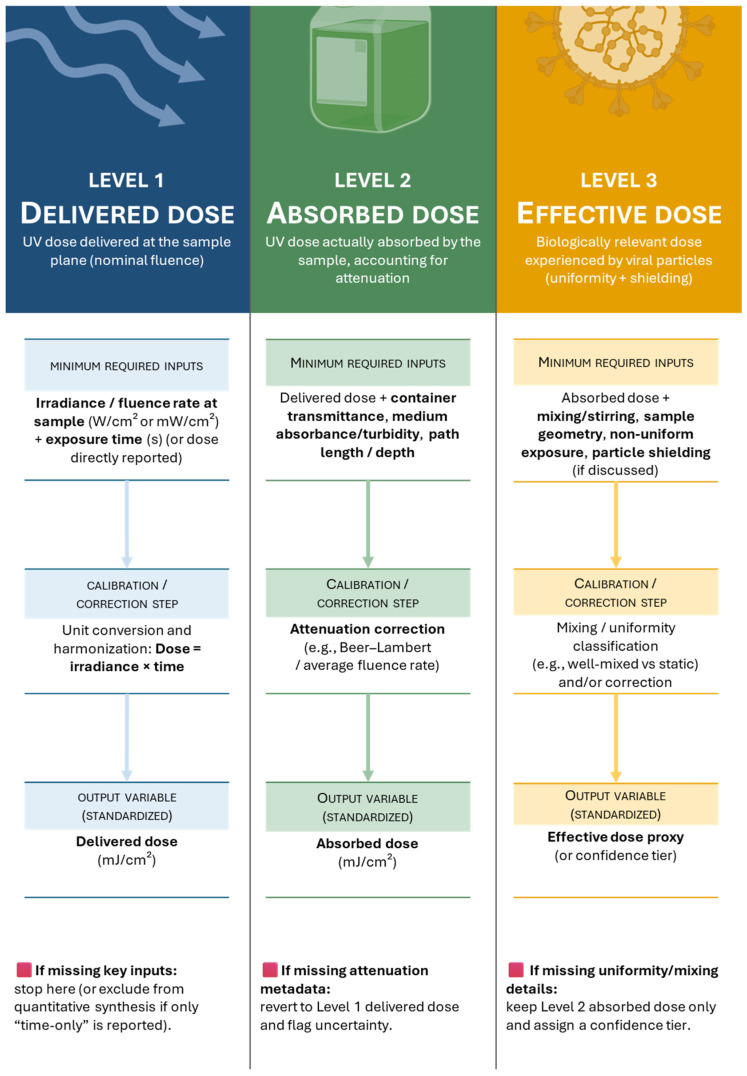
Shows the conceptual hierarchy flowchart of the progression from nominal UV delivered dose to absorbed dose and finally to biologically effective dose, with increasing correction, calibration, and biological relevance at each level.

In essence, our meta-analysis organizes scattered data into a roadmap toward structure-preserving UV inactivation. It maps how the dose, wavelength, medium, and strain influence the effects of UV-C on viruses. On the basis of the data presented, we propose a comprehensive reporting framework, terminology, exposure setups, and dose–response observations; see Table 1. Specifically, we recommend that TCID_50_ assays include well-by-well readouts accompanied by confidence intervals, strictly adhering to the Reed–Muench method, as experiments in some cases neglect such measures [86]. Furthermore, it is crucial to clearly specify whether the reported UV-C dose refers to the delivered dose (emitted by the source), the taken dose (absorbed by the sample), or a nominal value; see Figure 6.

Clear distinctions are essential, as demonstrated in other high-stakes domains, such as aviation, nuclear power, and military operations, where imprecise terminology has historically contributed to catastrophic failures [87,88,89]. We observed that the terms “inactivation” and “disinfection” were used interchangeably. Similarly, a lack of clarity between the aforementioned terms and “complete inactivation” may undermine both biosafety and regulatory reliability. In sum, since viruses do not stop at borders, standardization is vital for translating viral inactivation research into clinical and diagnostic use. The proposed UV-C dose definitions link laboratory protocols to medical standards, ensuring consistent and safe inactivation or disinfection, which is crucial for protecting immunocompromised patients from persistent or potentially inadequately inactivated viruses such as SARS-CoV-2 or Influenza. We propose defining “inactivation” as a treatment that eliminates viral infectivity while preserving structural integrity, enabling its use in downstream and scientific applications. Conversely, “disinfection” refers to making viruses noninfectious without the requirement of maintaining any structural integrity. ‘Complete inactivation’ is defined in the included studies, indicating that detecting the absolute absence of virus is limited by assay sensitivity. To ensure safety, we suggest the use of several types of tests, not just single-log reduction. On the basis of our review, we propose the following definition to make policy-making clearer and easier. “Complete viral inactivation is confirmed when no infectious virus is detectable via validated, highly sensitive infectivity assays, and the observed log reduction matches or at least theoretically exceeds the initial viral titer, typically verified through multipassage cell culture methods on one pathogen.” In addition, the difference between complete inactivation and disinfection is that complete inactivation may intentionally preserve viral structure or some function in one given pathogen, and disinfection ensures that only viruses or pathogens are noninfectious, often by using high doses and other complementary methods to eliminate multiple pathogens. We believe that this definition is rigorous and built upon the WHO standards [90,91,92,93,94,95,96]. Evidently, this definition is inherently tied to the specific cell line, viral strain, and assay system used, and without in vivo confirmation (e.g., animal model testing), “complete inactivation” remains a high-confidence prediction in this context rather than absolute proof.

The implementation of UV-C-based viral inactivation strategies spans three practical domains, namely, laboratory research, clinical and healthcare settings, and domestic or decentralized environmental use, each with distinct methodological and regulatory requirements.

In laboratory and research environments, inactivation protocols play a pivotal role in enabling the transition of high-risk pathogens from BSL-3 or BSL-4 containment to lower biosafety levels (BSL-2 or BSL-1) for downstream analysis, assay development, or emergency response. However, this transition hinges entirely on rigorously validated inactivation methods. If UV-C treatment protocols are not adequately validated via statistically sound, sensitive, and strain-specific assays, the consequences may not merely be experimental artifacts but also potential unintentional release of infectious agents. As emphasized in BMBL-6, failure to confirm complete inactivation under defined, reproducible conditions risks exposing laboratory personnel, the surrounding environment, and potentially the public to high-consequence pathogens [97]. To mitigate this, we propose that inactivation curves must include at least five data points and that TCID_50_ assays must be conducted with appropriate multipassage workflows and reported confidence intervals, strictly following the Reed–Muench method. For SARS-CoV-2, conclusions based on surrogates must be cautiously applied, as structural and genomic differences can significantly alter inactivation kinetics. Furthermore, the wavelength effects and log_dose relationships reported here may serve as starting points for engineering controls in environmental and hospital liquids, but they are to be recalibrated with matrix-specific attenuation, hydrodynamics, and safety margins before deployment.

In healthcare and patient care settings, where UV-C disinfection is increasingly employed in heating, ventilation, and air conditioning systems, intensive care units, surgical units, and point-of-care sterilization tools, the priority shifts to standardized log-reduction performance, safety margins, and regulatory adherence. Here, mechanistic clarity is secondary to outcome robustness; however, without unified terminology and operational guidance, safety margins may be misapplied or misunderstood. Distinctions in terminology (“inactivation”, “disinfection”, complete inactivation”) became relevant in light of even strain-specific resistance differences, such as those observed in SARS-CoV-2 P.1 and Omicron BA.2, where expected efficacy may be reduced. Our meta-analysis showed that the 267 nm and 275 nm wavelengths were associated with the highest viral inactivation potential (β = 6.4218 and 3.7876, respectively) for SARS-CoV-2 in liquids, but without clear definitions and robust assay reporting, such findings may be misinterpreted or overgeneralized. Our review highlights that UV-C dose–response data, although largely derived from controlled liquid matrices, are highly relevant to blood safety research, where optical density, protein content, and chromophores create complex irradiation dynamics. Findings such as the structural preservation observed at 253.7 nm and the high efficacy of 267–275 nm for SARS-CoV-2 indicate that blood treatment protocols for humans or animals require careful wavelength calibration to balance inactivation with biomolecule integrity. At 253.7 nm, UV-C could enhance convalescent plasma therapy by achieving complete viral inactivation with minimal protein alteration. Unlike UVA/UVB–riboflavin systems such as Mirasol (a commercially available UVA/UVB–riboflavin pathogen-reduction system, Mirasol Pathogen Reduction Technology; Terumo Blood and Cell Technologies, Lakewood, CO, USA), which induce structural and functional protein changes, the distinct absorption profile of UV-C limits such effects, potentially preserving protein integrity and improving transfusion efficacy and safety. A detailed characterization of the optical and biochemical properties of blood, informed by this synthesis, may guide future studies to optimize UV-C systems for transfusion safety, zoonotic risk reduction, and pathogen control; see Table 4 [98,99,100].

In domestic and environmental settings, such as air purifiers, food disinfectors, and water systems, UV-C is often used without regulation or training, posing a public risk when it is marketed as a universally effective “sterilizer” without context or safety warnings. Additionally, it is plausible that the perceived safety of 222 nm may stem partly from an underestimation of its potential risks to human tissue, given its pronounced biocidal activity, which we recognize contrasts with the literature on the subject [73,101,102,103]. Upper-air systems, handheld UV wands, and low-quality consumer devices risk carcinogenic UV-C exposure and often fail to inactivate pathogens due to improper doses, wavelength mismatches, or lamp degradation. In food and water systems, poor calibration can harm nontarget microbes or degrade product quality if inactivation is not balanced with environmental impact. Advertising UV-C devices with claims such as “99.99% virus reduction” (a 4-log reduction) can be misleading, especially in high-titer scenarios. For viruses commonly present in human secretions at concentrations of 10^6^–10^8^ TCID_50_/mL, norovirus in stool, influenza A nasal swabs, or SARS-CoV-2 (nasopharyngeal secretions), a 4-log reduction would still leave 10^2^–10^4^ TCID_50_/mL of infectious material [104,105,106]. This residual viral load can still cause infection, especially for pathogens with low infectious doses or in immunocompromised individuals. Disinfection claims should therefore reflect realistic starting titers, pathogen-specific infectivity thresholds, and adequate safety margins for each application.

Our systematic review and meta-analysis faced several limitations due to the abovementioned research gaps and experimental setups [6,13,107]. Although a dual-reviewer system with consensus was used, manual data extraction and conversion, especially for dose and titer standardization, carried a risk of human error, which we mitigated through repeated expert consultation. Furthermore, the inclusion criterion limited the dataset to peer-reviewed publications, excluding gray literature and preprints that might contain useful findings. While the exclusion of grey literature was intended to enhance methodological rigor and minimize the influence of non-peer-reviewed evidence, this decision may have led to the omission of relevant emerging data and is therefore acknowledged as a potential limitation. Despite narrative, thematic, and meta-regression, the limited granularity in the original data constrained the depth of subgroup or stratified analyses. At 253.7–275 nm, UV-C is effective under controlled conditions but requires standardized protocols to ensure reproducibility across laboratory and real-world applications [57]. In spite of the use of robust methods such as bootstrapping and bias-adjusted regression, further model refinement would have required the exclusion of valuable data, limiting analytical completeness and potential overfitting. In the present analysis, treating these differences as residual heterogeneity was necessary to avoid overfitting, given sparse data across medium types. However, this limitation highlights the need for future studies, including our own, to explicitly quantify and standardize medium effects, which will allow a clearer attribution of variability to viral susceptibility. Future research should focus on refining predictive models and validating the safety and effectiveness of 222 nm. A global reporting framework, akin to MIQE or CONSORT, outlined in our proposed table, could significantly enhance data comparability and translational value. Our framework offers a reproducible structure and policy-ready definitions for standardizing UV-C protocols. Although dose–response trends may appear linear, in truth, inactivation kinetics often follow biphasic or sigmoid curves, limiting the validity of log-linear assumptions and potentially skewing predictive models. Future work should account for these nonlinear dynamics. Filtering for methodological quality substantially improved model performance and showed that experimental inconsistency was the main source of bias and variability in the unfiltered dataset. Many studies lack sufficient data points (<5 per dose–response curve), hindering accurate kinetic modeling. Furthermore, we did not compare UV-C susceptibility across virus types because the available non-SARS-CoV-2 data were sparse and inconsistent, and we identify standardized, wavelength-resolved datasets across virus families as a key need for future studies. These findings underscore the necessity for standardization.

## 5. Conclusions

Our study clarifies how wavelength-specific mechanisms and dose–response behavior drive UV-C inactivation in liquids, explaining prior inconsistencies and demonstrating why reproducibility has remained limited. By showing that 253.7 nm could preserve structural integrity while 267–275 nm achieves peak inactivation efficiency, it provides the first evidence-based framework for selecting wavelengths according to biosafety or research needs. Future work could therefore verify structural integrity using high-resolution techniques such as cryo-electron microscopy or mass spectrometry. Across all domains, the lack of standardized terminology, inconsistent reporting of UV-C dose and wavelength, and improper microbial assay use undermine both scientific credibility and public safety, and our reporting framework and proposed terminology address these challenges by enabling biosafety-compliant inactivation, reducing exposure risks, and promoting harmonization in healthcare and related fields. The identified wavelength-specific dose–response relationships clarify SARS-CoV-2 inactivation and provide quantitative parameters for optimizing efficiency and energy use, while maintaining the need for recalibration to specific applications. Verification of structural integrity using complementary techniques such as cryo-electron microscopy or mass spectrometry would be a valuable direction for future mechanistic studies and is an excellent recommendation that we will explicitly highlight as a research gap and future perspective in relevant UV inactivation studies. Most importantly, this work creates a new research pathway by delivering an integrated, wavelength-resolved roadmap that allows precise design of future structure-preserving UV-C protocols, enabling studies that were previously scarce in number and reproducibility due to fragmented and incompatible datasets.

## Figures and Tables

**Table 1:** Showing the developed PICO framework for study retrieval with MeSH.

Question form: PICO	Keywords	Reasons
Population (P):	“viruses” [MeSH Terms] OR virus [Text Word]	RNA and or DNA viruses and their families
Intervention (I):	“Ultraviolet Rays” [Mesh] OR UV [All Fields]	Exposure to UV-C radiation within the 100–280 nm spectrum.
Comparison (C):	Adding keywords here, referring to the operational parameters, would encumber the search and render it ineffective.	Different operational parameters, such as the frequency of UV-C radiation application, the duration of exposure, and the openness (possibly referring to the physical openness or containment of the system where the UV-C radiation is applied).
Outcomes (O):	Virus Inactivation” [Mesh] OR “inactivation” OR “disinfection” [MeSH Terms] OR disinfection [Text Word].	The primary outcomes to be assessed are disinfection and inactivation of the viruses. Yes or no, which word does the text use, is there any distinction? To what degree was this successful?

**Table 2:** Shows Extracted Data items and suggested table for follow-up studies based on inductive and deductive coding exported from MS Forms.

Extracted Data (Not a Real Record)	Example (Not a Real Record)
Virus Name	SARS-CoV-2
Virus Strain	2019-nCoV/Italy-INMI1
Genome size (bp, nt) https://www.ncbi.nlm.nih.gov/genbank/ (accessed on 20 September 2025)	29,740 bp
Nucleic Acid Type GenBank Overview (nih.gov)	ssRNA+
Enveloped	Yes
Baseline Viral Load TCID50 or Baseline Viral PFU	20,000,000
Open/Contained SystemOpen: Petri dish, microplate 24-well plate, 96-well plate. Closed: centrifuge tube, Eppendorf, etc.	Open
Sample Volume (mL)	1
Sample Type	VERO E6 supernatant
Suspension Medium, Buffer solution type, UV transmission depth	Phosphate-buffered saline
Sample Temperature (°C) (when irradiated)	25
UV-C Wavelength (nm)	253.7
UV Light Source Type	UV-C LED
UV Exposure Configuration, Optical Pathway Description	Direct Exposure (Single Lamp)
Nucleic Acid Damage Evidence	(Y/N + Description)
Lamp to Sample Distance (mm)	1
UV Light Source Performance (mW)	13,000
UV Light Intensity Minimum (W/cm^2^)	0.0005
UV Light Intensity Maximum (W/cm^2^)	0.0013
Exposure Duration Minimum (s)	1
Exposure Duration Maximum (s)	60
UV Dose Specification Minimum (mJ/cm^2^)	5
The absorbed dose accounts for the UV-C energy actually taken up by the medium, considering attenuation due to container materials, solution turbidity, and path length. (mJ/cm^2^). Additionally, the liquid medium after accounting for attenuation by container walls, medium turbidity, path length, and spectral absorbance. Should be supported by absorption spectra (100–280 nm) of the medium to quantify wavelength-specific transmission.	
The delivered dose refers to the total UV-C energy emitted by the light source, typically measured at the source output. (mJ/cm^2^)	
The effective dose represents the biologically active UV-C energy that reaches and inactivates pathogens, influenced by factors like mixing, solution composition, and shielding by suspended particles. (mJ/cm^2^)	
Standard Deviation, Mean Difference in TCID50 and or PFU	±4
Minimum Log Rate of Virus Inactivation	1
Minimum % of Virus Inactivation	90
UV Dose Specification Maximum (mJ/cm^2^)	100
Maximum Log Rate of Virus Inactivation	7
Maximum % of Virus Inactivation	99.99999
Stirring, Mixing parameters e.g., method, agitation speed	Magnetic Stirring
Method of Dose Quantification	
Observed Proteomic	(Y/N + Description)

**Table 3:** Summary of wavelength-dependent targets and dominant mechanisms of UV-mediated viral inactivation, highlighting differences in molecular damage profiles across commonly used UV-C and far-UV wavelengths based on the reviewed literature.

UV Wavelength	Dominant Target(s)	Reported Mechanistic Features	Key References
222 nm (far-UVC)	Viral envelope/structural proteins	Predominantly induces structural and envelope damage; enhanced surface disruption with limited penetration; associated with lower human exposure risk but limited comprehensive safety data	[49,61,73,74,75]
253.7 nm (conventional UV-C)	Viral nucleic acids	Direct genomic damage via pyrimidine dimer formation; replication inhibition; may preserve overall virion structure under certain conditions	[65,68,69,70,71,72]
267–275 nm	Mixed (nucleic acids ± proteins)	High inactivation efficacy observed; wavelength-dependent balance between genomic damage and protein effects;	[50,67,74,75]

**Table 4:** Comparison flowchart of current UV-C (253.7 nm) and the Mirasol (UVA + riboflavin) system with respect to clinical translational feasibility and implementation constraints in blood pathogen inactivation.

Aspect	UV-C (253.7 nm)	Mirasol (UVA + Riboflavin)
**Primary mechanism**	Direct nucleic acid damage (pyrimidine dimer formation)	Photoactivation of riboflavin causing nucleic acid damage
**Wavelength/reagent requirement**	253.7 nm UV-C, no chemical additives	UVA (≈365 nm) + riboflavin
**Viral spectrum (enveloped/non-enveloped)**	Broad, strong for enveloped viruses; variable for non-enveloped	Broad, including enveloped and some non-enveloped viruses
**Bacterial reduction**	Effective for many bacteria	Effective
**Parasite reduction**	Limited data; wavelength-dependent	Demonstrated
**Penetration in blood products**	Strongly limited by hemoglobin absorption and scattering	Improved penetration due to longer wavelength
**Photochemical residuals**	None	Riboflavin remains (clinically accepted)
**Effect on platelets**	Potential activation or functional impairment at higher doses	Clinically validated preservation
**Effect on coagulation factors**	Dose-dependent degradation possible	Mild to moderate reduction (product-dependent)
**Uniform dose delivery in bags**	Challenging without thin-layer or mixing systems	Validated illumination geometry
**Cost per unit (OPEX)**	Potentially low per unit, device-dependent	Higher per-unit cost
**Capital expenditure (CAPEX)**	Moderate–high (custom UV systems required)	High (proprietary system)
**Scalability/throughput**	Limited by optical constraints and engineering solutions	Established for routine blood bank use
**Regulatory maturity**	Experimental/preclinical	Approved and widely implemented
**Clinical adoption level**	Proof-of-concept/early translational stage	Routine clinical use

## Data Availability

The extracted data used in this systematic review and meta-analysis, including study characteristics, viral strains, UV-C parameters, and outcome measures, as well as the template data collection forms, analysis datasets, and analytic code, are openly available in Figshare at https://doi.org/10.6084/m9.figshare.30024085. Supporting Information is in accordance with FAIR principles. The following files are available in open repositories: Supplementary Methods and Data Extraction Tables (PDF): Detailed description of the search strategies, inclusion/exclusion criteria, full PICO framework, and extracted study characteristics. Quality Assessment Tables (ROBINS-I V2, GRADE, MECIR) (PDF): Risk of bias ratings, certainty of evidence grading, and methodological quality assessment figures. Statistical Outputs and Additional Figures (Word): Meta-regression model outputs, subgroup analyses, residual heterogeneity plots, and forest plots.

## Supplementary Materials

The following supporting information can be downloaded at: https://www.mdpi.com/article/10.3390/v18030276/s1, the extracted data used in this systematic review and meta-analysis, including information on study characteristics, viral strains, UV-C parameters, and outcome measures, are available. Supporting Information 01 contains the full database search strategies; 02 details the screening process and exclusion table; 03 describes data harmonization and conversion rules; 04 provides the cleaned analysis dataset; 05 reports the qualitative text analysis results; 06 compiles extracted terminology definitions; 07 summarizes wavelength-specific mechanistic findings; 08 presents the ROBINS-I V2 risk-of-bias assessments; 09 includes the GRADE recommendtations. The template data collection forms extracted from the included studies, analysis datasets, analytic codes, and all other review-related materials are available at https://doi.org/10.6084/m9.figshare.30024085 (accessed on 20 September 2025).

## Abbreviations

The following abbreviations are used in this manuscript:

**Abbreviation (EN)**	**Full Term**	**Meaning**
UV-C	Ultraviolet-C	Electromagnetic radiation in the 100–280 nm range
PFU	Plaque-forming unit	Quantitative measure of infectious viral particles
TCID50	Tissue culture infectious dose 50%	Viral quantity needed to infect 50% of cultured cells
BSL	Biosafety level	Classification of laboratory safety standards
PRISMA	Preferred Reporting Items for Systematic Reviews and Meta-Analyses	Guidelines for reporting systematic reviews
MECIR	Methodological Expectations of Cochrane Intervention Reviews	Standards for Cochrane review conduct and reporting
GRADE	Grading of Recommendations Assessment, Development, and Evaluation	System for rating certainty of evidence and strength of recommendations
ROBINS-I V2	Risk Of Bias In Non-randomized Studies of Interventions, version 2	Tool for assessing risk of bias in non-randomized intervention studies
CI	Confidence interval	Statistical range expressing uncertainty around an estimate
LED	Light-emitting diode	Semiconductor light source
DNA	Deoxyribonucleic acid	Genetic material in organisms
RNA	Ribonucleic acid	Molecule essential for coding, decoding, and expression of genes
