# Supplementary material for: Wavelength-Specific UV-C Inactivation of Viruses in Liquids: Dose–Response, Mechanistic Insights, and Structural Integrity—A Systematic Review and Meta-Analysis
Source: Viruses. 2026 Feb 24;18(3):276. doi: 10.3390/v18030276 (PMC13030338; doi:10.3390/v18030276)
Supplement: Supplementary file 1 [file viruses-18-00276-s001.zip › 01_Detailed_Database_Search_Terms.pdf]

Shows searched databases, retrieved results, and adapted query strings to databases

**Database**	**MeSH and Retrieved Studies**	**Adapted Query String**
Pubmed	(MeSH Applied, 490 results)	(“Virus Inactivation” [MeSH Terms] OR “inactivation” [Text Word] OR “Disinfection”[MeSH Terms] OR “disinfection”[Text Word]) AND (“Ultraviolet Rays”[MeSH Terms] OR “UV”[All Fields]) AND (“Viruses”[MeSH Terms] OR “virus”[Text Word])
Embase	(MeSH applied, 627 results)	('virus inactivation'/exp OR 'inactivation':ab,ti OR 'disinfection'/exp OR 'disinfection':ab,ti) AND ('ultraviolet rays'/exp OR 'uv':ab,ti) AND ('viruses'/exp OR 'virus':ab,ti) AND [2019-2024]/py AND [english]/lim
Scopus	(MeSH not applicable, 768 results)	(TITLE-ABS-KEY (“virus inactivation”) OR TITLE-ABS-KEY (inactivation) OR TITLE-ABS-KEY (“disinfection”)) AND (TITLE-ABS-KEY (“ultraviolet rays”) OR TITLE-ABS-KEY (uv)) AND (TITLE-ABS-KEY (viruses) OR TITLE-ABS-KEY (virus)) AND PUBYEAR > 2019 AND PUBYEAR < 2024 AND (LIMIT-TO (LANGUAGE, “English”))
Web of Science	(MeSH not applicable, 603 results)	(TS= (“virus inactivation” OR inactivation OR “disinfection”)) AND (TS= (“ultraviolet rays” OR UV)) AND (TS= (viruses OR viruses))
Ovid Medline and Journals@OVID full text	(MeSH applied, 325 results)	(“Virus Inactivation”/ or inactivation.tw. or “Disinfection”/ or disinfection.tw.) AND (“Ultraviolet Rays”/ or UV.tw.) AND (“Viruses”/ or virus.tw.)
