# Supplementary material for: Wavelength-Specific UV-C Inactivation of Viruses in Liquids: Dose–Response, Mechanistic Insights, and Structural Integrity—A Systematic Review and Meta-Analysis
Source: Viruses. 2026 Feb 24;18(3):276. doi: 10.3390/v18030276 (PMC13030338; doi:10.3390/v18030276)
Supplement: Supplementary file 1 [file viruses-18-00276-s001.zip › 02_Data_Extraction_Template_MSForms.pdf]

04_form_UV review

Spectral Efficacy: The Role of UV-C Light Frequency in Viral Inactivation and Disinfection

* Required

* This form will record your name, please fill your name.

Title

1. Title *

- 1. Advancements in 275 nm UV-LED Technology for Deactivation of Bacteriophages, Phi6 and MS2; 10.1109/HONET59747.2023.10374888
- 2. Comparison of the inactivation capacity of various UV wavelengths on SARS-CoV-2; 10.1016/j.bbrep.2022.101379
- 3. Demonstration of antiviral activity of far-uvc microplasma lamp irradiation against sars-cov-2; 10.7754/Clin.Lab.2020.201140
- 4. Determination of the characteristic inactivation fluence for SARS-CoV-2 under UV-C radiation considering light absorption in culture media; 10.1038/s41598-021-94648-w
- 5. Determination of the UV Inactivation Constant under 280 nm UV LED Irradiation for SARS-CoV-2; 10.1111/php.13653
- 6. Disinfection of SARS-CoV-2 by UV-LED 267Â nm: comparing different variants; 10.1038/s41598-023-35247-9
- 7. Dose-Response Behavior of Pathogens and Surrogate Microorganisms across the Ultraviolet-C Spectrum: Inactivation Efficiencies, Action Spectra, and Mechanisms; 10.1021/acs.est.3c00518
- 8. Effect of inactivating RNA viruses by coupled UVC and UVA LEDs evaluated by a viral surrogate commonly used as a genetic vector; 10.1364/BOE.468445
- 9. Effect of inactivation methods on sars-cov-2 virion protein and structure; 10.3390/v13040562
- 10. Effect of intermittent irradiation and fluence-response of 222 nm ultraviolet light on SARS-CoV-2 contamination; 10.1016/j.pdpdt.2021.102184
- 11. Effect of spray-drying and ultraviolet C radiation as biosafety steps for CSFV and ASFV inactivation in porcine plasma; 10.1371/journal.pone.0249935
- 12. Effect of Ultraviolet Light C (UV-C) Radiation Generated by Semiconductor Light Sources on Human Beta-Coronaviruses' Inactivation; 10.3390/ma15062302
- 13. Effective inactivation of Nipah virus in serum samples for safe processing in low-containment laboratories; 10.1186/s12985-020-01425-8
- 14. Effectiveness of 222-nm ultraviolet light on disinfecting SARS-CoV-2 surface contamination; 10.1016/j.ajic.2020.08.022
- 15. Efficiency improvement of AlGaN-based deep-ultraviolet light-emitting diodes and their virus inactivation application; 10.35848/1347-4065/ac10f2
- 16. Enhanced viral inactivation by combined ultraviolet light and heat; 10.1088/1612-202X/ac52bb
- 17. Evaluation of disinfection efficacy of single UV-C, and UV-A followed by UV-C LED irradiation on Escherichia coli, B. spizizenii and MS2 bacteriophage, in water; 10.1016/j.scitotenv.2022.160256

- 18. Evaluation of the effectiveness of the surepure turbulator ultraviolet-C irradiation equipment on inactivation of different enveloped and non-enveloped viruses inoculated in commercially collected liquid animal plasma; 10.1371/journal.pone.0212332
- 19. Fabrication of Ag-doped ZnO/PAN composite nanofibers by electrospinning: Photocatalytic and antiviral activities; 10.1007/s11814-021-1037-4
- 20. Focused multivector ultraviolet (FMUV) technology rapidly eradicates SARS-CoV-2 in-vitro: Implications for hospital disinfection of COVID-19 environments; 10.1016/j.ajic.2022.02.001
- 21. Handheld Multispectral Fluorescence Imaging System to Detect and Disinfect Surface Contamination; 10.3390/s21217222
- 22. Hepatitis E virus is effectively inactivated by methylene blue plus light treatment; 10.1111/trf.17108
- 23. Hepatitis E virus is effectively inactivated in platelet concentrates by ultraviolet C light; 10.1111/vox.12936
- 24. Immunogenic properties of SARS-CoV-2 inactivated by ultraviolet light; 10.1007/s00705-022-05530-7
- 25. In Vitro Virucidal Effects of Ultraviolet Light Prototypes on RNA viruses; https://hdl.handle.net/11573/1681572
- 26. Inactivation and genome damage of rotavirus and a human norovirus surrogate by monochloramine treatment and sequential application with UV; 10.1016/j.watres.2022.119309
- 27. Inactivation of HCoV-NL63 and SARS-CoV-2 in aqueous solution by 254 nm UV-C; 10.1016/j.jphotobiol.2023.112755
- 28. Inactivation of health-related microorganisms in water using UV light-emitting diodes; 10.2166/ws.2019.022
- 29. Inactivation of Japanese encephalitis virus in plasma by methylene blue combined with visible light and in platelet concentrates by ultraviolet C light; 10.1111/trf.16021
- 30. Inactivation of Material from SARS-CoV-2-Infected Primary Airway Epithelial Cell Cultures; 10.3390/mps4010007
- 31. Inactivation of phage phiX174 by UV(254) and free chlorine: Structure impairment and function loss; 10.1016/j.jenvman.2023.117962
- 32. Inactivation of SARS-CoV-2 and COVID-19 Patient Samples for Contemporary Immunology and Metabolomics Studies; 10.4049/immunohorizons.2200005
- 33. Inactivation of SARS-CoV-2 infectivity in platelet concentrates or plasma following treatment with ultraviolet C light or with methylene blue combined with visible light; 10.1111/trf.17238
- 34. Inactivation of SARS-CoV-2 isolates from lineages B.1.1.7 (Alpha), P.1 (Gamma) and B.1.110 by heating and UV irradiation; 10.1016/j.jviromet.2021.114216
- 35. Inactivation of severe fever with thrombocytopenia syndrome virus for improved laboratory safety; 10.1016/j.jobb.2020.02.002

- 36. Inactivation of three emerging viruses â€" severe acute respiratory syndrome coronavirus, Crimeanâ€"Congo haemorrhagic fever virus and Nipah virus â€" in platelet concentrates by ultraviolet C light and in plasma by methylene blue plus visible light; 10.1111/vox.12888
- 37. Inactivation of yellow fever virus in plasma after treatment with methylene blue and visible light and in platelet concentrates following treatment with ultraviolet C light; 10.1111/trf.15332
- 38. Irradiation by a Combination of Different Peak-Wavelength Ultraviolet-Light Emitting Diodes Enhances the Inactivation of Influenza A Viruses; 10.3390/microorganisms8071014
- 39. Mechanism and efficacy of virus inactivation by a microplasma UV lamp generating monochromatic UV irradiation at 222 nm; 10.1016/j.watres.2020.116386
- 40. Mechanisms of SARS-CoV-2 Inactivation using UVC Laser Radiation; 10.1101/2023.02.03.526944
- 41. Methods of Inactivation of SARS-CoV-2 for Downstream Biological Assays; 10.1093/infdis/jiaa507
- 42. Monitoring SARS-CoV-2 decontamination by dry heat and ultraviolet treatment with a swine coronavirus as a surrogate; 10.1016/j.infpip.2020.100103
- 43. New ultraviolet C light-based method for pathogen inactivation of red blood cell units; 10.1111/trf.17098
- 44. Optimized parameters for effective SARS-CoV-2 inactivation using UVC-LED at 275Â nm; 10.1038/s41598-022-20813-4
- 45. Pathogen Inactivation in Drinking Water: A Point-of-Use Microscale Reactor with Ultraviolet Irradiation; 10.1089/ees.2021.0468
- 46. Pathogen inactivation of red blood cells by ultraviolet C light; 10.1111/vox.12792
- 47. Photoinactivation of the bacteriophage PhiX174 by UVA radiation and visible light in SM buffer and DMEM-F12; 10.1186/s13104-023-06658-8
- 48. Rapid and complete inactivation of SARS-CoV-2 by ultraviolet-C irradiation; 10.1038/s41598-020-79600-8
- 49. Rapid inactivation of sars-cov-2 variants by continuous and intermittent irradiation with a deep-ultraviolet light-emitting diode (Duv-led) device; 10.3390/pathogens10060754
- 50. Rapid inactivation of SARS-CoV-2 with Deep-UV LED irradiation; 10.1080/22221751.2020.1796529
- 51. SARS-CoV-2 Production, Purification Methods and UV Inactivation for Proteomics and Structural Studies; 10.3390/v14091989
- 52. SARS-CoV-2 variants inactivation of plasma units using a riboflavin and ultraviolet light-based photochemical treatment; 10.1016/j.transci.2022.103398
- 53. Stability of SARS-CoV-2 and other airborne viruses under different stress conditions; 10.1007/s00705-021-05293-7

- 54. Structural and Immunoreactivity Properties of the SARS-CoV-2 Spike Protein upon the Development of an Inactivated Vaccine; 10.3390/v15020480
- 55. Superiority of UV222 radiation by in situ aquatic electrode KrCl excimer in disinfecting waterborne pathogens: Mechanism and efficacy; 10.1016/j.jhazmat.2023.131292
- 56. Susceptibility of enveloped and non-enveloped viruses to ultraviolet light-emitting diode (UV-LED) irradiation and implications for virus inactivation mechanisms; 10.1039/d3ew00277b
- 57. Susceptibility of SARS-CoV-2 to UV irradiation; 10.1016/j.ajic.2020.07.031
- 58. Systematic evaluating and modeling of SARS-CoV-2 UVC disinfection; 10.1038/s41598-022-09930-2
- 59. Tailored hybrid microbial water disinfection system using sequentially assembled microbial fuel cells and an ultraviolet C light-emitting diode; 10.1016/j.watres.2023.120482
- 60. Ultraviolet-C light at 222 nm has a high disinfecting spectrum in environments contaminated by infectious pathogens, including SARS-CoV-2; 10.1371/journal.pone.0294427
- 61. UV 254 nm is more efficient than UV 222 nm in inactivating SARS-CoV-2 present in human saliva; 10.1016/j.pdpdt.2022.103015
- 62. UV Disinfection of Human Norovirus: Evaluating Infectivity Using a Genome-Wide PCR-Based Approach; 10.1021/acs.est.9b05747
- 63. UV Inactivation of Rotavirus and Tulane Virus Targets Different Components of the Virions; 10.1128/AEM.02436-19
- 64. UV Inactivation of SARS-CoV-2 across the UVC Spectrum: KrCl* Excimer, Mercury-Vapor, and Light-Emitting-Diode (LED) Sources; 10.1128/AEM.01532-21
- 65. UV inactivation of viruses in water: Its potential to mitigate current and future threats of viral infectious diseases; 10.35848/1347-4065/ac2b4f
- 66. UV radiation sensitivity of bacteriophage PhiX174-A potential surrogate for SARS-CoV-2 in terms of radiation inactivation; 10.3934/microbiol.2023023
- 67. UVC disinfects SARS-CoV-2 by induction of viral genome damage without apparent effects on viral morphology and proteins; 10.1038/s41598-021-93231-7
- 68. UVC inactivation of MS2-phage in drinking water â€“ Modelling and field testing; 10.1016/j.watres.2021.117496
- 69. UV-C irradiation is highly effective in inactivating SARS-CoV-2 replication; 10.1038/s41598-021-85425-w
- 70. UV-C light-based surface disinfection: Analysis of its virucidal efficacy using a bacteriophage model; 10.1055/s-0042-1746562
- 71. Viral Inactivation with Emphasis on SARS-CoV-2 Using Physical and Chemical Disinfectants; 10.1155/2021/9342748

- 72. Wavelength dependence of ultraviolet light inactivation for SARS-CoV-2 omicron variants; 10.1038/s41598-023-36610-6

2. Study Type *

- **Randomized Controlled Trial**: A study in which participants are randomly assigned to receive one of several clinical interventions. One of these interventions is the standard of comparison or control.
- **Non-randomized Studies—of Interventions**: Studies that assess the effects of interventions without the use of random assignment. These can include observational studies, cohort studies, and case-control studies.
- **Non-randomized Studies—of Exposures**: Research focused on the effects of exposures or risk factors without randomization. This includes cohort, case-control, and cross-sectional studies.
- **Systematic Reviews**: Instruments and criteria used to evaluate the quality and findings of systematic reviews.
- **Animal Laborytory Studies**: Research conducted on animal subjects in a laboratory setting to understand biological and physiological processes or to test interventions before human trials.
- Other

3. Year of Publication *

The value must be a number

4. Journal Name *

- Scientific Reports
- Photochemistry and Photobiology
- Korean Journal of Chemical Engineering
- Other

5. Journal Classification Quartile (Q) or (D) (C) Rating *

https://www.mtmt.hu/folyoiratok

- D1
- Q1
- Q2
- Q3
- Other

Virus Details

6. Virus Name

eg. SARS-CoV-2

7. Virus Strain

eg. 2019-nCoV/Italy-INMI1 NR-52284

8. Genome size (bp, nt)

Based on virus strain from: https://www.ncbi.nlm.nih.gov/genbank/

9. Nucleic Acid Type

GenBank Overview (nih.gov)

- ssRNA (+)
- ssRNA (-)
- dsRNA
- ssDNA
- dsDNA
- ssRNA-RT
- RNA-RT

10. Enveloped

- Yes
- No

11. Baseline Viral Load TCID50

Extract the value and unit of TCID50/mL from the given text prior to UV-C Intervention (Eg.: 2x10^7= 20 000 000)

The value must be a number

12. Baseline Viral PFU

Extract the value and unit of PFU/mL from the given text prior to UV-C Intervention (Eg.: 2x10^7= 20 000 000)

The value must be a number

Experimental Setup Details

13. Exposure Conditions (Open/Contained System)

Open: Petri dish, microplate 24-well plate, 96-well plate. Closed: centrifuge tube, eppendorf

- Open
- Contained
- Other

14. Sample Volume (ml)

The value must be a number

15. Sample Type

- Cell supernatant, virus isolate suspension (eg. VERO E6 supernatant)
- Biological sample (plasma)
- Biological sample (serum)
- Biological sample (saliva)
- Waste water
- Water
- Other

16. Suspension Medium, Buffer solution type

when irradiated with UV

- ◯ PBS (Phosphate-buffered saline)
- ◯ Virus Transport Medium (WHO), VTM
- ◯ DMEM (Dulbecco's Modified Eagle Medium)
- ◯ DMEM + 2% FBS
- ◯ DMEM + 10% FBS
- ◯ MEM (Minimum Essential Medium)
- ◯ EMEM (Eagle's Minimum Essential Medium)
- ◯ Other

17. Sample Temperature (°C)

When UV-irradiated

The value must be a number

UV-C Setup Details

18. UV-C Wavelength (nm)

Extract the wavelength of the UV-C light used, in nanometers.

The value must be a number

19. UV Light Source Type

- ◯ Low-Pressure Mercury Lamp
- ◯ Medium-Pressure Mercury Lamp
- ◯ Excimer Lamp (e.g., Krypton Chloride, KrCl)
- ◯ UV-C LED
- ◯ Pulsed Xenon Lamp
- ◯ Quartz Lamp
- ◯ Germicidal UV Lamp
- ◯ High-Intensity Discharge (HID) Lamp
- ◯ Cold Cathode UV Lamp
- ◯ Laser
- ◯ Other

20. UV Exposure Configuration

This question aims to identify the specific configuration of the UV lamp utilized in the study for UV-C inactivation of viruses. Please select the configuration that best describes the setup used.

- ◯ **Direct Exposure (Single Lamp):** A single UV lamp directly illuminating the target area or sample.
- ◯ **Enclosed UV Box:** An enclosed box or chamber specifically designed to house the UV lamp and target material.
- ◯ **Flow-Through Reactor:** A configuration where the target material flows through a reactor or tube exposed to UV light.
- ◯ **Multiple Lamps (Direct Exposure):** Multiple UV lamps providing direct exposure to the target area or sample.
- ◯ **Reflective Chamber:** A setup where the target area or sample is placed in a chamber with reflective surfaces to enhance UV exposure.
- ◯ **Rotating Platform:** A setup with a rotating platform to ensure uniform exposure to UV light.
- ◯ **UV Tunnel:** A tunnel-like structure where the target material passes through and is exposed to UV light.
- ◯ **Custom Configuration:** Any custom or specialized setup not covered by the above options (please specify).
- ◯ Other

21. Number of UV Lamps Used

The value must be a number

22. Lamp to Sample Distance (mm)

Record the distance between the UV lamp and the sample in millimeters.

The value must be a number

23. UV Light Source Performance (mW)

Extract the minimum performance of the UV Light Source in milliwatts.

The value must be a number

24. UV Light Intensity Minimum (W/cm^2)

Extract the minimum UV light intensity in watts per square centimeter. (can be calculated, if so note it!)

The value must be a number

25. UV Light Intensity Maximum (W/cm^2)

Extract the maximum UV light intensity in watts per square centimeter. (can be calculated, if so note it!)

The value must be a number

26. Exposure Duration Minimum (s)

The value must be a number

27. Exposure Duration Maximum (s)

The value must be a number

28. UV Dose Specification Minimum (mJ/cm²)

Extract the minimum UV dose specified in millijoules per square centimeter.

The value must be a number

29. Minimum Log rate of Virus Inactivation

Extract the minimum virus inactivation rate expressed in logarithmic reduction. (Can be calculated from percentages; Eg.: 3)

The value must be a number

30. Minimum % of Virus Inactivation

Extract the minimum virus inactivation rate expressed as a percentage. (Can be calculated from Log reductions; Eg.: 90)

The value must be a number

31. UV Dose Specification Maximum (mJ/cm²)

Extract the maximum UV dose specified in millijoules per square centimeter.

The value must be a number

32. Maximum Log rate of Virus Inactivation

Extract the maximum virus inactivation rate expressed in logarithmic reduction. (Can be calculated from percentages; Eg.: 5)

The value must be a number

33. Maximum % of Virus Inactivation

Extract the minimum virus inactivation rate expressed as a percentage. (Can be calculated from Log reductions; Eg.: 99.9)

The value must be a number

34. Stirring

Describe stirring or mixing methods used in UV-C inactivation setups as mentioned

- Magnetic Stirring
- Mechanical Agitation
- Recirculation Pump
- Air Bubbling
- Rotary Mixing
- Other

Reviewer Notes

35. Observed Proteomic or Nucleic Acid Damage Due to UV-C Exposure

This question seeks to identify the types of proteomic or nucleic acid damage observed as a result of UV-C exposure in the study. Please select all applicable types of damage documented.

36. Wordcount: Disinfection

Occurrence without references

The value must be a number

37. Wordcount: Inactivation

Occurrence without references

The value must be a number

38. Reviewer Notes

Study provides robust data on UV-C efficacy but lacks details on lamp maintenance.

39. Your opinion of this article? *

☆ ☆ ☆ ☆ ☆

40. In summary: include or exclude? *

This question seeks to determine whether to include or exclude specific studies or data points in the systematic review. Based on your analysis and the criteria set for the review, indicate if the summarized information should be included or excluded.

- ◯ Include
- ◯ Exclude

This content is neither created nor endorsed by Microsoft. The data you submit will be sent to the form owner.

Microsoft Forms
