# Supplementary material for: Wavelength-Specific UV-C Inactivation of Viruses in Liquids: Dose–Response, Mechanistic Insights, and Structural Integrity—A Systematic Review and Meta-Analysis
Source: Viruses. 2026 Feb 24;18(3):276. doi: 10.3390/v18030276 (PMC13030338; doi:10.3390/v18030276)
Supplement: Supplementary file 1 [file viruses-18-00276-s001.zip › 04_Resolved_Documentation_of_Meta-Analysis_Data_Extraction.pdf]

Decision on Article Figure Selection for Meta-Analysis

This document records the decision-making process regarding the selection of the most appropriate figure reading for each of the 18 scientific articles included in the current meta-analysis. The two available figure readings were provided by Hanna Dániel and Kopasz Zoltán.

Date: 2025-04-07

Location: University of Pécs, National Virology Laboratory (PTE-VNL)

Project Title: Spectral Efficacy: The Role of UV-C Light Frequency in Viral Inactivation and Disinfection

The evaluation and final decision on which figure to accept was carried out independently by two appointed seniors:

- Dr. Szabó-Meleg Edina

- Dr. Bányai Krisztián

After thorough review and mutual agreement, the accepted figure reading for each article is as follows:

Study ID (DOI)	Accepted Figure Reading (Author Name)
Study 1: 15. Efficiency improvement of AlGaN-based deep-ultraviolet light-emitting diodes and their virus inactivation application; 10.35848/1347-4065/ac10f2	Kopasz Zoltán
Study 2: 2.Comparison of the inactivation capacity of various UV wavelengths on SARS-CoV-2;	Kopasz Zoltán

10.1016/j.bbrep.2022.101379	
Study 3: 24. Immunogenic properties of SARS-CoV-2 inactivated by ultraviolet light;<br>10.1007/s00705-022-05530-7	Not Relevant (original authors provided a table containing the relevant information)
Study 4: 27. Inactivation of HCoV-NL63 and SARS-CoV-2 in aqueous solution by 254 nm UV-C;<br>10.1016/j.jphotobiol.2023.112757	Hanna Dániel
Study 5: 30. Inactivation of Material from SARS-CoV-2-Infected Primary Airway Epithelial Cell Cultures;<br>10.3390/mps4010007	Identical Reading
Study 6: 32. Inactivation of SARS-CoV-2 and COVID-19 Patient Samples for Contemporary Immunology and Metabolomics Studies; 10.4049/immunohorizons.2200005	Identical Reading
Study 7: 10. 34. Inactivation of SARS-CoV-2 isolates from lineages B.1.1.7 (Alpha), P.1 (Gamma) and B.1.110 by heating and UV irradiation; 10.1016/j.jviromet.2021.114216	Identical Reading
Study 8: 41. Methods of Inactivation of SARS-CoV-2 for Downstream Biological Assays; 10.1093/infdis/jiaa507	Identical Reading
Study 9: 44. Optimized parameters for effective SARS-CoV-2 inactivation using UVC-LED at 275Â nm; 10.1038/s41598-022-20813-4	Not Relevant (original authors provided a table containing the relevant information)
Study 10: 5. Determination of the UV	EXCLUDED DUE TO RISK OF BIAS (It

Inactivation Constant under 280 nm UV LED Irradiation for SARS-CoV-2; 10.1111/php.13653	is unclear whether base-3 logarithmic transformation was used in the formula F = N/N_0, or whether the values reflect actual PFU/ml. The data presented in Figures 1 and 2 do not correspond to the data described in the text. At 24 minutes, the reported irradiance is 23 J/m², which contradicts the values shown in the figures. Overall, the methodology and results are ambiguous and lack clarity.)
Study 11: 51. SARS-CoV-2 Production, Purification Methods and UV Inactivation for Proteomics and Structural Studies; 10.3390/v14091989	Kopasz Zoltán
Study 12: 53. Stability of SARS-CoV-2 and other airborne viruses under different stress conditions; 10.1007/s00705-021-05293-7	Hanna Dániel
Study 13: 54. Structural and Immunoreactivity Properties of the SARS-CoV-2 Spike Protein upon the Development of an Inactivated Vaccine; 10.3390/v15020480	Hanna Dániel
Study 14: 58. Systematic evaluating and modeling of SARS-CoV-2 UVC disinfection; 10.1038/s41598-022-09930-2	Identical Reading
Study 15: 6. Disinfection of SARS-CoV-2 by UV-LED 267Â nm: comparing different variants; 10.1038/s41598-023-35247-9	Identical Reading

Study 16: 61. UV 254 nm is more efficient than UV 222 nm in inactivating SARS-CoV-2 present in human saliva; 10.1016/j.pdpdt.2022.103015	Not Relevant (original authors provided a table containing the relevant information)
Study 17: 67. UVC disinfects SARS-CoV-2 by induction of viral genome damage +out apparent effects on viral morphology and proteins; 10.1038/s41598-021-93231-7	Identical Reading
Study 18: 72. Wavelength dependence of ultraviolet light inactivation for SARS-CoV-2 omicron variants; 10.1038/s41598-023-36610-6	Hanna Dániel
Study 19: 9. Effect of inactivation methods on sars-cov-2 virion protein and structure; 10.3390/v13040562	Not Relevant (original authors provided a table containing the relevant information)

Both seniors confirm that the selections were made jointly, based on clarity, accuracy, and relevance to the meta-analytical outcomes.

Signatures:

Edina Szabó-Meleg
Digitally signed by Edina Szabó-Meleg
Date: 2025.05.22 18:05:19+02'00'

Dr. Szabó-Meleg Edina

Krisztián Bányai
Digitally signed by Krisztián Bányai
Date: 2025.05.22 18:02:48+02'00'

Dr. Bányai Krisztián
