# Supplementary material for: Wavelength-Specific UV-C Inactivation of Viruses in Liquids: Dose–Response, Mechanistic Insights, and Structural Integrity—A Systematic Review and Meta-Analysis
Source: Viruses. 2026 Feb 24;18(3):276. doi: 10.3390/v18030276 (PMC13030338; doi:10.3390/v18030276)
Supplement: Supplementary file 1 [file viruses-18-00276-s001.zip › 05_Cross_Thematic_Qualitative_Analysis_PowerBI.pdf]

Count of Title by UV-C Wavelength (nm)

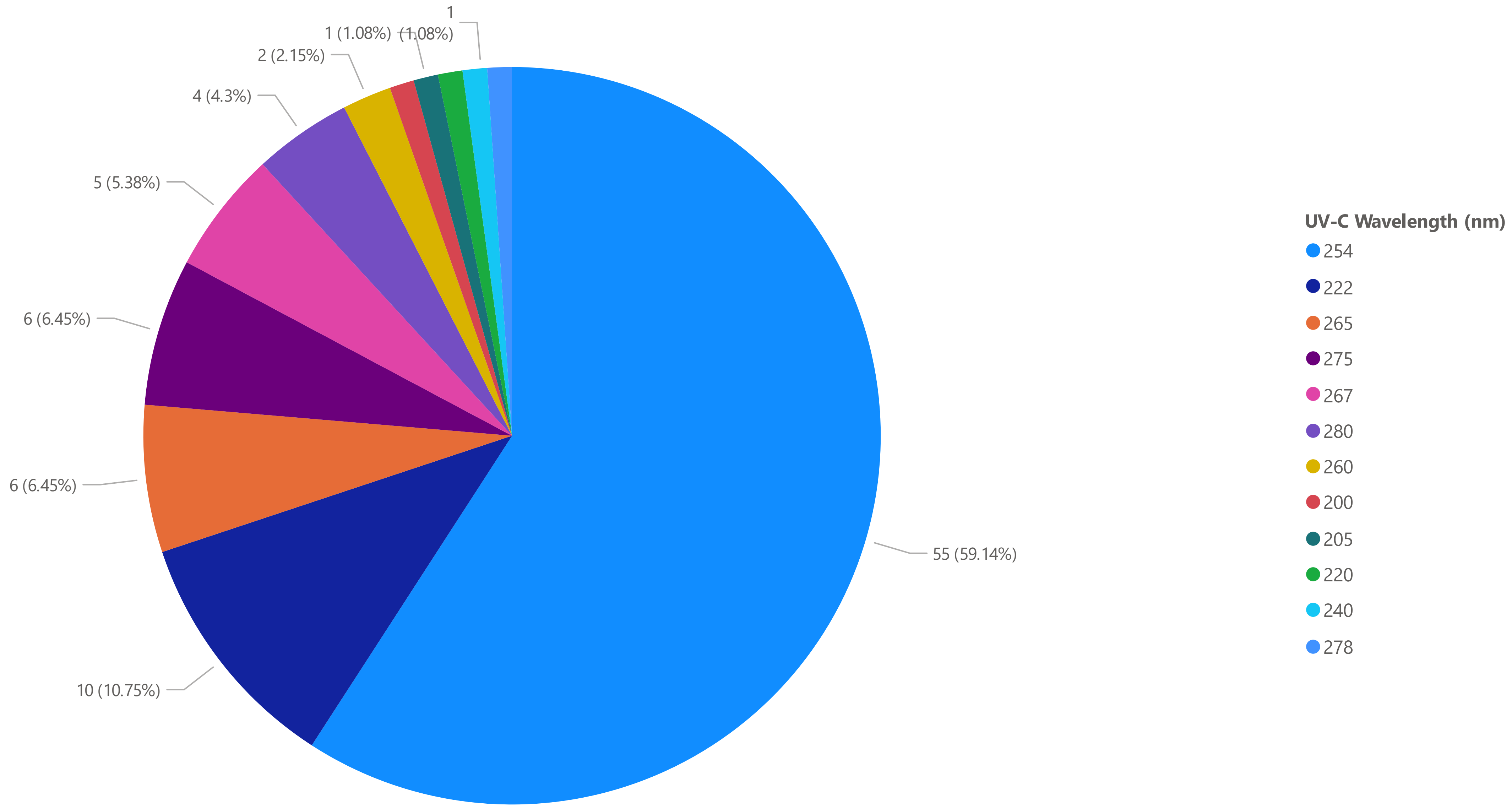

Virus Name	2019	2020	2021	2022	2023	Total
Yellow fever virus	1					**1**
Vesicular stomatitis virus				1		**1**
Tulane Virus		1				**1**
Swine vesicular disease virus	1					**1**
Swine influenza virus	1					**1**
Sindbis virus				1		**1**
Senecavirus A	1					**1**
SARS-CoV-2		1	5	9	4	**19**
SARS-CoV		1				**1**
Rotavirus		1				**1**
Pseudorabies virus	1			1		**2**
Porcine reproductive and respiratory syndrome virus	1					**1**
Porcine parvovirus	1			1		**2**
Porcine epidemic diarrhea virus	1					**1**
Porcine circovirus 2	1					**1**
PhiX174					1	**1**
Phi6					1	**1**
Nipah virus		1				**1**
Mumps virus				1		**1**
MS2	1		1		2	**4**
human rhinovirus				1		**1**
Human respiratory syncytial virus				1		**1**
Human norovirus					1	**1**
Human Coronavirus					1	**1**
Human adenovirus 2		1				**1**
Hepatitis A virus				1		**1**
Feline calicivirus	1			1		**2**
Escherichia phage phiX174					1	**1**
Escherichia coli bacteriophage Q-β	1					**1**
Encephalomyocarditis virus				1		**1**
Crimean-Congo hemorrhagic fever virus		1				**1**
Coxsackievirus				1		**1**
Classical swine fever virus	1					**1**
Bovine viral diarrhea virus	1			1		**2**
African swine fever virus			1			**1**
**Total**	**3**	**4**	**7**	**10**	**9**	**33**

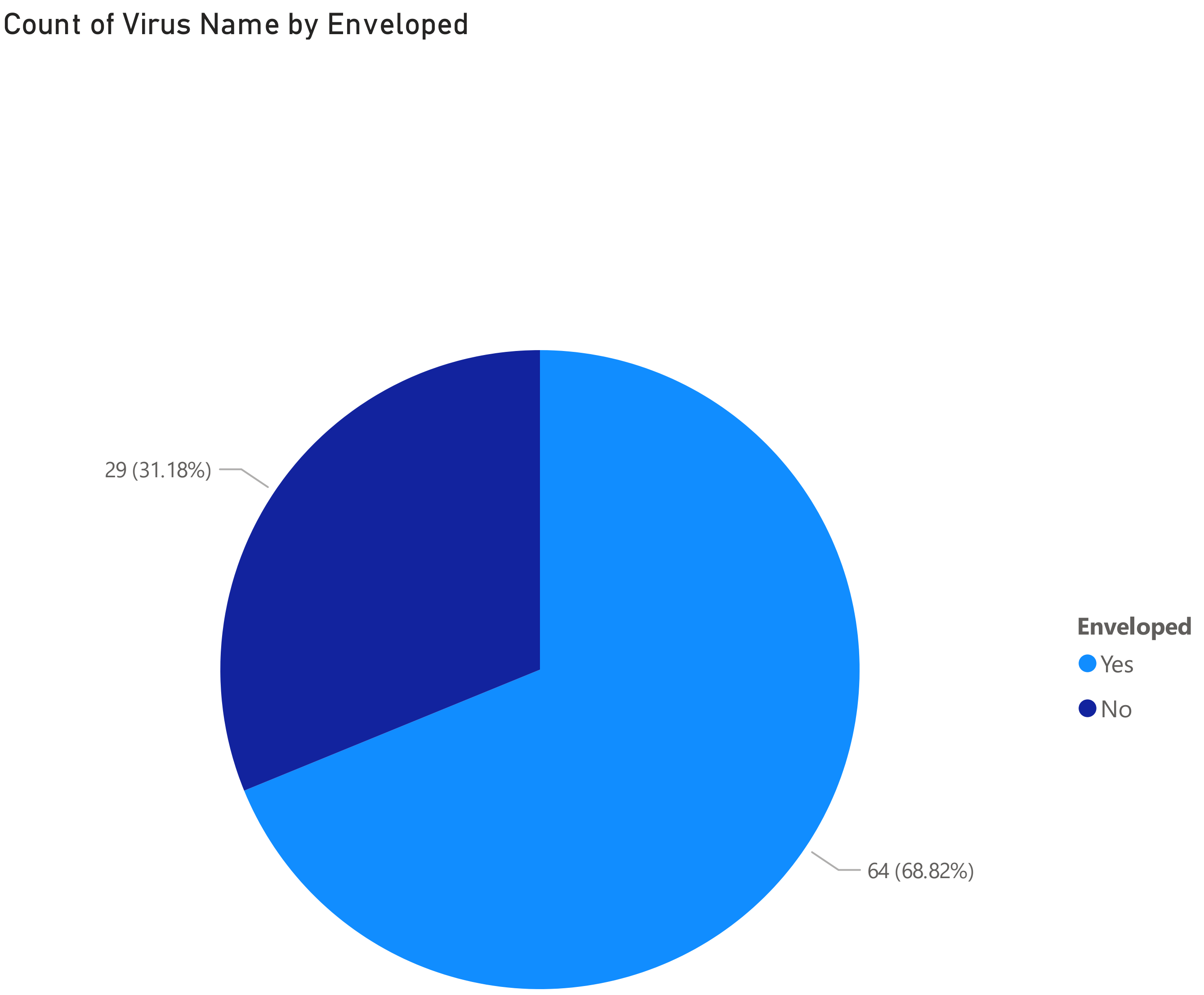

Count of Exposure Conditions (Open/Contained System) by Virus Name and UV Light Source Type

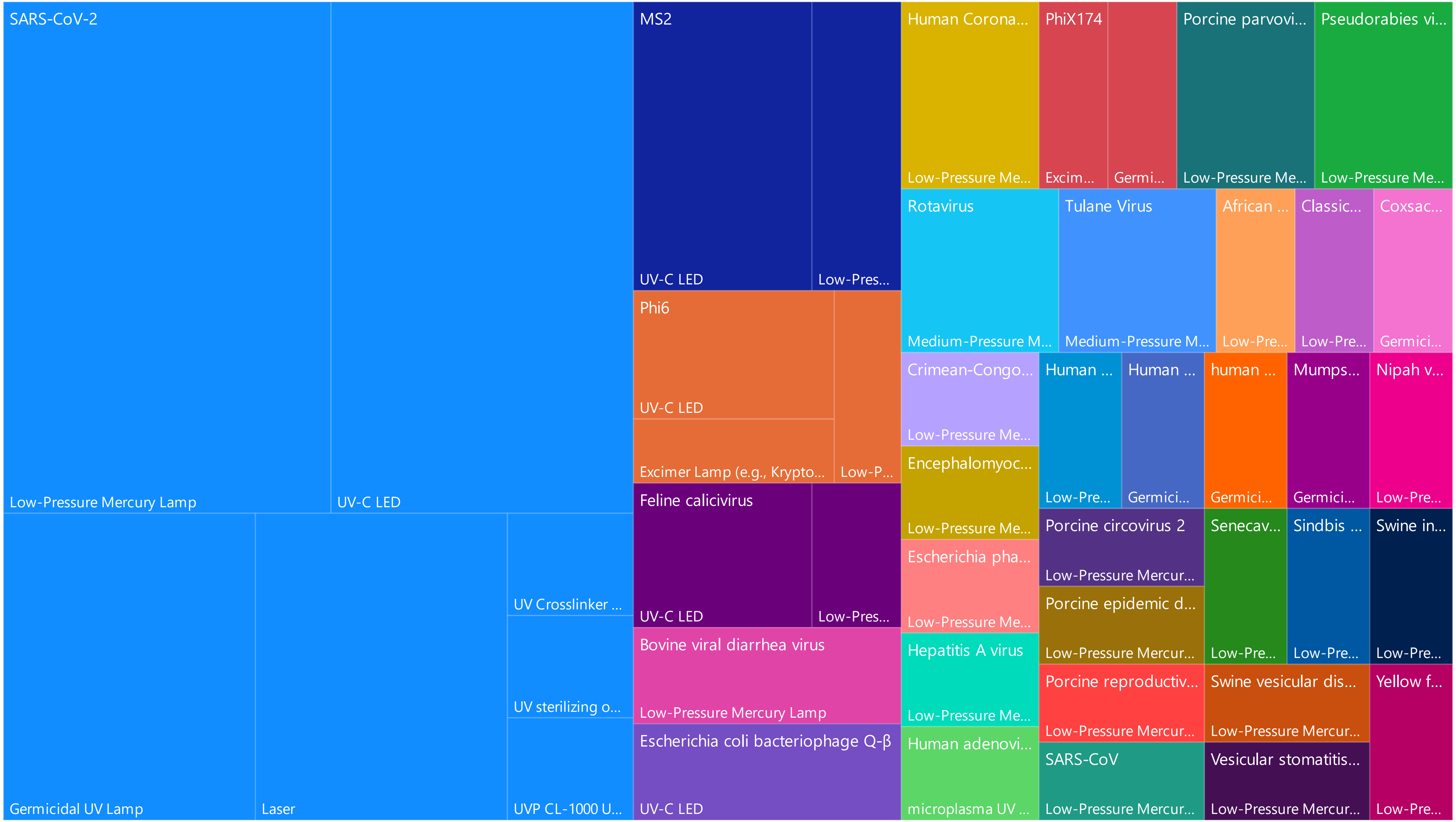

Count of Virus Name by Suspension Medium, Buffer solution type

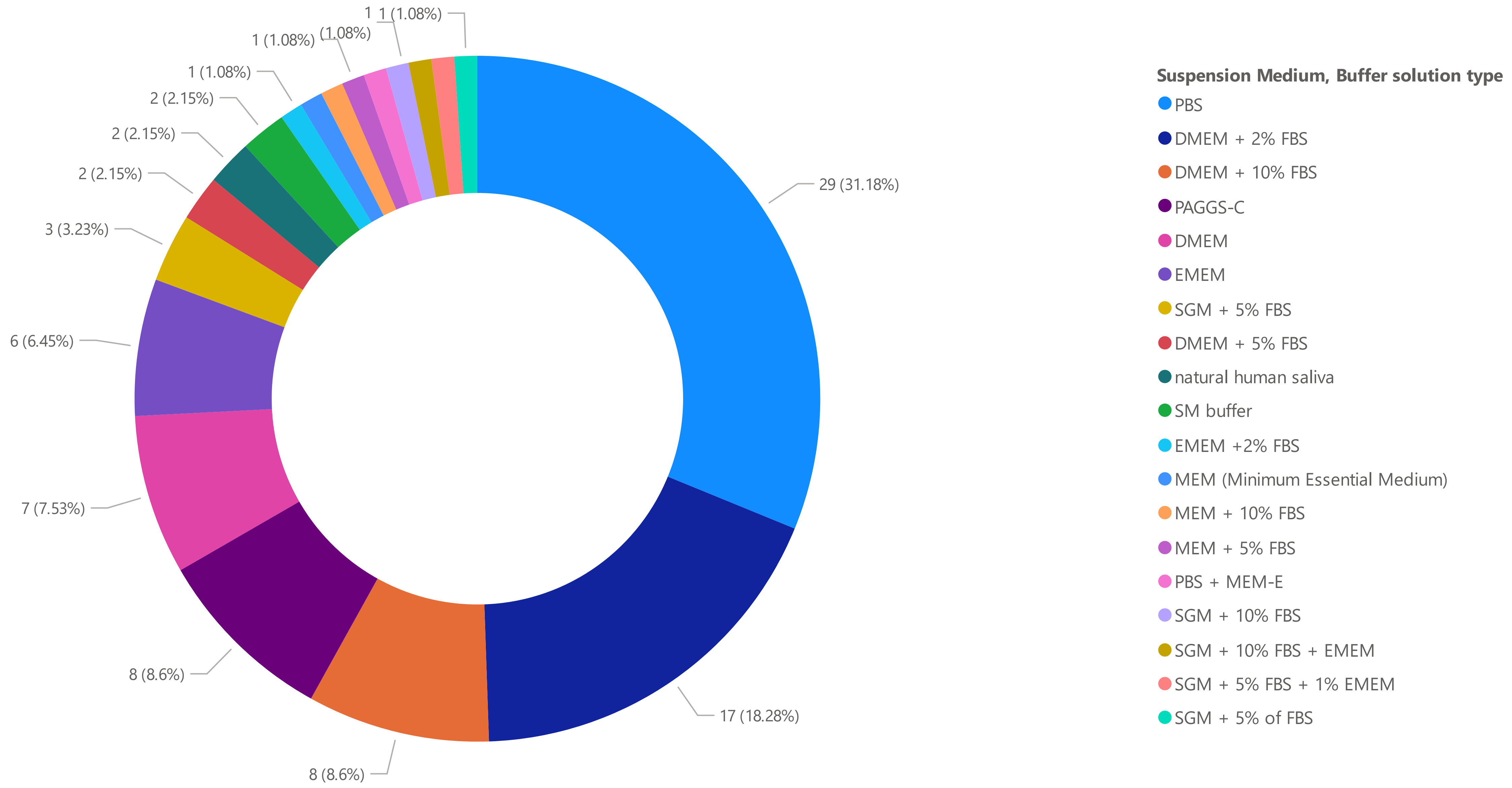

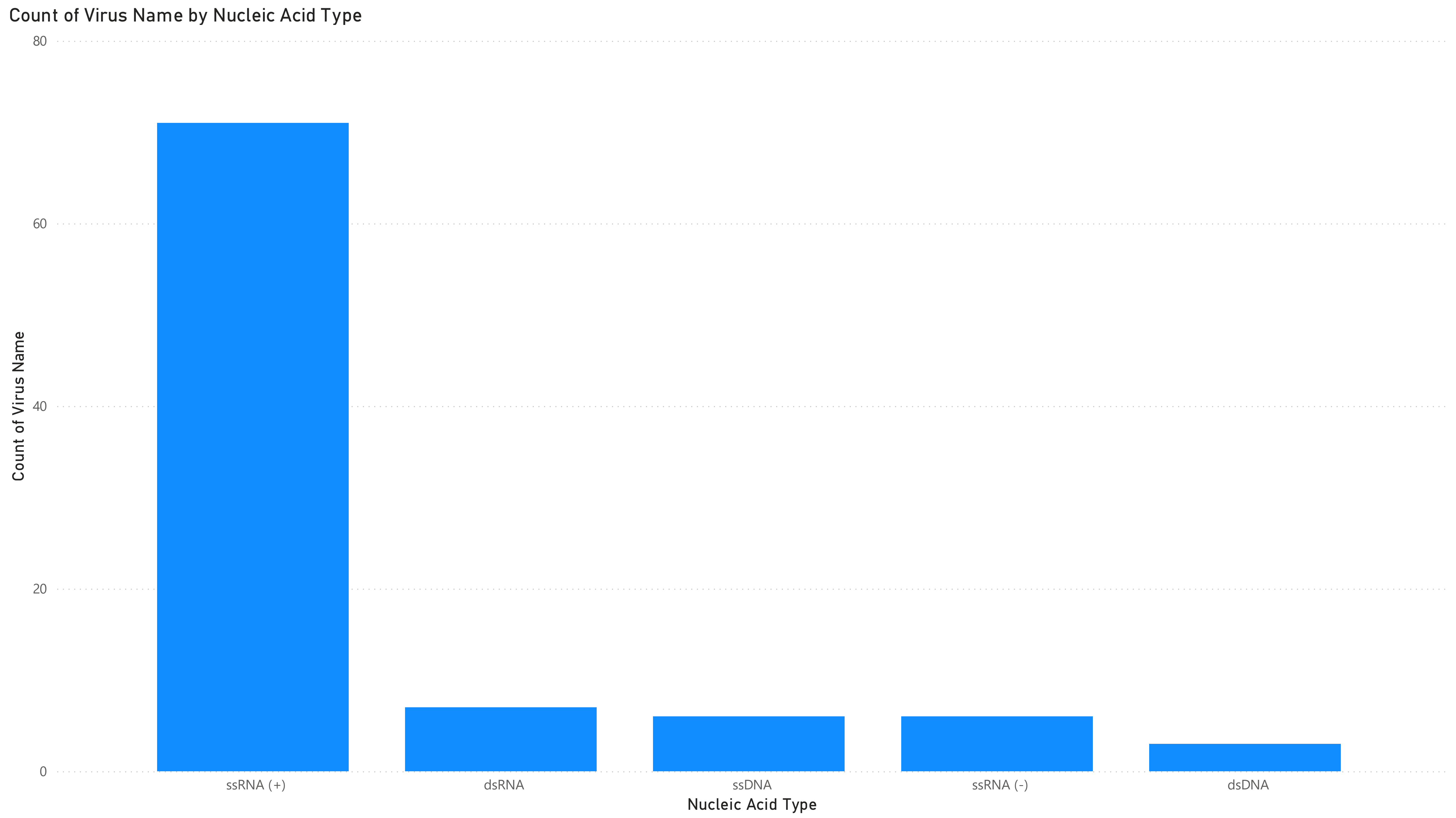
Count of Virus Name by Nucleic Acid Type
80
60
40
20
0
Count of Virus Name
ssRNA (+)
dsRNA
ssDNA
ssRNA (-)
dsDNA
Nucleic Acid Type

Genome size (bp, nt)	Virus Name
10730	Yellow fever virus
11161	Vesicular stomatitis virus
11699	Sindbis virus
12300	Classical swine fever virus
12570	Bovine viral diarrhea virus
12573	Bovine viral diarrhea virus
13150	Swine influenza virus
13385	Phi6
143460	Pseudorabies virus
15226	Human respiratory syncytial virus
15384	Mumps virus
15430	Porcine reproductive and respiratory syndrome virus
170101	African swine fever virus
1770	Porcine circovirus 2
18000	Rotavirus
18246	Nipah virus
19207	Crimean-Congo hemorrhagic fever virus
27553	Human Coronavirus
28030	Porcine epidemic diarrhea virus
2907	Human adenovirus 2
29714	SARS-CoV-2
29721	SARS-CoV-2
29727	SARS-CoV
29785	SARS-CoV-2
29800	SARS-CoV-2
29806	SARS-CoV-2
29809	SARS-CoV-2
29848	SARS-CoV-2
29850	SARS-CoV-2
29854	SARS-CoV-2
29855	SARS-CoV-2
29867	SARS-CoV-2
29870	SARS-CoV-2
29871	SARS-CoV-2

Observed Proteomic or Nucleic Acid Damage Due to UV-C Exposure

We observed a linear correlation between the log10 reduction in the copy number of the VP7 gene of RV and 220-nm UV doses...suggesting that 220-nm irradiation caused damage to both the TV and RV genomes. "Treatment of viruses + 220-nm UV may compromise the structure of the capsid, such that some or all of the small segments of the RV genome escape from capsids that are no longer intact." The document also notes that "220-nm UV targets proteins/amino acid residues," which suggests that the viral capsid proteins of RV might also be damaged, affecting the virus's ability to interact + host cells.

UV-C exposure primarily results in nucleic acid damage. Specifically, the UV-C light used in the study damages viral nucleic acids, effectively reducing the infectivity of the viruses being studied (SARS-CoV, CCHFV, and NiV). This damage to the nucleic acids is a key mechanism by which the UV-C light inactivates the viruses

Time relation to constant dose noted

This hypothesis, if correct, would suggest small changes in the virus genome sequence could result in dramatic changes in its UV resistance, and should be considered when looking for the use of UV irradiation as way to combat pathogenic viruses.

These alterations indicate that UV-C exposure can cause oxidative damage to certain biomolecules, which could potentially impact downstream metabolomic analyses

The study specifically mentions RNA damage as observed through quantitative reverse transcription polymerase chain reaction (RT-qPCR) analysis. The reduction in RNA amplification after UV-C exposure indicates that the UV light caused damage to the viral RNA. The study implies that UV-C exposure likely causes damage to the protein layer that shields the RNA, particularly below 240 nm, where proteins strongly absorb UV light, potentially leading to protein modifications or damage.

The study reports that UV254 exposure caused significant damage to the phiX174 genome, particularly in the L and S segments of the viral DNA. The main mechanism of genome damage by UV254 is the formation of photoproducts like cyclobutane pyrimidine dimers (CPDs) and pyrimidine (6-4) pyrimidone adducts (6-4 PP). UV254 exposure also caused some damage to viral proteins, although the extent was less severe compared to genome damage. Proteins such as B, H, and D were affected, + the study indicating that UV254 "had a little effect on viral proteins," and mentioning, "UV254 might inhibit physiological processes involving H protein, such as the injection of viral genomes."

The study reported significant damage to the viral genome, including strand breaks and potential pyrimidine dimer formation. The study specifically noted that UVC exposure did not result in observable damage to the viral proteins, including the spike (S) and nucleocapsid (N) proteins.

The study notes that UV254 irradiation primarily causes damage to nucleic acids by inducing pyrimidine dimers. Specifically, pyrimidine bases (cytosine and thymine) are more photoreactive than purine bases, leading to the formation of both pyrimidine hydrates and pyrimidine dimers, which are critical to the inactivation process. Quote: "Specifically, pyrimidine bases are more photoreactive than purine bases, forming both pyrimidine hydrates and pyrimidine dimers" RNA Degradation: The study also discusses the degradation of the RNA genome as a significant effect of UV254 exposure. This degradation is quantified by the decay rate constants for various regions of the HuNoV genome, indicating the extent of nucleic acid damage across different portions of the viral RNA.

The study noted that UV-C exposure at 265 nm caused significant damage to the viral RNA, particularly in the case of MS2 bacteriophage.

The linear scale stresses the difference in significance of the two measurement techniques. qRT-PCR (blue stars) measures the total amount of viral RNA recovered +out distinguishing between infectious viruses and nucleic acids derived from inactivated viruses and thus provides a higher 2 Biffi Silvia et al. count, especially at low doses, i.e. low exposure times.

Text data on max inactiovation and dose

Solutions of AdV were exposed to microplasma UV irradiation, and both the inactivation efficiency and mechanism were evalu- ated by the set of molecular assays, as shown in Fig. 5. The exper- iment was repeated + a different UV irradiance (0.10 mW/cm2) at the center of the solution + 4 cm distance from the lamp, and the results showed no significant difference (p>0.05) (Fig. S5). This result confirmed that the microplasma UV irradiation kinetics could be normalized by the product I × t referred to as fluence in this study Eqs. (7)–((9)). The AdV inactivation rate constant, which was shown in the slope of the infectivity loss over the fluence, was 0.142 cm2 /mJ. Among the three viral components, the capsid dam- age explained the 3-log10 infectivity loss (p>0.05), while genome and fiber damages were significantly different from infectivity loss (p<0.05). The molecular assays indicated that microplasma UV ir- radiation mostly attacked the capsid proteins to inactivate AdV.This observation can be explained by the overlapping of the UV ab- sorbance of capsid proteins + the microplasma UV wavelength. These results agree + a previous study using a sodium dodecyl sulfate polyacrylamide gel electrophoresis assay to report that the monochromatic UV damaged about 40% of the hexon and penton proteins at 220 nm of 22.7 mJ/cm2 (Beck et al., 2018). Genome damage increased up to about 1 log of reduction as fluence increased until about 5 mJ/cm2 before reaching saturation. This saturation was also confirmed + the naked DNA sample (Fig. S6). This plateau was attributed to the reversibility of the le- sion of cyclobutane pyrimidine dimers (CPDs), the most common lesion caused by UVC irradiation. The CPDs formation follows a pseudo-zero-order reaction. In contrast, the reversal reaction con- forms to a first-order reaction, so the two reactions reach an equi- librium state at a certain concentration of CPDs in the AdV genome (Errol et al., 2006). The fiber damage was not significantly different from the neg- ative control (p>0.05) at fluences of less than 21.2 mJ/cm2 . This observation meant monochromatic UV at 222 nm is not effective to degrade the viral fiber proteins. A similar trend was also con- firmed by Beck et al. (2018), showing fiber damage was negligible at fluences less than 22.7 mJ/cm2 of monochromatic UV irradiation at 220 nm generated by a deuterium UV lamp + a bandpass fil- ter. The results of the molecular assays also provided knowledge on the damage patterns on AdV. These

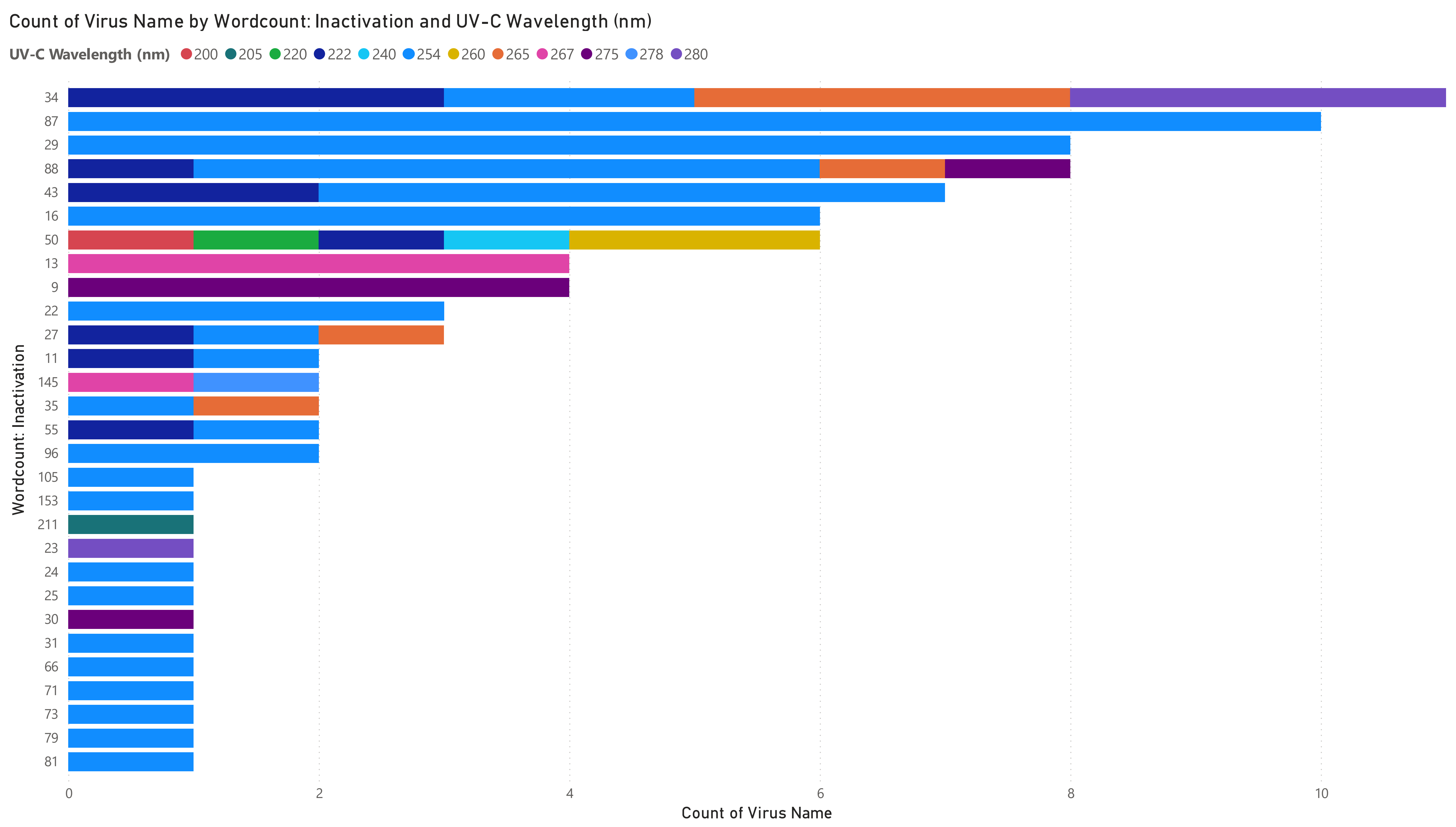

Count of Virus Name by Wordcount: Inactivation and UV-C Wavelength (nm)
UV-C Wavelength (nm)
200
205
220
222
240
254
260
265
267
275
278
280
Wordcount: Inactivation
34
87
29
88
43
16
50
13
9
22
27
11
145
35
55
96
105
153
211
23
24
25
30
31
66
71
73
79
81
0
2
4
6
8
10
Count of Virus Name

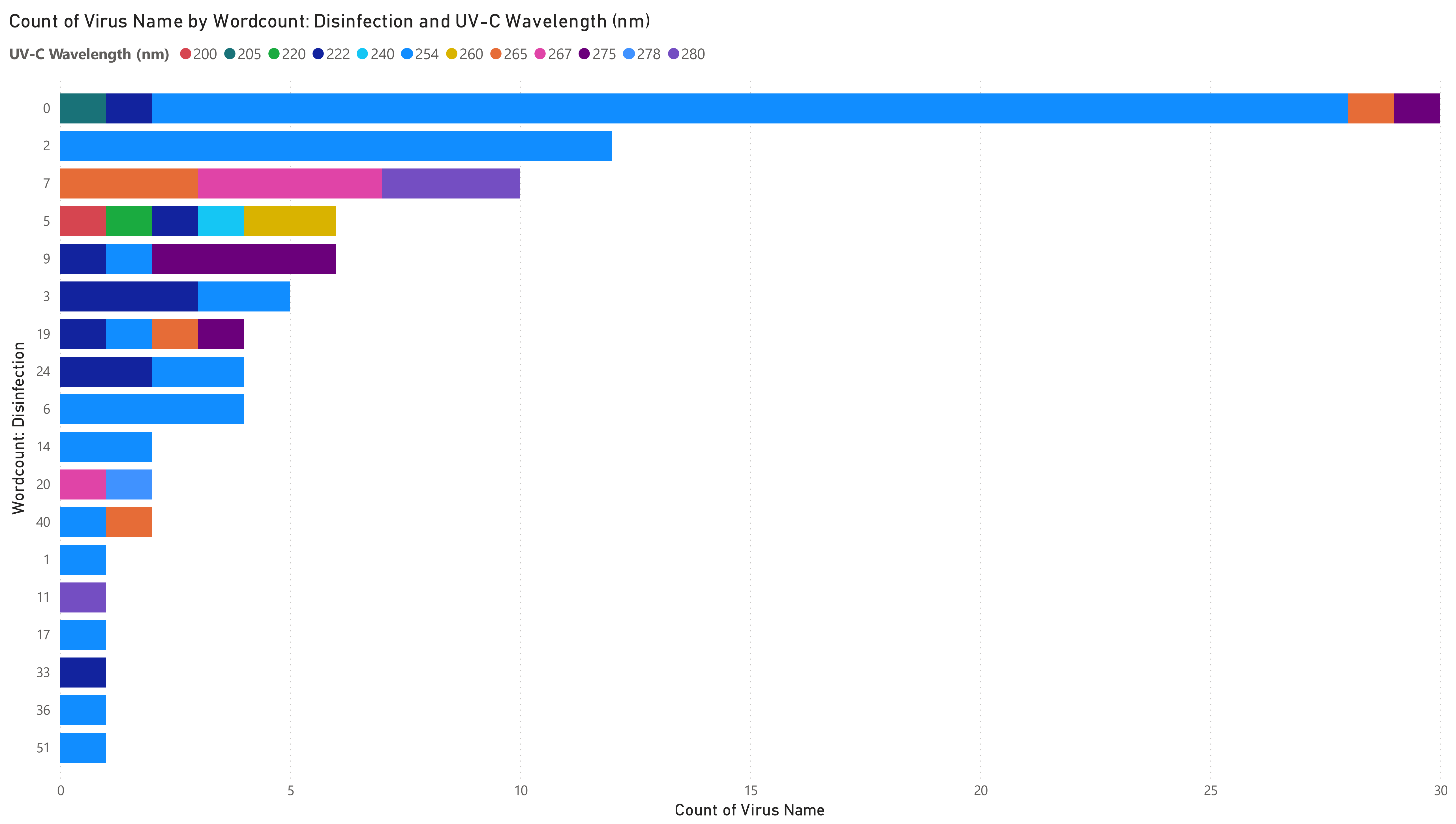

Count of Virus Name by Wordcount: Disinfection and UV-C Wavelength (nm)
UV-C Wavelength (nm)
200
205
220
222
240
254
260
265
267
275
278
280
Wordcount: Disinfection
0
2
7
5
9
3
19
24
6
14
20
40
1
11
17
33
36
51
0
5
10
15
20
25
30
Count of Virus Name

Count of Title by UV-C Wavelength (nm)

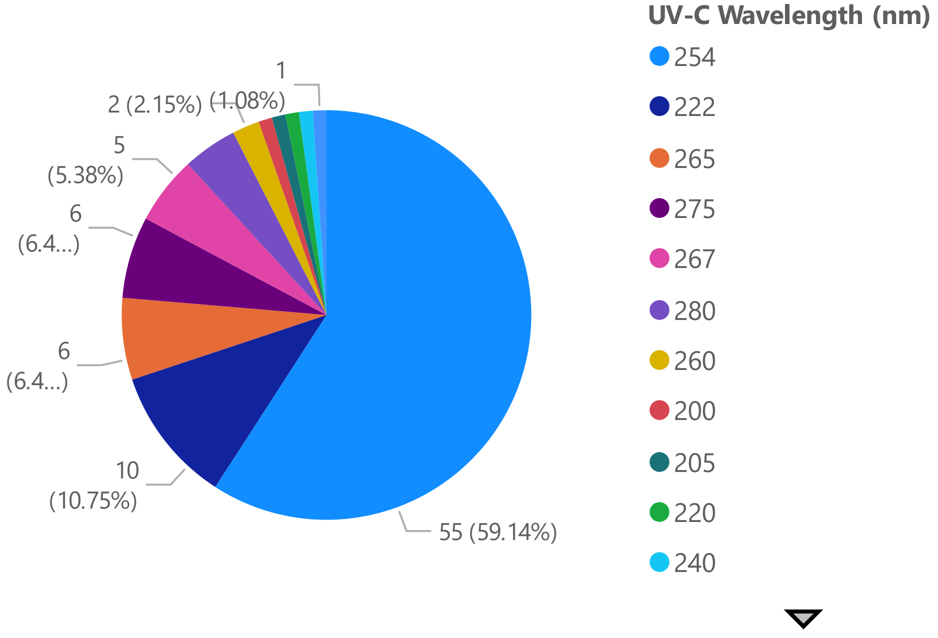

Count of Exposure Conditions (Open/Contained System) by Virus Name and UV Light Source Type

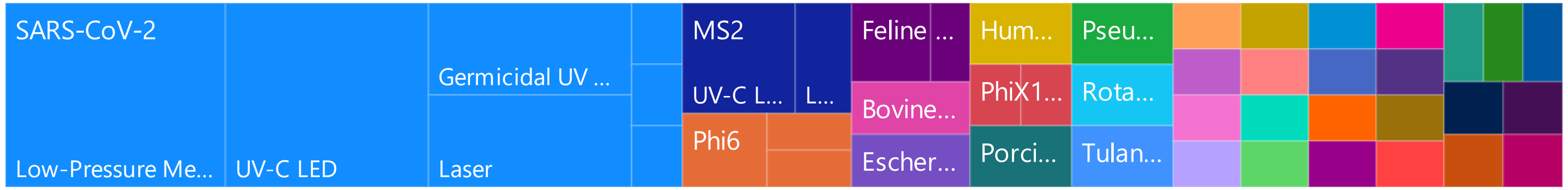

Count of Virus Name by Suspension Medium, Buffer solution type

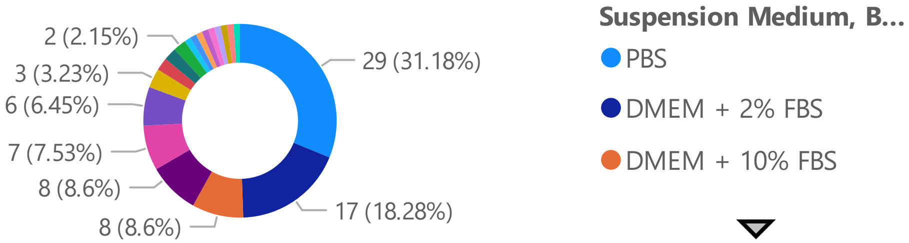

Count of Virus Name by Enveloped

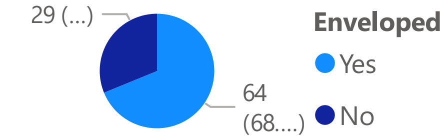

Count of Virus Name by Wordcount: Inactivation and UV-C Wavelength (nm)

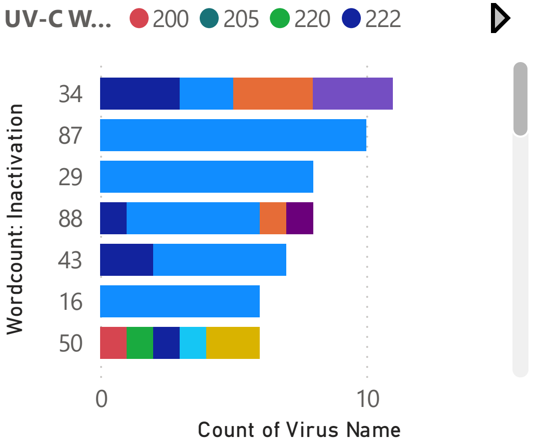

Count of Virus Name by Wordcount: Disinfection and UV-C Wavelength (nm)

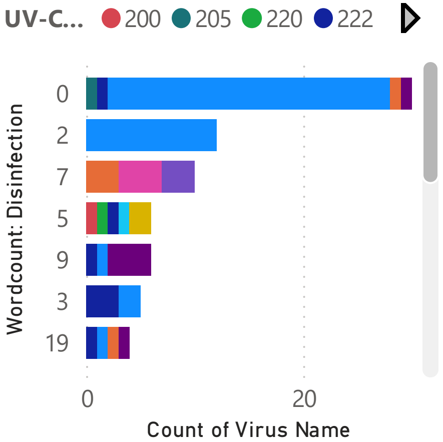

Count of Virus Name by Nucleic Acid Type

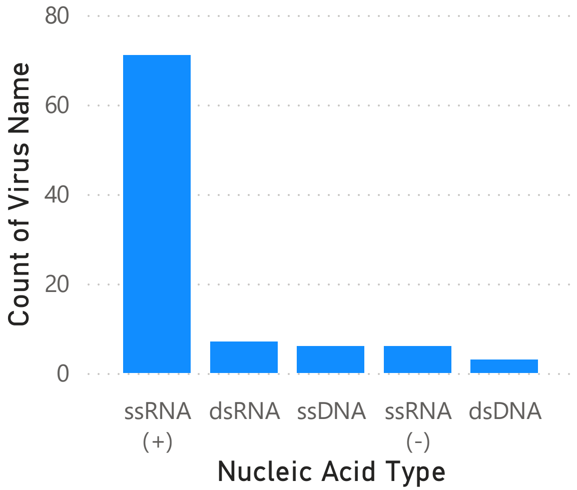

Genome size (bp, nt)	Virus Name
10730	Yellow fever virus
11161	Vesicular stomatitis virus
11699	Sindbis virus
12300	Classical swine fever virus
12570	Bovine viral diarrhea virus
12573	Bovine viral diarrhea virus
13150	Swine influenza virus
13385	Phi6
[illegible]	[illegible]

Virus Name	2019	2020	2021	2022	2023	Total
Yellow fever virus	1					1
Vesicular stomatitis virus				1		1
Tulane Virus		1				1
Swine vesicular disease virus	1					1
Swine influenza virus	1					1
**Total**	**3**	**4**	**7**	**10**	**9**	**33**

Observed Proteomic or Nucleic Acid Damage Due to UV-C Exposure
We observed a linear correlation between the log10 reduction in the copy number of the VP7 gene of RV and 220-nm UV doses...suggesting that 220-nm irradiation caused damage to both the TV and RV genomes. "Treatment of viruses + 220-nm UV may compromise the structure of the capsid, such that some or all of the small segments of the RV genome escape from capsids that are no longer intact." The document also notes that "220-nm UV targets proteins/amino acid residues," which suggests that the viral capsid proteins of RV might also be damaged, affecting the virus's ability to interact + host cells.
[illegible]
