# Supplementary material for: Wavelength-Specific UV-C Inactivation of Viruses in Liquids: Dose–Response, Mechanistic Insights, and Structural Integrity—A Systematic Review and Meta-Analysis
Source: Viruses. 2026 Feb 24;18(3):276. doi: 10.3390/v18030276 (PMC13030338; doi:10.3390/v18030276)
Supplement: Supplementary file 1 [file viruses-18-00276-s001.zip › 06_Included_studies_complete_inactivation_qualitative_analysis_definition.pdf]

**Study**	**Complete Inactivation Definition (extracted by usage from included studies)**
(Li et al., 2023)	NA
(Masaike et al., 2019)	NA
(Barrow et al., 2021)	“Fully inactivated" refers to the absence of cytopathic effect in Vero cells across all dilutions after UV-C exposure.
(Yu et al., 2023)	The phrase “complete inactivation” is not explicitly used in this study. Instead, the study consistently uses "4-log_{10} inactivation" as the maximum level achieved, and equates inactivation to genome damage and functional loss, especially the inability to inject the viral genome.
(Eddins et al., 2022)	Thus, complete inactivation was defined by absence of detectable virus via plaque assay after UV-C treatment.
(Ulloa et al., 2021)	This quote confirms that complete inactivation was defined as reaching the assay’s detection limit
(Blazquez et al., 2021)	Virus was undetectable even after 3 blind passages indicating complete inactivation."
(Saito et al., 2021)	Complete inactivation was inferred by 5-log (99.999%) reduction.
(Nyangaresi et al., 2023)	Either by no colonies observed or no repair after inactivation” at specified fluences.
(Blazquez et al., 2019)	Detection was based on $TCID_{50}$ quantification by microtiter assay, followed by three blind passages in cell culture to verify complete inactivation.
(Matsuura et al., 2022)	NA

(Gracheva et al., 2022)	Complete inactivation was defined by no detectable infectivity even after three blind passages
(Eickmann et al., 2020)	Complete inactivation" is operationally defined by the absence of detectable infectivity in vitro using $TCID_{50}$ assays after treatment.
(Faddy et al., 2019)	below the detection limit of the plaque assays used, "complete inactivation" corresponds to no detectable infectious virus remaining post-treatment
(Oh et al., 2020)	In this study, "complete inactivation" is interpreted as a 4-$\log_{10}$ (99.99%) reduction in viral infectivity
(Patterson et al., 2020)	In this study, "complete inactivation" of SARS-CoV-2 is defined as the absence of cytopathic effect (CPE) and undetectable viable virus in cell culture following treatment, confirmed by multiple passages in Vero E6 cells with $TCID_{50}$ assays.
(Handke et al., 2022)	Thus, "complete inactivation" was operationalized as undetectable pathogen presence in treated RBCs within assay limits.
(Lee et al., 2022)	"complete inactivation" here relies on reaching or exceeding a 4-log reduction under clearly defined dose-response parameters.
(Plavec et al., 2022)	"complete inactivation" here is operationally defined by the total loss of detectable PFU post-treatment under standard assay sensitivity.
(Fumagalli et al., 2022)	Thus, complete inactivation here corresponds to undetectable infectivity post-

	treatment, with specific quantified UV exposure thresholds
(Kordyukova et al., 2023)	absence of detectable infectious virus after treatment, confirmed by no cytopathic effect (CPE) and a $TCID_{50}$ of 1×10^0 in Vero cells following inactivation protocols including β-propiolactone, formaldehyde, and UV light . For rigorous confirmation, samples were also passaged again in cell culture with no CPE observed, ruling out residual infectivity.
(Freeman et al., 2022)	4-log_{10} reduction in viral infectivity, confirmed by absence of detectable plaques in Vero E6 cell assays
(Atari et al., 2023)	defined as a ≥5-log_{10} reduction in viral copy number (i.e., >99.999% reduction), determined via $TCID_{50}$-based RT-qPCR infectivity assays
(Rockey et al., 2020)	“complete inactivation” of human norovirus (HuNoV) is defined by a 4-log_{10} reduction (99.99%) in genome integrity, extrapolated from RT-qPCR measurements covering ~50% of the viral genome
(Araud et al., 2020)	“complete inactivation” is defined operationally by this 4-log reduction threshold in combination with loss of functional infectivity.
(Weyersberg et al., 2023)	“complete inactivation” here is explicitly a ≥4-log reduction in viable virus compared to controls
(Lo et al., 2021)	4.9-log_{10} reduction in $TCID_{50}$, with no detectable viral replication thereafter
(Baldasso et al., 2021)	“complete inactivation” is defined operationally by meeting or exceeding a log-

	reduction value (LRV) of ≥3.5, which aligns with the World Health Organization's threshold for protective disinfection of drinking water
(Sun et al., 2023)	NA
(Fujimoto et al., 2023)	NA
(Loveday et al., 2021)	the total absence of detectable plaques in a standard plaque assay

References:

Araud, E., Fuzawa, M., Shisler, J. L., Li, J., & Nguyen, T. H. (2020). UV Inactivation of Rotavirus and Tulane Virus Targets Different Components of the Virions. *Appl Environ Microbiol*, *86*(4). https://doi.org/10.1128/AEM.02436-19

Atari, N., Mamane, H., Silberbush, A., Zuckerman, N., Mandelboim, M., & Gerchman, Y. (2023). Disinfection of SARS-CoV-2 by UV-LED 267 nm: comparing different variants. *Sci Rep*, *13*(1), 8229. https://doi.org/10.1038/s41598-023-35247-9

Baldasso, V., Lubarsky, H., Pichel, N., Turolla, A., Antonelli, M., Hincapie, M., Botero, L., Reygadas, F., Galdos-Balzategui, A., Byrne, J. A., & Fernandez-Ibanez, P. (2021). UVC inactivation of MS2-phage in drinking water - Modelling and field testing. *Water Res*, *203*, 117496. https://doi.org/10.1016/j.watres.2021.117496

Barrow, K. A., Rich, L. M., Vanderwall, E. R., Reeves, S. R., Rathe, J. A., White, M. P., & Debley, J. S. (2021). Inactivation of Material from SARS-CoV-2-Infected Primary Airway Epithelial Cell Cultures. *Methods Protoc*, *4*(1). https://doi.org/10.3390/mps4010007

Blazquez, E., Rodriguez, C., Rodenas, J., Navarro, N., Riquelme, C., Rosell, R., Campbell, J., Crenshaw, J., Segales, J., Pujols, J., & Polo, J. (2019). Evaluation of the effectiveness of the SurePure Turbulator ultraviolet-C irradiation equipment on inactivation of different enveloped and non-enveloped viruses inoculated in commercially collected

liquid animal plasma. *PLoS One*, *14*(2), e0212332. https://doi.org/10.1371/journal.pone.0212332

Blazquez, E., Rodriguez, C., Rodenas, J., Rosell, R., Segales, J., Pujols, J., & Polo, J. (2021). Effect of spray-drying and ultraviolet C radiation as biosafety steps for CSFV and ASFV inactivation in porcine plasma. *PLoS One*, *16*(4), e0249935. https://doi.org/10.1371/journal.pone.0249935

Eddins, D. J., Bassit, L. C., Chandler, J. D., Haddad, N. S., Musall, K. L., Yang, J., Kosters, A., Dobosh, B. S., Hernandez, M. R., Ramonell, R. P., Tirouvanziam, R. M., Lee, F. E., Zandi, K., Schinazi, R. F., & Ghosn, E. E. B. (2022). Inactivation of SARS-CoV-2 and COVID-19 Patient Samples for Contemporary Immunology and Metabolomics Studies. *Immunohorizons*, *6*(2), 144-155. https://doi.org/10.4049/immunohorizons.2200005

Eickmann, M., Gravemann, U., Handke, W., Tolksdorf, F., Reichenberg, S., Muller, T. H., & Seltsam, A. (2020). Inactivation of three emerging viruses - severe acute respiratory syndrome coronavirus, Crimean-Congo haemorrhagic fever virus and Nipah virus - in platelet concentrates by ultraviolet C light and in plasma by methylene blue plus visible light. *Vox Sang*, *115*(3), 146-151. https://doi.org/10.1111/vox.12888

Faddy, H. M., Fryk, J. J., Hall, R. A., Young, P. R., Reichenberg, S., Tolksdorf, F., Sumian, C., Gravemann, U., Seltsam, A., & Marks, D. C. (2019). Inactivation of yellow fever virus in plasma after treatment with methylene blue and visible light and in platelet concentrates following treatment with ultraviolet C light. *Transfusion*, *59*(7), 2223-2227. https://doi.org/10.1111/trf.15332

Freeman, S., Kibler, K., Lipsky, Z., Jin, S., German, G. K., & Ye, K. (2022). Systematic evaluating and modeling of SARS-CoV-2 UVC disinfection. *Sci Rep*, *12*(1), 5869. https://doi.org/10.1038/s41598-022-09930-2

Fujimoto, N., Nagaoka, K., Tatsuno, I., Oishi, H., Tomita, M., Hasegawa, T., Tanaka, Y., & Matsumoto, T. (2023). Wavelength dependence of ultraviolet light inactivation for SARS-CoV-2 omicron variants. *Sci Rep*, *13*(1), 9706. https://doi.org/10.1038/s41598-023-36610-6

Fumagalli, M. J., Capato, C. F., de Castro-Jorge, L. A., de Souza, W. M., Arruda, E., & Figueiredo, L. T. M. (2022). Stability of SARS-CoV-2 and other airborne viruses under different stress conditions. *Arch Virol*, *167*(1), 183-187. https://doi.org/10.1007/s00705-021-05293-7

Gracheva, A. V., Korchevaya, E. R., Ammour, Y. I., Smirnova, D. I., Sokolova, O. S., Glukhov, G. S., Moiseenko, A. V., Zubarev, I. V., Samoilikov, R. V., Leneva, I. A., Svitich, O. A., Zverev, V. V., & Faizuloev, E. B. (2022). Immunogenic properties of SARS-CoV-2 inactivated by ultraviolet light. *Arch Virol*, *167*(11), 2181-2191. https://doi.org/10.1007/s00705-022-05530-7

Handke, W., Gravemann, U., Muller, T. H., Wagner, F. F., Schulze, T. J., & Seltsam, A. (2022). New ultraviolet C light-based method for pathogen inactivation of red blood cell units. *Transfusion*, *62*(11), 2314-2323. https://doi.org/10.1111/trf.17098

Kordyukova, L. V., Moiseenko, A. V., Serebryakova, M. V., Shuklina, M. A., Sergeeva, M. V., Lioznov, D. A., & Shanko, A. V. (2023). Structural and Immunoreactivity Properties of the SARS-CoV-2 Spike Protein upon the Development of an Inactivated Vaccine. *Viruses*, *15*(2). https://doi.org/10.3390/v15020480

Lee, C., Park, K. H., Kim, M., & Kim, Y. B. (2022). Optimized parameters for effective SARS-CoV-2 inactivation using UVC-LED at 275 nm. *Sci Rep*, *12*(1), 16664. https://doi.org/10.1038/s41598-022-20813-4

Li, L. X., Nissly, R. H., Swaminathan, A., Bird, I. M., Boyle, N. R., Nair, M. S., Greenawalt, D. I., Gontu, A., Cavener, V. S., Sornberger, T., Freihaut, J. D., Kuchipudi, S. V., & Bahnfleth, W. P. (2023). Inactivation of HCoV-NL63 and SARS-CoV-2 in aqueous solution by 254 nm UV-C. *J Photochem Photobiol B*, *245*, 112755. https://doi.org/10.1016/j.jphotobiol.2023.112755

Lo, C. W., Matsuura, R., Iimura, K., Wada, S., Shinjo, A., Benno, Y., Nakagawa, M., Takei, M., & Aida, Y. (2021). UVC disinfects SARS-CoV-2 by induction of viral genome damage without apparent effects on viral morphology and proteins. *Sci Rep*, *11*(1), 13804. https://doi.org/10.1038/s41598-021-93231-7

Loveday, E. K., Hain, K. S., Kochetkova, I., Hedges, J. F., Robison, A., Snyder, D. T., Brumfield, S. K., Young, M. J., Jutila, M. A., Chang, C. B., & Taylor, M. P. (2021). Effect of Inactivation Methods on SARS-CoV-2 Virion Protein and Structure. *Viruses*, *13*(4). https://doi.org/10.3390/v13040562

Masaike, M., Rattanakul, S., & Oguma, K. (2019). Inactivation of health-related microorganisms in water using UV light-emitting diodes. *Water Supply*, *19*(5), 1507-1514. https://doi.org/10.2166/ws.2019.022

Matsuura, R., Lo, C. W., Ogawa, T., Nakagawa, M., Takei, M., Matsumoto, Y., Wada, S., & Aida, Y. (2022). Comparison of the inactivation capacity of various UV wavelengths

on SARS-CoV-2. *Biochem Biophys Rep*, *32*, 101379. https://doi.org/10.1016/j.bbrep.2022.101379

Nyangaresi, P. O., Rathnayake, T., & Beck, S. E. (2023). Evaluation of disinfection efficacy of single UV-C, and UV-A followed by UV-C LED irradiation on Escherichia coli, B. spizizenii and MS2 bacteriophage, in water. *Sci Total Environ*, *859*(Pt 1), 160256. https://doi.org/10.1016/j.scitotenv.2022.160256

Oh, C., Sun, P. P., Araud, E., & Nguyen, T. H. (2020). Mechanism and efficacy of virus inactivation by a microplasma UV lamp generating monochromatic UV irradiation at 222 nm. *Water Res*, *186*, 116386. https://doi.org/10.1016/j.watres.2020.116386

Patterson, E. I., Prince, T., Anderson, E. R., Casas-Sanchez, A., Smith, S. L., Cansado-Utrilla, C., Solomon, T., Griffiths, M. J., Acosta-Serrano, A., Turtle, L., & Hughes, G. L. (2020). Methods of Inactivation of SARS-CoV-2 for Downstream Biological Assays. *J Infect Dis*, *222*(9), 1462-1467. https://doi.org/10.1093/infdis/jiaa507

Plavec, Z., Domanska, A., Liu, X., Laine, P., Paulin, L., Varjosalo, M., Auvinen, P., Wolf, S. G., Anastasina, M., & Butcher, S. J. (2022). SARS-CoV-2 Production, Purification Methods and UV Inactivation for Proteomics and Structural Studies. *Viruses*, *14*(9). https://doi.org/10.3390/v14091989

Rockey, N., Young, S., Kohn, T., Pecson, B., Wobus, C. E., Raskin, L., & Wigginton, K. R. (2020). UV Disinfection of Human Norovirus: Evaluating Infectivity Using a Genome-Wide PCR-Based Approach. *Environ Sci Technol*, *54*(5), 2851-2858. https://doi.org/10.1021/acs.est.9b05747

Saito, Y., Wada, S., Nagata, K., Makino, H., Boyama, S., Miwa, H., Matsui, S., Kataoka, K., Narita, T., & Horibuchi, K. (2021). Efficiency improvement of AlGaN-based deep-ultraviolet light-emitting diodes and their virus inactivation application. *Japanese Journal of Applied Physics*, *60*(8). https://doi.org/10.35848/1347-4065/ac10f2

Sun, W., Jing, Z., Zhao, Z., Yin, R., Santoro, D., Mao, T., & Lu, Z. (2023). Dose-Response Behavior of Pathogens and Surrogate Microorganisms across the Ultraviolet-C Spectrum: Inactivation Efficiencies, Action Spectra, and Mechanisms. *Environ Sci Technol*, *57*(29), 10891-10900. https://doi.org/10.1021/acs.est.3c00518

Ulloa, S., Bravo, C., Ramirez, E., Fasce, R., & Fernandez, J. (2021). Inactivation of SARS-CoV-2 isolates from lineages B.1.1.7 (Alpha), P.1 (Gamma) and B.1.110 by heating and UV irradiation. *J Virol Methods*, *295*, 114216. https://doi.org/10.1016/j.jviromet.2021.114216

Weyersberg, L., Sommerfeld, F., Vatter, P., & Hessling, M. (2023). UV radiation sensitivity of bacteriophage PhiX174 - A potential surrogate for SARS-CoV-2 in terms of radiation inactivation. *AIMS Microbiol*, *9*(3), 431-443. https://doi.org/10.3934/microbiol.2023023

Yu, M., Gao, R., Lv, X., Sui, M., & Li, T. (2023). Inactivation of phage phiX174 by UV(254) and free chlorine: Structure impairment and function loss. *J Environ Manage*, *340*, 117962. https://doi.org/10.1016/j.jenvman.2023.117962
