# Supplementary material for: Wavelength-Specific UV-C Inactivation of Viruses in Liquids: Dose–Response, Mechanistic Insights, and Structural Integrity—A Systematic Review and Meta-Analysis
Source: Viruses. 2026 Feb 24;18(3):276. doi: 10.3390/v18030276 (PMC13030338; doi:10.3390/v18030276)
Supplement: Supplementary file 1 [file viruses-18-00276-s001.zip › 07_Qualitative_narrative_study_of_inactivation.pdf]

Virus Name Strain	Genome	UV Wavelength (nm)	Inactivation Information
African swine fever virus, BA71V	DNA	254	In liquid porcine plasma, achieving a 4.62 Log_{10} $TCID_{50}/mL$ reduction at 3000 J/L and complete inactivation at 6000 J/L[25].
Bacteriophage Phi6, Pseudomonas phage phi6 (Cystovirus phi6) HER 102	RNA	222<br>265<br>285	Inactivation rate constant of 0.13 cm^2/mJ, significantly higher than at other wavelengths (0.006–0.035 cm^2/mJ), with lipid envelope damage identified as the primary mechanism rather than genome damage[26]. PFU and RT-qPCR, LC-MS. In contrast, genome damage was the leading inactivation mechanism at 265 and 285 nm, but far less efficient.
Bovine viral diarrhea virus, 1-NADL	RNA	254	In plasma (complete at 3000 J/L) is more effective than in red blood cell suspensions (partial at 4.5 J/cm^2)[27,28]. Matrix composition and opacity critically influence inactivation kinetics and required dosage. PFU and $TCID_{50}/mL$ was utilized.
Yellow fever virus, YFV-17D	RNA	254	0.20 J/cm^2, a ≥4.80 log_{10} reduction in viral infectivity was achieved, effectively lowering the virus to below detectable limits using both serial dilution and large-volume plaque assays. Even lower doses (0.10–0.15 J/cm^2) produced ≥3.6 log_{10} reductions, confirming strong dose-dependent inactivation[40]. The UV-C mechanism disrupted YFV's genomic RNA, halting replication and rendering gene/protein expression (e.g., envelope, NS proteins) irrelevant post-treatment.
Vesicular stomatitis virus, Indiana Lab V-520-001-522	RNA	254	A 4.5 J/cm^2 , ≥4.9 log_{10} with further reduction to ≥5.2 log_{10} after post-treatment reconcentration[28]. Genome damage is the main contributor.
Tulane Virus, Tulane virus nonstructural polyprotein, capsid protein, and minor structural protein VP2 genes, complete cds	RNA	222<br>254	Both 220 nm and 254 nm UV-C irradiation, achieving up to 4-log_{10} inactivation at 22.5 mJ/cm^2, with genome damage and loss of capsid function as the main mechanisms. Irradiation mutagenized the NSP gene and disrupted the ability to bind to its host receptor via capsid protein impairment, which prevents both attachment and replication. 254 nm primarily damaged the genome, while 220 nm caused broader disruption, including structural protein degradation, especially VP2 and capsid-binding domains[39].

Swine vesicular disease virus, UK-72	RNA	254	Notable resistance irradiation, a 4D (4-log_{10}) inactivation dose of 3708 J/L. Virus titers dropped significantly at 6000 and 9000 J/L, low residual infectivity (0.02–0.04 particles/mL) was still detected through blind passages, indicating incomplete sterilization at even the highest doses tested[27]. The biphasic inactivation model revealed a "shoulder effect" at lower doses, suggesting delayed inactivation onset, possibly due to aggregation or shielding within plasma.
Swine influenza virus H1N1 A/Swine/Spain/SF11131/2017	RNA	254	Non-linear kinetics (biphasic + shoulder model) with a 4-log reduction dose (4D) of 1639 J/L, complete inactivation by 3000 J/L, with no residual virus detected after subsequent blind passages[27]. Inactivation is possible in protein-rich matrices without affecting plasma functionality.
Sindbis virus AR-339 p270 nonstructural polyprotein, p230 nonstructural polyprotein, hypothetical protein, and truncated polyprotein genes	RNA	254	Mean log_{10} reduction factor was 4.3 after 4.5 J/cm² exposure, improving slightly to 4.6 log_{10} after reconcentration, indicating high efficacy of viral clearance in red blood cells. The inactivation mechanism targets nucleic acids, not specific protein products, thus inactivating replication regardless of specific gene segments[28]. This means that the expression of nonstructural or truncated polyproteins is irrelevant post-treatment, as the genome integrity is compromised, and protein translation is halted.
Seneca virus A, BRA/UEL-PR/15	RNA	254	Baseline titer of 4.68 log_{10} $TCID_{50}$/mL, which dropped to below detection level by 9000 J/L, with a calculated 4D value of 3223 J/L . The best-fitting inactivation model was a biphasic plus shoulder, suggesting an initial delay before rapid decline, possibly due to structural robustness or shielding effects[27]. The inactivation remained incomplete at intermediate doses (1500–3000 J/L).
SARS-CoV, Frankfurt 1	RNA	254	At 0.1 J/cm² (half of the full dose), the virus was reduced below the limit of detection, achieving a ≥3.4 log_{10} $TCID_{50}$/mL reduction . The inactivation was complete without requiring the full dose (0.2 J/cm²)[30].
Rotavirus, OSU	RNA	222<br><br>254	At 220 nm, a dose of 22.5 mJ/cm² resulted in a 5-log_{10} reduction in infectivity, while at 254 nm, the same dose only caused a 2-log_{10} reduction, indicating significantly higher resistance at the longer wavelength. RT-qPCR revealed that 220 nm caused clear damage to the VP7 gene of the genome, while 254 nm UV caused only minimal degradation even at high doses (180 mJ/cm²), suggesting that 254 nm does not efficiently damage genomic segments.

			Despite infectivity loss, host receptor binding remained intact at both wavelengths, indicating that the inactivation mechanism is post-entry, likely affecting genome replication rather than attachment[38].
Pseudorabies virus, Suid herpesvirus 1, strain Aujeszky	DNA	254	In plasma a total dose of 4.5 J/cm² achieved a 2.7-$\log_{10}$ $TCID_{50}$/mL reduction, which slightly increased to 2.9 $\log_{10}$ after reconcentration, likely due to a minor washing effect[28].
Pseudorabies virus, NIA3	DNA	254	In plasma the virus showed susceptibility with a baseline titer of 4.53 $\log_{10}$ $TCID_{50}$/mL reduced to below detection level by 3000 J/L, and a calculated 4D value of 1612 J/L[27]. The best-fit inactivation model was Weibull + tail, suggesting a gradual decline with a minor resistant fraction, although secondary modeling also supported biphasic shoulder interpretation.
Porcine reproductive and respiratory syndrome virus, vP21	RNA	254	4.0 $\log_{10}$ $TCID_{50}$/mL reduced below detection level by 1500 J/L, and a calculated 4D value of 1004 J/L[27]. The inactivation kinetics best fit a log-linear model in this study.
Porcine parvovirus, NADL-2	DNA	254	4-$\log_{10}$ reduction at 2161 J/L in bovine plasma with a biphasic inactivation curve, indicating the presence of a resistant subpopulation and a reduced susceptibility at higher doses. 4.5 J/cm² achieved a 6.2-$\log_{10}$ $TCID_{50}$/mL reduction, demonstrating much higher efficiency due to improved fluid transparency[28]. Matrix composition, genome type, and structural protection (capsid robustness) play major roles in inactivation kinetics.
Porcine epidemic diarrhea virus, CV777	RNA	254	In plasma, with a 4.07 $\log_{10}$ $TCID_{50}$/mL baseline titer reduced below detection level by 3000 J/L, and an estimated 4D value of 1953 J/L, using a biphasic inactivation model as best fit. Infectivity was confirmed by PFU conversion and validated through serial passage of negative samples, confirming full inactivation[27].
Porcine circovirus 2, genotype b isolate Sp-10-7-54-13	DNA	254	Despite being exposed to doses up to 9000 J/L, the virus showed only a 2.71-$\log_{10}$ $TCID_{50}$/mL reduction, and no 4D value could be calculated because the starting titer was <4.0 $\log_{10}$, and full inactivation was not achieved[27]. The inactivation curve followed a biphasic model, indicating a resistant subpopulation, possibly due to genome topology (circular ssDNA) or aggregation effects shielding viral particles. Non-enveloped viruses with small circular genomes may require much higher fluences.
PhiX174, Escherichia phage phiX174	DNA	222<br><br>254	At 222 nm (Far-UVC), a 4.97-$\log_{10}$ PFU/mL reduction was achieved with a total dose of 6.5 mJ/cm², corresponding to a log-reduction dose of 1.3 mJ/cm² and an inactivation rate constant of 0.76 cm²/mJ. At 254 nm, it showed a 4.54-$\log_{10}$ PFU/mL reduction at a dose of 22 mJ/cm², yielding a log-reduction dose of 5.0 mJ/cm² and a rate constant of 0.20 cm²/mJ[37].

Nipah virus, NV/MY/99 /VRI-0626	RNA	254	254 nm irradiation, at 0.15 J/cm² (¾ of full dose) reduced infectivity to the limit of detection, achieving a ≥4.3 $\log_{10}$ $TCID_{50}$/mL reduction[30]. No further reduction was observed with higher doses, implying early saturation and high sensitivity of Nipah virus to both photoinactivation methods.
Mumps virus, Enders	RNA	254	Measurable infectivity even after 300 seconds (total dose: 300 mJ/cm²), complete inactivation was not achieved[29]. The decay of viral titer was modeled using a non-linear one-phase exponential decay curve, demonstrating a consistent but incomplete reduction in $TCID_{50}$/mL over time. No plateau or tailing effect was explicitly reported for Mumps virus, but the persistence of infectivity suggests a relatively resistant subpopulation or structural features limiting photochemical damage.
MS2, 15597-B1 ATCC	RNA	254<br>265<br>280	Firstly, requiring 93.0 mJ/cm² at 267 nm and 126.0 mJ/cm² at 278 nm to achieve ~4.5-$\log_{10}$ inactivation, with rate constants of 0.0541 cm²/mJ (267 nm) and 0.0326 cm²/mJ (278 nm)[35]. Infectivity was quantified via plaque-forming units (PFU/mL), MS2 showed no evidence of repair post-UV exposure, aligning with its lack of enzymatic repair systems. Secondly, resistance in this study using 265 nm and 280 nm, with log-linear inactivation kinetics and no observed shoulder or tailing. The inactivation rate constants were 0.034 cm²/mJ at 265 nm and 0.033 cm²/mJ at 280 nm, showing nearly equivalent susceptibility at both wavelengths. For 3-$\log_{10}$ reduction, the required fluences were 76.1 mJ/cm² (265 nm) and 91.5 mJ/cm² (280 nm)[31]. Thirdly, MS2 bacteriophage genome was used in this study to benchmark a novel qPCR-based inactivation model, and it exhibited an inactivation rate constant of 0.14 ± 0.02 cm²/mJ at 254 nm in optically clear phosphate buffer [34]. Fourthly, varying water qualities using 254 nm in both collimated beam and flow systems, demonstrating a two-phase inactivation profile: a higher rate constant of 0.050 cm²/mJ at low doses (10–90 mJ/cm²) and a lower rate of 0.026 cm²/mJ at higher doses (>90 mJ/cm²), confirming non-linear, biphasic kinetics[36].
Human rhinovirus, A16	RNA	254	Retained substantial infectivity even after 300 seconds (300 mJ/cm²), showing only limited reduction[29].
Human respiratory syncytial virus, ATCC VR-26	RNA	254	Significant reduction after 30 seconds (30 mJ/cm²)[29].
Human Norovirus, GII.4 Sydney	RNA	254	The inactivation rate constant was 0.27 cm²/mJ, and genome degradation was inferred from eight amplicons covering ~50% of the genome, with a strong correlation between amplicon decay and overall genome inactivation[34].
Human Coronavirus, NL63	RNA	254	Inactivation rate constant of 2.062 cm²/mJ, equivalent to 1-log, 2-log, and 3-log reductions at 1.12, 2.25, and 3.37 mJ/cm², respectively[33]. While the data fit a one-stage exponential decay model, higher fluence exposures occasionally showed residual

			infectivity, suggesting tailing or biphasic resistance, despite the model being forced through (0,1). The study emphasized that medium composition (PBS vs DMEM) and stirring affected apparent UV susceptibility due to absorption and mixing effects.
Human adenovirus 2, Adenoid 6	DNA	222<br><br>254	Inactivation rate constant of 0.142 cm²/mJ, which is 4.4 times higher than that of 254 nm UV (0.032 cm²/mJ). Although a log-linear model was fitted, damage saturation for the genome at low fluences and lack complete inactivation suggests non-linear behavior and potential tailing effects[32].
Hepatitis A virus, polyprotein RNA	RNA	254	4.5 J/cm², achieving a $\log_{10}$ reduction of 4.3, with a post-reconcentration result of ≥3.9[28].
Feline calicivirus, FCV-2280		254	4.5 J/cm², under vigorous agitation and low hematocrit conditions to enhanced light penetration. Mean reduction of 3.7 log and increasing to 4.5 log after reconcentration, with no reduction in untreated controls, indicating robust but not complete inactivation[28]. Complete inactivation was not achieved, highlighting a potential plateau effect.
Feline calicivirus, F-9 ATCC® VR-782™	RNA	265<br><br>280	Measured with PFU, Feline calicivirus showed an inactivation rate constants of 0.113 cm²/mJ at 265 nm and 0.101 cm²/mJ at 280 nm, requiring 24.5 mJ/cm² and 28.9 mJ/cm² respectively for a 3-$\log_{10}$ reduction[31]. While the study fitted a log-linear regression, this excluded potential shoulder and tailing regions, meaning the model does not capture full inactivation behavior—especially at higher fluences.
Escherichia coli bacteriophage Q-β	RNA	265<br><br>280	At 265 nm (k = 0.091 cm²/mJ) compared to 280 nm (k = 0.052 cm²/mJ), indicating that shorter wavelengths are more effective[31]. Both wavelengths are assumed to follow a log-linear inactivation profile suggesting uniform susceptibility and a genome-focused inactivation mechanism. While 280 nm is less efficient than 265 nm.
Encephalo myocarditis virus, EMC-D variant	RNA	254	4.5 J/cm² effectively inactivated in PAGGS-C additive solution achieving a $\log_{10}$ reduction factor of 3.1, which slightly improved to 3.4 after reconcentration[28].
Crimean-Congo hemorrhagic fever virus, Afg09-2990	RNA	254	In platelet concentrates[30], the dose of 0.1 J/cm² reduced infectivity below the limit of detection, achieving a virus log reduction factor of ≥2.2, with even higher reduction at the full dose (0.2 J/cm²).
Coxsackievirus, Coxsackievirus B5	RNA	254	300 seconds of exposure to 254 nm light (1 mJ/cm²), its infectivity was only moderately reduced, indicating robust capsid integrity against photochemical damage[29].
Classical swine fever	RNA	254	Inactivated at doses below 3000 J/L, with a calculated 4-log reduction (4D) dose of 1641 J/L, is highly efficient under turbulent flow conditions[27]. The inactivation curve followed a biphasic plus

virus, Alfort/187			shoulder model, indicating a non-linear but robust decline in viral load. The study suggests that enveloped viruses are more susceptible than non-enveloped ones. PFU and $TCID_{50}$/mL was utilized.

References:

1 Chiappa, F. et al. The efficacy of ultraviolet light-emitting technology against coronaviruses: a systematic review. J Hosp Infect 114, 63-78, doi:10.1016/j.jhin.2021.05.005 (2021).

2 Marasini, S., Zhang, A. C., Dean, S. J., Swift, S. & Craig, J. P. Safety and efficacy of UV application for superficial infections in humans: A systematic review and meta-analysis. Ocul Surf 21, 331-344, doi:10.1016/j.jtos.2021.03.002 (2021).

3 Lindblad, M., Tano, E., Lindahl, C. & Huss, F. Ultraviolet-C decontamination of a hospital room: Amount of UV light needed. Burns 46, 842-849, doi:10.1016/j.burns.2019.10.004 (2020).

4 Song, K., Mohseni, M. & Taghipour, F. Application of ultraviolet light-emitting diodes (UV-LEDs) for water disinfection: A review. Water Res 94, 341-349, doi:10.1016/j.watres.2016.03.003 (2016).

5 Sabbaghi, A., Miri, S. M., Keshavarz, M., Zargar, M. & Ghaemi, A. Inactivation methods for whole influenza vaccine production. Rev Med Virol 29, e2074, doi:10.1002/rmv.2074 (2019).

6 Loveday, E. K. et al. Effect of Inactivation Methods on SARS-CoV-2 Virion Protein and Structure. Viruses 13, doi:10.3390/v13040562 (2021).

7 Feys, H. B. et al. Oxygen removal during pathogen inactivation with riboflavin and UV light preserves protein function in plasma for transfusion. Vox Sang 106, 307-315, doi:10.1111/vox.12106 (2014).

8 Rule Wigginton, K., Menin, L., Montoya, J. P. & Kohn, T. Oxidation of Virus Proteins during UV254 and Singlet Oxygen Mediated Inactivation. Environmental Science & Technology 44, 5437-5443, doi:10.1021/es100435a (2010).

9 Wang, J. et al. Virus inactivation and protein recovery in a novel ultraviolet-C reactor. Vox Sang 86, 230-238, doi:10.1111/j.0042-9007.2004.00485.x (2004).

10 Szabo, P. et al. Systematic review: pain, cognition, and cardioprotection-unpacking oxytocin's contributions in a sport context. Front Physiol 15, 1393497, doi:10.3389/fphys.2024.1393497 (2024).

11 Sterne, J. A. et al. ROBINS-I: a tool for assessing risk of bias in non-randomised studies of interventions. BMJ 355, i4919, doi:10.1136/bmj.i4919 (2016).

12 Jonathan Sterne, J. H. The Risk Of Bias In Non-randomized Studies – of Interventions, Version 2 (ROBINS-I V2), <https://www.riskofbias.info/welcome/robins-i-v2> (2024).

13 McGuinness, L. A. & Higgins, J. P. T. Risk-of-bias VISualization (robvis): An R package and Shiny web app for visualizing risk-of-bias assessments. Res Synth Methods 12, 55-61, doi:10.1002/jrsm.1411 (2021).

14 Page, M. J. et al. The PRISMA 2020 statement: an updated guideline for reporting systematic reviews. BMJ 372, n71, doi:10.1136/bmj.n71 (2021).

15 Hoffmann, T. C. et al. Better reporting of interventions: template for intervention description and replication (TIDieR) checklist and guide. BMJ 348, g1687, doi:10.1136/bmj.g1687 (2014).

16 Zhang, Y., Akl, E. A. & Schunemann, H. J. Using systematic reviews in guideline development: the GRADE approach. Res Synth Methods 10, doi:10.1002/jrsm.1313 (2019).

17 Andersson, T., Alfredsson, L., Kallberg, H., Zdravkovic, S. & Ahlbom, A. Calculating measures of biological interaction. Eur J Epidemiol 20, 575-579, doi:10.1007/s10654-005-7835-x (2005).

18 Field, A. P. & Gillett, R. How to do a meta-analysis. Br J Math Stat Psychol 63, 665-694, doi:10.1348/000711010X502733 (2010).

19 Sen, S. & Yildirim, I. A Tutorial on How to Conduct Meta-Analysis with IBM SPSS Statistics. Psych 4, 640-667, doi:10.3390/psych4040049 (2022).

20 Kitagawa, H. et al. Effect of intermittent irradiation and fluence-response of 222 nm ultraviolet light on SARS-CoV-2 contamination. Photodiagnosis Photodyn Ther 33, 102184, doi:10.1016/j.pdpdt.2021.102184 (2021).

21 Kitagawa, H. et al. Effectiveness of 222-nm ultraviolet light on disinfecting SARS-CoV-2 surface contamination. Am J Infect Control 49, 299-301, doi:10.1016/j.ajic.2020.08.022 (2021).

22 Park, S., Perlin, D. S., Fitzgerald, S., Petraitis, V. & Walsh, T. J. Focused multivector ultraviolet (FMUV) technology rapidly eradicates SARS-CoV-2 in-vitro: Implications for hospital disinfection of COVID-19 environments. Am J Infect Control 50, 828-830, doi:10.1016/j.ajic.2022.02.001 (2022).

23 Zhao, Y. & Dong, J. Effect of inactivating RNA viruses by coupled UVC and UVA LEDs evaluated by a viral surrogate commonly used as a genetic vector. Biomed Opt Express 13, 4429-4444, doi:10.1364/BOE.468445 (2022).

24 Storm, N. et al. Rapid and complete inactivation of SARS-CoV-2 by ultraviolet-C irradiation. Sci Rep 10, 22421, doi:10.1038/s41598-020-79600-8 (2020).

25 Blazquez, E. et al. Effect of spray-drying and ultraviolet C radiation as biosafety steps for CSFV and ASFV inactivation in porcine plasma. PLoS One 16, e0249935, doi:10.1371/journal.pone.0249935 (2021).

26 Sun, W. et al. Dose-Response Behavior of Pathogens and Surrogate Microorganisms across the Ultraviolet-C Spectrum: Inactivation Efficiencies, Action Spectra, and Mechanisms. Environ Sci Technol 57, 10891-10900, doi:10.1021/acs.est.3c00518 (2023).

27 Blazquez, E. et al. Evaluation of the effectiveness of the SurePure Turbulator ultraviolet-C irradiation equipment on inactivation of different enveloped and non-enveloped viruses inoculated in commercially collected liquid animal plasma. PLoS One 14, e0212332, doi:10.1371/journal.pone.0212332 (2019).

28 Handke, W. et al. New ultraviolet C light-based method for pathogen inactivation of red blood cell units. Transfusion 62, 2314-2323, doi:10.1111/trf.17098 (2022).

29 Fumagalli, M. J. et al. Stability of SARS-CoV-2 and other airborne viruses under different stress conditions. Arch Virol 167, 183-187, doi:10.1007/s00705-021-05293-7 (2022).

30 Eickmann, M. et al. Inactivation of three emerging viruses - severe acute respiratory syndrome coronavirus, Crimean-Congo haemorrhagic fever virus and Nipah virus - in platelet concentrates by ultraviolet C light and in plasma by methylene blue plus visible light. Vox Sang 115, 146-151, doi:10.1111/vox.12888 (2020).

31 Masaike, M., Rattanakul, S. & Oguma, K. Inactivation of health-related microorganisms in water using UV light-emitting diodes. Water Supply 19, 1507-1514, doi:10.2166/ws.2019.022 (2019).

32 Oh, C., Sun, P. P., Araud, E. & Nguyen, T. H. Mechanism and efficacy of virus inactivation by a microplasma UV lamp generating monochromatic UV irradiation at 222 nm. Water Res 186, 116386, doi:10.1016/j.watres.2020.116386 (2020).

33 Li, L. X. et al. Inactivation of HCoV-NL63 and SARS-CoV-2 in aqueous solution by 254 nm UV-C. J Photochem Photobiol B 245, 112755, doi:10.1016/j.jphotobiol.2023.112755 (2023).

34 Rockey, N. et al. UV Disinfection of Human Norovirus: Evaluating Infectivity Using a Genome-Wide PCR-Based Approach. Environ Sci Technol 54, 2851-2858, doi:10.1021/acs.est.9b05747 (2020).

35 Nyangaresi, P. O., Rathnayake, T. & Beck, S. E. Evaluation of disinfection efficacy of single UV-C, and UV-A followed by UV-C LED irradiation on Escherichia coli, B. spizizenii and MS2 bacteriophage, in water. Sci Total Environ 859, 160256, doi:10.1016/j.scitotenv.2022.160256 (2023).

36 Baldasso, V. et al. UVC inactivation of MS2-phage in drinking water - Modelling and field testing. Water Res 203, 117496, doi:10.1016/j.watres.2021.117496 (2021).

37 Weyersberg, L., Sommerfeld, F., Vatter, P. & Hessling, M. UV radiation sensitivity of bacteriophage PhiX174 - A potential surrogate for SARS-CoV-2 in terms of radiation inactivation. AIMS Microbiol 9, 431-443, doi:10.3934/microbiol.2023023 (2023).

38 Araud, E., Fuzawa, M., Shisler, J. L., Li, J. & Nguyen, T. H. UV Inactivation of Rotavirus and Tulane Virus Targets Different Components of the Virions. Applied and Environmental Microbiology 86, e02436-02419, doi:doi:10.1128/AEM.02436-19 (2020).

39 Araud, E., Fuzawa, M., Shisler, J. L., Li, J. & Nguyen, T. H. UV Inactivation of Rotavirus and Tulane Virus Targets Different Components of the Virions. Appl Environ Microbiol 86, doi:10.1128/AEM.02436-19 (2020).

40 Faddy, H. M. et al. Inactivation of yellow fever virus in plasma after treatment with methylene blue and visible light and in platelet concentrates following treatment with ultraviolet C light. Transfusion 59, 2223-2227, doi:10.1111/trf.15332 (2019).

41 Biffi, S. et al. Determination of the UV Inactivation Constant Under 280 nm UV LED Irradiation for SARS-CoV-2. Photochem Photobiol 99, 101-105, doi:10.1111/php.13653 (2023).

42 Sesti-Costa, R. et al. UV 254 nm is more efficient than UV 222 nm in inactivating SARS-CoV-2 present in human saliva. Photodiagnosis Photodyn Ther 39, 103015, doi:10.1016/j.pdpdt.2022.103015 (2022).
