# Supplementary material for: Wavelength-Specific UV-C Inactivation of Viruses in Liquids: Dose–Response, Mechanistic Insights, and Structural Integrity—A Systematic Review and Meta-Analysis
Source: Viruses. 2026 Feb 24;18(3):276. doi: 10.3390/v18030276 (PMC13030338; doi:10.3390/v18030276)
Supplement: Supplementary file 1 [file viruses-18-00276-s001.zip › 08_RoB_Assessment_Figures.pdf]

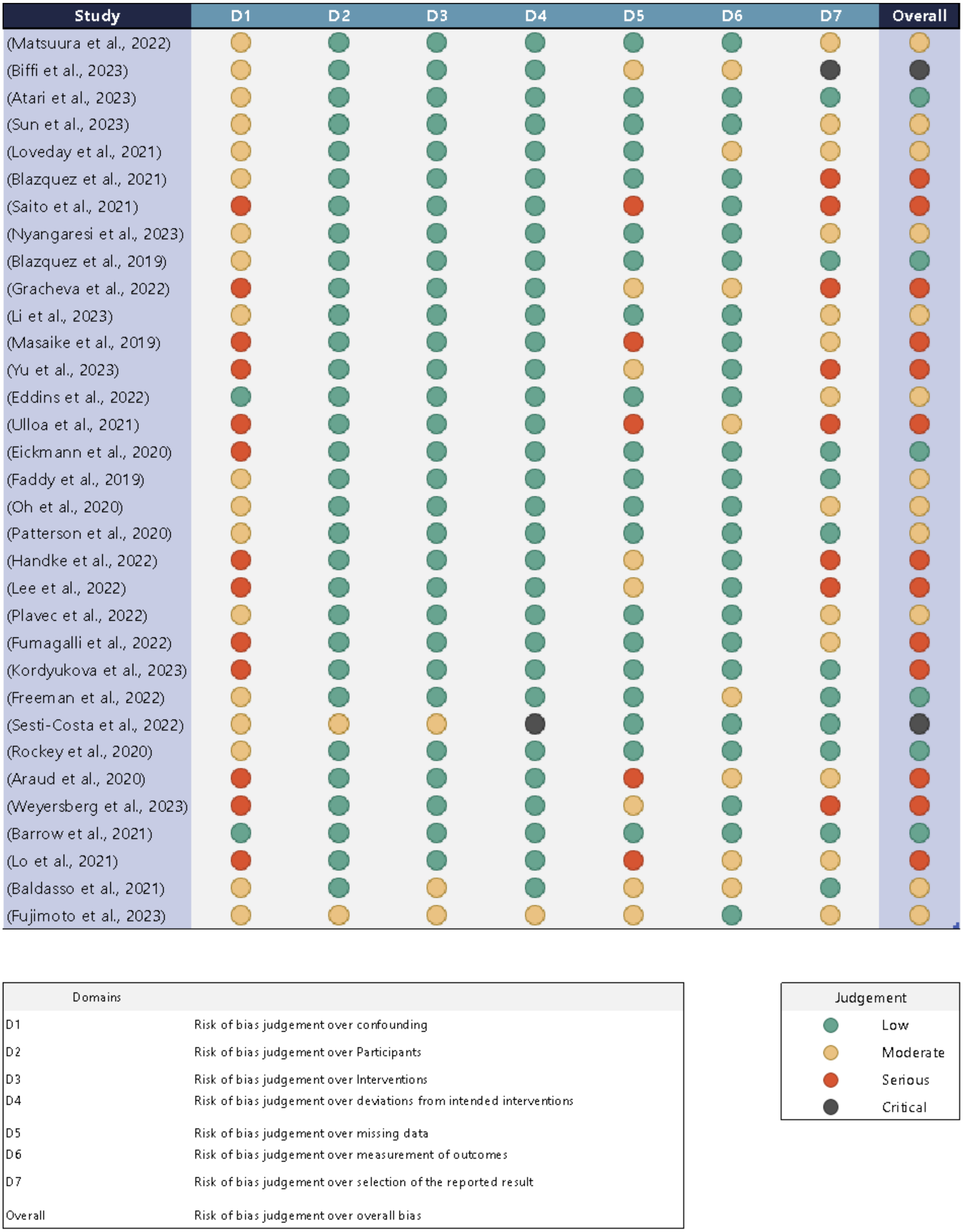

Figure 1. Shows Risk of Bias assessment using ROBINS-I V2 across seven domains (D1–D7) for 33 studies.

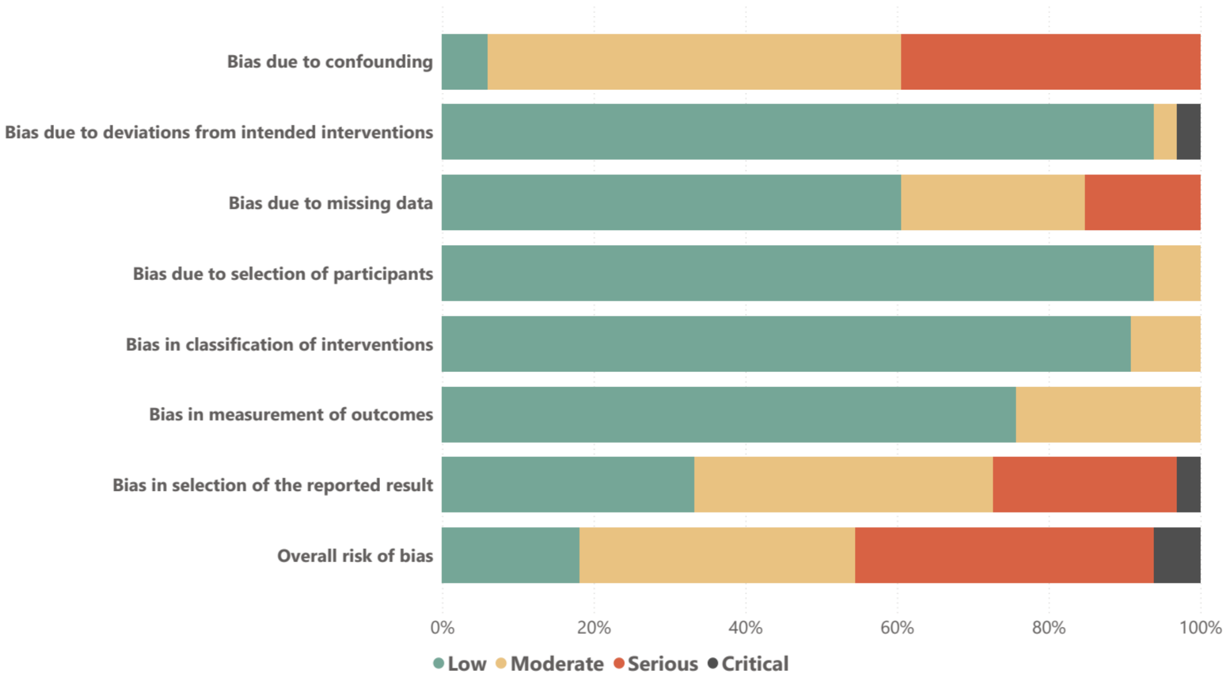

Domains	
D1	Risk of bias judgement over confounding
D2	Risk of bias judgement over Participants
D3	Risk of bias judgement over Interventions
D4	Risk of bias judgement over deviations from intended interventions
D5	Risk of bias judgement over missing data
D6	Risk of bias judgement over measurement of outcomes
D7	Risk of bias judgement over selection of the reported result
Overall	Risk of bias judgement over overall bias

Judgement
Low
Moderate
Serious
Critical

Figure 2. Shows summary plot of ROBINS-I V2 domain ratings across included studies. Most bias arose from confounding and selection of reported results
