# Supplementary material for: Wavelength-Specific UV-C Inactivation of Viruses in Liquids: Dose–Response, Mechanistic Insights, and Structural Integrity—A Systematic Review and Meta-Analysis
Source: Viruses. 2026 Feb 24;18(3):276. doi: 10.3390/v18030276 (PMC13030338; doi:10.3390/v18030276)
Supplement: Supplementary file 1 [file viruses-18-00276-s001.zip › 09_GRADE_recommendations_policy.pdf]

Summary of findings:

Different operational parameters (e.g., wavelength, dose) compared to UV-C light for Viruses in suspensions

Patient or population: Viruses in suspensions
Setting: Experimental virology using UV-C exposure in liquid suspensions (e.g., PBS, VERO E6 supernatants) in petri dishes or tubes
Intervention: Different operational parameters (e.g., wavelength, dose)
Comparison: UV-C light

Outcomes	Anticipated absolute effects* (95% CI)		Relative effect (95% CI)	№ of participants (studies)	Certainty of the evidence (GRADE)	Comments
	**Risk with UV-C light**	**Risk with Different operational parameters (e.g., wavelength, dose)**				
Log_{10} Reduction (UV-C) (Reduction) assessed with: TCID50, PFU	The mean log_{10} Reduction (UV-C) was **2.95** log reduction	**0 log reduction** (2.946 higher to 3.817 higher)	-	34 (17 non-randomised studies)	⊕⊕⊕⊕ High[a,b,c]	Effect based on random-effects meta-regression (N = 114, REML, Knapp-Hartung adjustment); R^2 = 95.2%; CI from filtered model.

***The risk in the intervention group** (and its 95% confidence interval) is based on the assumed risk in the comparison group and the **relative effect** of the intervention (and its 95% CI).

CI: confidence interval

GRADE Working Group grades of evidence
High certainty: we are very confident that the true effect lies close to that of the estimate of the effect.
Moderate certainty: we are moderately confident in the effect estimate: the true effect is likely to be close to the estimate of the effect, but there is a possibility that it is substantially different.
Low certainty: our confidence in the effect estimate is limited: the true effect may be substantially different from the estimate of the effect.
Very low certainty: we have very little confidence in the effect estimate: the true effect is likely to be substantially different from the estimate of effect.

Explanations

a. Inconsistencies across studies regarding protocol transparency and result plausibility further complicate interpretation. Overall, while the findings offer valuable insights, imprecision and bias risks limit their reliability without cautious, context-aware interpretation.
b. However, a consistent lack of pre-registered analysis plans raises concerns about selective reporting and indirectness in findings. Confounding is a recurring issue, often unaddressed, leading to potential overestimation or underestimation of UV-C efficacy.
c. The study generally uses controlled experimental designs and standardized measurements, which help reduce some sources of bias.

QUESTION

**Should Different operational parameters (e.g., wavelength, dose) vs. UV-C light be used for Viruses in suspensions?**	
**POPULATION:**	Viruses in suspensions
**INTERVENTION:**	Different operational parameters (e.g., wavelength, dose)
**COMPARISON:**	UV-C light
**MAIN OUTCOMES:**	Log_{10} Reduction (UV-C);
**SETTING:**	Experimental virology using UV-C exposure in liquid suspensions (e.g., PBS, VERO E6 supernatants) in petri dishes or tubes
**PERSPECTIVE:**	This assessment was conducted from a public health and biosafety perspective, with relevance to clinical laboratories, biotechnology research, infection control, and vaccine development sectors.
**BACKGROUND:**	UV-C light is a widely studied non-chemical disinfection method with demonstrated potential for viral inactivation. However, current literature shows methodological inconsistencies and lacks standardization in experimental setup and reporting. This review systematically assessed the efficacy of UV-C on RNA and DNA viruses in liquid solutions to support evidence-informed implementation and future standardization.
**CONFLICT OF INTERESTS:**	The authors declare no relevant financial or personal conflicts of interest. Two contributors are employed by RoLink Biotechnology Ltd., which had no influence on the design, conduct, or interpretation of the review.

ASSESSMENT

Problem
Is the problem a priority?

JUDGEMENT	RESEARCH EVIDENCE	ADDITIONAL CONSIDERATIONS
○ No<br>○ Probably no<br>○ Probably yes<br>● Yes<br>○ Varies<br>○ Don't know	The problem of viral inactivation using UV-C light is a high-priority issue due to its implications in public health, biotechnology, and clinical safety. Our systematic review of 33 studies (17 meta-analyzed) confirms that UV-C within 100–280 nm can significantly reduce viral loads across both RNA and DNA viruses in liquid solutions. However, current experimental designs lack standardization and clarity in terminology, especially concerning “inactivation” vs. “disinfection.” These inconsistencies hinder the translation of laboratory findings to real-world application and necessitate a refined protocol for UV-C evaluation.	Global health emergencies (e.g., SARS-CoV-2) have emphasized the need for reliable and replicable disinfection strategies. The growing use of UV-C technologies in healthcare, water treatment, and vaccine development underlines the societal and economic importance of this research. Moreover, the potential of UV-C to preserve viral structural integrity while inactivating replication capacity presents opportunities for safer vaccine production and protein studies.

Desirable Effects
How substantial are the desirable anticipated effects?

JUDGEMENT	RESEARCH EVIDENCE	ADDITIONAL CONSIDERATIONS
○ Trivial<br>○ Small<br>○ Moderate<br>● Large<br>○ Varies<br>○ Don't know	The review identifies significant reductions in viral load across diverse virus types with UV-C exposure, including up to 7-log reductions (99.99999%) in some studies. UV-C treatment, when standardized, enables safe manipulation of inactivated viruses while preserving structural integrity, a critical advance for vaccine production, biosample handling, and biosafety applications. The ability to inactivate pathogens without damaging proteins opens possibilities for virological assays and structural biology research.	Given the global need for scalable disinfection tools and safe virus-handling protocols, these effects support improved public health outcomes, especially during outbreaks or in clinical and laboratory environments. The potential for broader biotechnological innovation further enhances the desirability of these effects.

Undesirable Effects
How substantial are the undesirable anticipated effects?

JUDGEMENT	RESEARCH EVIDENCE	ADDITIONAL CONSIDERATIONS

○ Trivial<br>○ Small<br>○ Moderate<br>● Large<br>○ Varies<br>○ Don't know	Our review identified a moderate level of undesirable effects primarily stemming from poor standardization across studies. Incomplete inactivation due to incorrect UV-C dosages, inadequate exposure times, or poorly described setups poses a tangible biosafety risk. Several studies lacked sufficient reporting on variables such as lamp intensity, exposure configuration, or viral load quantification, making reproducibility and safety assessment difficult. This lack of rigor may lead to false assumptions about inactivation and potential accidental exposure to viable pathogens. These optimalizations are crucial for biosafety.	In laboratory and clinical contexts, these risks are non-trivial and must be addressed through protocol harmonization and stricter reporting guidelines. Improved methodological consistency is essential to mitigate accidents and ensure reliable viral inactivation. These optimalizations are crucial for biosafety.

Certainty of evidence

What is the overall certainty of the evidence of effects?

JUDGEMENT	RESEARCH EVIDENCE	ADDITIONAL CONSIDERATIONS
○ Very low<br>○ Low<br>● Moderate<br>○ High<br>○ No included studies	Although the included studies consistently show that UV-C light can effectively inactivate viruses, many were non-randomized with varying methodological quality. Risk of bias was generally low to moderate, but inconsistencies in experimental setups, UV dosimetry, and reporting practices reduced the overall confidence. The lack of a unified protocol and variance in buffer types, virus strains, and dose calculations contributed to heterogeneity. Despite this, the magnitude of effects was strong and biologically plausible across studies.	The evidence base is sufficient for drawing preliminary conclusions, but not yet robust enough for high-certainty recommendations without standardization and better-controlled comparisons.

Values

Is there important uncertainty about or variability in how much people value the main outcomes?

JUDGEMENT	RESEARCH EVIDENCE	ADDITIONAL CONSIDERATIONS
○ Important uncertainty or variability<br>○ Possibly important uncertainty or variability<br>● Probably no important uncertainty or variability<br>○ No important uncertainty or variability	Effective viral inactivation is broadly valued across sectors—from hospitals and labs to biotechnology industries—due to its direct role in biosafety, infection control, and vaccine production. The public, health professionals, and researchers all recognize the critical importance of reliable pathogen inactivation, especially following the COVID-19 pandemic. Our review's focus on structural preservation further adds value in domains where intact viral proteins are necessary, such as serology or immunogenicity studies.	Given this wide applicability and general agreement on its utility, significant variability in stakeholder valuation is unlikely.

Balance of effects

Does the balance between desirable and undesirable effects favor the intervention or the comparison?

JUDGEMENT	RESEARCH EVIDENCE	ADDITIONAL CONSIDERATIONS
○ Favors the comparison<br>● Probably favors the comparison<br>○ Does not favor either the intervention or the comparison<br>○ Probably favors the intervention<br>○ Favors the intervention<br>○ Varies<br>○ Don't know	Desirable effects such as reliable inactivation, structural protein preservation, and potential application in vaccine production clearly outweigh the main undesirable effects, which stem from poor standardization and methodological inconsistency. While these drawbacks are non-negligible, they are avoidable with controlled setups and improved reporting. No direct comparison (e.g. chemical disinfection or heat) was made in your review, but UV-C remains uniquely suited for applications requiring intact viral proteins.	With refinement, UV-C inactivation offers scalable, precise, and low-residue pathogen control that current alternatives may not match in all contexts.

Resources required

JUDGEMENT	RESEARCH EVIDENCE	ADDITIONAL CONSIDERATIONS

○ Large costs<br>○ Moderate costs<br>○ Negligible costs and savings<br>○ Moderate savings<br>● Large savings<br>○ Varies<br>○ Don't know	UV-C systems—especially LEDs and low-pressure mercury lamps—are increasingly affordable and reusable, offering cost-effective disinfection without consumables like chemicals or filters. The intervention enables rapid inactivation, reducing labor time, equipment turnover, and waste. Additionally, preserving viral structure reduces reliance on more expensive cold-inactivation or proteomic purification techniques in biotechnology and vaccine research.	Once implemented, UV-C systems yield long-term operational savings in hospitals, labs, and industrial settings through reduced pathogen spread and minimized consumable usage.

Certainty of evidence of required resources

What is the certainty of the evidence of resource requirements (costs)?

JUDGEMENT	RESEARCH EVIDENCE	ADDITIONAL CONSIDERATIONS
○ Very low<br>○ Low<br>● Moderate<br>○ High<br>○ No included studies	While no cost-effectiveness studies were directly included in your systematic review, substantial literature and market data confirm the affordability and scalability of UV-C light technologies. Variability exists between UV-C LEDs and traditional mercury lamps, and cost information is often context-specific, lacking formal economic analysis. However, real-world implementations consistently report favorable cost-benefit outcomes in clinical and lab settings.	With improving technology and economies of scale, cost certainty is strengthening but not yet fully backed by systematic economic reviews.

Cost effectiveness

Does the cost-effectiveness of the intervention favor the intervention or the comparison?

JUDGEMENT	RESEARCH EVIDENCE	ADDITIONAL CONSIDERATIONS
○ Favors the comparison<br>○ Probably favors the comparison<br>○ Does not favor either the intervention or the comparison<br>● Probably favors the intervention<br>○ Favors the intervention<br>○ Varies<br>○ No included studies	Although formal health economic studies were not included, UV-C systems are generally cost-efficient due to their long lifespan, low maintenance, and minimal consumable use. Compared to chemical or thermal methods, UV-C offers repeatable, rapid, and scalable disinfection that reduces labor costs and downtime. Especially in lab and clinical environments, the combination of high efficacy and reusability makes it economically attractive.	While precise comparative cost-effectiveness data are limited, the qualitative evidence and market trends support its economic favorability for targeted applications.

Equity

What would be the impact on health equity?

JUDGEMENT	RESEARCH EVIDENCE	ADDITIONAL CONSIDERATIONS
○ Reduced<br>○ Probably reduced<br>○ Probably no impact<br>● Probably increased<br>○ Increased<br>○ Varies<br>○ Don't know	UV-C disinfection/inactivation, especially with declining costs of UV-LEDs and simplified devices, can be implemented in low-resource settings without recurring supply chains or chemical dependencies. Its scalability and one-time investment model make it accessible to underserved regions that lack stable infrastructure for chemical disinfection. By enabling safe virus handling and surface decontamination, UV-C helps reduce infectious disease burdens more equitably.	Further increases in equity will depend on local manufacturing, maintenance support, and global knowledge-sharing on safe and standardized use.

Acceptability

Is the intervention acceptable to key interest-holders?

JUDGEMENT	RESEARCH EVIDENCE	ADDITIONAL CONSIDERATIONS
○ No<br>○ Probably no<br>○ Probably yes<br>● Yes<br>○ Varies<br>○ Don't know	UV-C interventions are widely accepted in clinical, laboratory, industrial, and public health contexts due to their non-invasive nature, rapid action, and growing availability. Stakeholders value its safety (when used properly), low operational cost, and effectiveness. Its integration into hospital disinfection protocols and biosafety procedures suggests strong institutional acceptance.	Concerns over improper use or eye/skin exposure exist but are well-managed through clear safety protocols and shielding, further supporting its acceptability.

Feasibility

Is the intervention feasible to implement?

JUDGEMENT	RESEARCH EVIDENCE	ADDITIONAL CONSIDERATIONS

○ No<br>○ Probably no<br>● Probably yes<br>○ Yes<br>○ Varies<br>○ Don't know	UV-C disinfection technologies are already in widespread use in hospitals, labs, and water treatment facilities, demonstrating proven feasibility. Devices range from handheld units to automated systems and can be tailored to resource availability and target pathogens. The main barriers—like safety training and setup calibration—are manageable with standard protocols and advanced infrastructure - through education and science.	Increasing availability of portable, affordable UV-C systems enhances implementation even in lower-resource settings, supporting scalability and feasibility.

SUMMARY OF JUDGEMENTS

	JUDGEMENT						
**PROBLEM**	No	Probably no	Probably yes	**Yes**		Varies	Don't know
**DESIRABLE EFFECTS**	Trivial	Small	Moderate	**Large**		Varies	Don't know
**UNDESIRABLE EFFECTS**	Trivial	Small	Moderate	**Large**		Varies	Don't know
**CERTAINTY OF EVIDENCE**	Very low	Low	**Moderate**	High			No included studies
**VALUES**	Important uncertainty or variability	Possibly important uncertainty or variability	**Probably no important uncertainty or variability**	No important uncertainty or variability			
**BALANCE OF EFFECTS**	Favors the comparison	**Probably favors the comparison**	Does not favor either the intervention or the comparison	Probably favors the intervention	Favors the intervention	Varies	Don't know
**RESOURCES REQUIRED**	Large costs	Moderate costs	Negligible costs and savings	Moderate savings	**Large savings**	Varies	Don't know
**CERTAINTY OF EVIDENCE OF REQUIRED RESOURCES**	Very low	Low	**Moderate**	High			No included studies
**COST EFFECTIVENESS**	Favors the comparison	Probably favors the comparison	Does not favor either the intervention or the comparison	**Probably favors the intervention**	Favors the intervention	Varies	No included studies
**EQUITY**	Reduced	Probably reduced	Probably no impact	**Probably increased**	Increased	Varies	Don't know
**ACCEPTABILITY**	No	Probably no	Probably yes	**Yes**		Varies	Don't know
**FEASIBILITY**	No	Probably no	**Probably yes**	Yes		Varies	Don't know

TYPE OF RECOMMENDATION

Strong recommendation against the intervention	Conditional recommendation against the intervention	Conditional recommendation for either the intervention or the comparison	Conditional recommendation for the intervention	**Strong recommendation for the intervention**
○	○	○	○	●

CONCLUSIONS

Recommendation

We recommend the implementation of UV-C light-based viral inactivation systems in research and clinical environments, provided standardized protocols and safety measures are in place, with our advanced reporting table for transparance and reiliability, and pre-registration of protocols.

Justification

Overall justification
UV-C interventions demonstrate large desirable effects—such as effective viral load reduction and preservation of viral structure—while posing only moderate risks due to methodological inconsistency. The intervention is cost-effective, acceptable to stakeholders, and feasible in diverse settings, with probable benefits for health equity. Moderate certainty of evidence supports this recommendation, though further standardization and high-quality studies would enhance confidence.

Detailed justification

Cost effectiveness

Properly inactivated and intact viral structures may advance the biotechnological field rapidly.

Subgroup considerations

Special attention should be given to differences in UV-C sensitivity among viral subtypes, the design of inactivation systems. Protocol adjustments may be needed for high-containment labs, low-resource facilities, and specific viral families.

Implementation considerations

Standardized protocols must be developed for UV-C dose, wavelength, exposure time, and equipment calibration, with consistent reporting. Proper shielding and safety training are essential to avoid harm to operators. Implementation should consider the type of viral targets (RNA/DNA, enveloped/non-enveloped) and the configuration of the exposure system (open vs. contained). Integration with existing biosafety frameworks and compatibility with local infrastructure must be addressed.

Monitoring and evaluation

Continuous evaluation of UV-C system efficacy should include regular performance testing (e.g. dosimetry, inactivation verification using TCID50/PFU). Adverse events or near misses should be tracked through biosafety reporting systems. A checklist-based auditing system could be introduced for labs using UV-C for viral inactivation to ensure compliance and quality control.

Research priorities

Future research should focus on direct comparative studies of UV-C with other inactivation methods, quantifying residual infectivity, and evaluating long-term protein integrity post-irradiation. High-quality studies are needed on dose–response relationships across diverse viral families. Development of a reporting standard for UV-C inactivation studies (similar to CONSORT or ARRIVE) would greatly enhance reproducibility and meta-analysis reliability which we have begun in this work.

1. Abstract

Background:

UV-C irradiation is widely used to inactivate viruses in clinical and environmental settings, yet inconsistencies in experimental setups, terminology, and outcome definitions hinder standardization.

Objective:

This review aimed to evaluate the spectral efficacy of UV-C light (100–280 nm) on virus inactivation in liquid solutions, determine optimal wavelengths, and propose a refined definition of "complete inactivation."

Methods:

We included 33 non-randomized intervention studies meeting strain-level virus identification and UV setup criteria. Meta-regression (N=17 studies) and thematic linguistic analysis were conducted using SPSS and Power BI.

Results:

UV-C irradiation showed wavelength- and strain-specific efficacy. 267 nm and 275 nm wavelengths demonstrated superior viral inactivation ($\beta = 6.418$; $\beta = 3.776$), with LOG_DOSE as a consistent predictor ($\beta = 3.382$, $I^2 = 15.1\%$).

Conclusion:

UV-C effectiveness is influenced by wavelength, matrix, and virus strain. We propose standard reporting practices and a rigorous definition for “complete inactivation” to enhance biosafety and translational research.

2. Executive summary

2.1. Methods

Methods

- Databases: PubMed, Scopus, Embase, Web of Science, Ovid (search date: 2024.04.10)
- Inclusion: Studies reporting viral strain, UV-C setup, and quantitative inactivation ($TCID_{50}$/PFU)
- Tools: PRISMA, ROBINS-I V2, Power BI, SPSS
- Meta-regression modeled LOG_DOSE vs LOG_reduction; moderators: virus strain, UV wavelength
- Sensitivity: Robust SEs (HC3), bootstrap CI (BCa, 1000 samples)

Interpretation of Strong and Conditional Recommendations

Strong Recommendation:

Use 254–275 nm UV-C light in controlled laboratory settings for viral inactivation, with 267 nm showing highest efficacy. Ensure accurate reporting of UV dose, assay method, and matrix transparency.

Conditional Recommendation:

Adopt 222 nm UV-C for clinical and public environments with caution due to incomplete proteomic damage data and potential human exposure risks. Standardize definitions (e.g., “inactivation” vs “disinfection”) and require multi-assay confirmation for regulatory approval.

2.2. Interpretation of strong and conditional recommendations

Strong Recommendations:

UV-C irradiation at 254–275 nm, especially at 267 nm, should be used in laboratory and controlled disinfection settings due to its consistently high efficacy in reducing viral loads. These wavelengths demonstrated strong, dose-dependent inactivation effects and are supported by robust statistical evidence. Experimental protocols should mandate strain-level virus identification, dose standardization (log-transformed), and validated infectivity assays to confirm inactivation.

Conditional Recommendations:

Use of 222 nm UV-C light, while promising for surface disinfection and safe human exposure, should be applied with caution due to less consistent viral inactivation data and limited information on proteomic damage. Incomplete reporting and matrix effects (e.g., sample turbidity, suspension media) necessitate careful evaluation. Regulatory and practical guidelines should differentiate "inactivation" for lab studies from "disinfection" in applied fields, requiring orthogonal assay confirmation for high-biosafety contexts.

2.3. How to use these guidelines

These guidelines are intended for researchers, biosafety officers, and public health professionals applying UV-C light for viral inactivation or disinfection. For laboratory use, follow the strong recommendations by selecting optimal wavelengths (254–275 nm) and validating viral reduction with sensitive infectivity assays (e.g., $TCID_{50}$, plaque assay, qPCR). For real-world applications such as water treatment or hospital disinfection, conditional recommendations suggest cautious use of 222 nm UV-C with detailed risk assessments. Ensure all protocols report UV source details, matrix characteristics, and dose-response data transparently. Incorporating these standards will enhance reproducibility, biosafety, and the translational potential of UV-C technologies.

2.4. Recommendations

Use UV-C wavelengths of 254–275 nm—especially 267 nm—for high-efficacy viral inactivation in laboratory and controlled settings.

Report viral inactivation using $\log_{10}$-reduction values and include at least five data points per dose-response curve to ensure reliable modeling.

Distinguish terminology clearly: use “inactivation” for laboratory processes and “disinfection” for applied, real-world settings.

Confirm “complete inactivation” using multi-passage infectivity assays and nucleic acid degradation methods (e.g., qPCR), aligned with WHO standards.

Use 222 nm UV-C with caution, only in low-risk environments, and validate with proteomic and genomic assays due to limited safety and efficacy data.

3. Table of contents

Abstract

1. **Executive Summary**
   - 2.1 Methods
   - 2.2 Interpretation of Recommendations

2. **Introduction**
   - 3.1 Rationale
   - 3.2 Objectives
   - 3.3 PICO Framework

3. **Methods**
   - 4.1 Eligibility Criteria
   - 4.2 Information Sources
   - 4.3 Search Strategy
   - 4.4 Study Selection
   - 4.5 Data Collection and Items
   - 4.6 Risk of Bias Assessment
   - 4.7 Effect Measures
   - 4.8 Synthesis Methods
   - 4.9 Sensitivity Analysis
   - 4.10 Reporting Bias and Certainty

4. **Results**

5.1 Study Selection and Characteristics

5.2 Individual Study Outcomes

5.3 Meta-Analysis and Statistical Synthesis

5.4 Heterogeneity and Moderator Effects

5.5 Sensitivity and Bias Analysis

5.6 Certainty of Evidence

5. **Discussion**

6.1 Summary of Key Findings

6.2 Limitations of Evidence

6.3 Limitations of the Review Process

6.4 Implications for Practice, Policy, and Research

4. Introduction

The increasing application of ultraviolet-C (UV-C) irradiation in healthcare, biotechnology, and environmental safety highlights the need for standardized guidelines on viral inactivation. However, literature reveals significant variability in experimental conditions, terminological ambiguity (e.g., "inactivation" vs. "disinfection"), and inconsistent reporting of critical parameters such as UV dose, viral strain, and matrix composition. This guideline was developed to address these gaps by synthesizing evidence from 33 studies on strain-specific UV-C inactivation in liquid media. Through meta-analysis and thematic review, we aim to inform researchers and policy-makers on best practices, optimal UV-C wavelengths.

5. Scope and purpose

5.1. Target audience

This guideline is intended for virologists, biomedical researchers, clinical laboratory professionals, regulatory agencies, infection control specialists, and public health policy-makers involved in viral inactivation protocols and biosafety evaluations.

5.2. How to use these guidelines

Use these guidelines to design and evaluate UV-C viral inactivation protocols with rigor and reproducibility. Researchers should apply the strong recommendations for wavelength selection, dose standardization, and assay confirmation. Policy-makers can adopt the terminology and reporting framework to harmonize regulatory standards. The guidelines are structured to support both experimental and applied contexts and are compatible with WHO biosafety principles.

6. Methods

6.1. Group composition

The guideline development group included two primary reviewers with expertise in virology, biosafety, and systematic review methodology, along with a third adjudicator with expertise in statistical modeling and evidence grading. All members were trained in PRISMA, GRADE, and ROBINS-I V2 protocols. External consultation was conducted with domain experts in UV disinfection, microbiology, and public health regulation to ensure technical accuracy and translational applicability.

6.2. Group interaction and processes

An initial online training session was conducted to harmonize understanding of inclusion criteria and data extraction. Reviewers independently screened titles, abstracts, and full texts, using a structured MS Excel database and MS Forms to ensure traceability. Discrepancies were resolved through consensus discussion or by involving a third reviewer. All meta-analytical and risk-of-bias judgments followed predefined protocols, with synthesis validated using SPSS and Power BI. Regular consensus meetings and version-controlled documentation supported transparency throughout the process.

6.3. Declaration and management of competing interests

All authors declared no financial or personal competing interests that could influence the systematic review. Employment by RoLink Biotechnology Ltd. was transparently disclosed, and funding by the National Research, Development, and Innovation Office of Hungary had no role in study design, data collection, or interpretation.

6.4. Selection of questions and outcomes of interest

The research question was formulated using a structured PICO framework: *In RNA and DNA viruses (Population)*, *how*

does UV-C light exposure (100–280 nm) (Intervention), under varied operational conditions (Comparison), influence viral inactivation and disinfection outcomes (Outcome)? The outcomes of interest were selected based on biosafety relevance and measurable virological endpoints, including log_{10} viral titre reduction, complete inactivation status, and experimental UV dose (mJ/cm^2). Secondary outcomes included wavelength-specific effects, matrix interference (e.g., plasma vs. PBS), and assay type used ($TCID_{50}$, PFU, qPCR). Linguistic usage of “inactivation” vs. “disinfection” was also analyzed to support standardization across fields. Selection was guided by PRISMA, GRADE, and biosafety-level operational relevance.

6.5. Evidence review and development of clinical recommendations

The development of clinical and laboratory recommendations was informed by a systematic review and meta-analysis of 33 non-randomized intervention studies published between 2019 and 2024. The selection process adhered to PRISMA, GRADE, and MECIR guidelines, with risk of bias assessed via ROBINS-I V2. Data extraction was standardized using predefined variables (e.g., viral strain, UV wavelength, dose, assay type). Meta-regression, ANOVA, and sensitivity analyses (robust SE, bootstrap CI) were applied to identify predictors of effective inactivation. Recommendations were based on both quantitative effect estimates (e.g., LOG_DOSE $\beta = 3.382$) and qualitative thematic findings. Strong recommendations were derived from high-certainty evidence; conditional ones reflected moderate or indirect evidence.

6.6. Use of indirect evidence

Indirect evidence was used in cases where surrogate viruses (e.g., Phi6, MS2) were studied instead of high-risk human pathogens (e.g., SARS-CoV-2) due to biosafety constraints. Additionally, findings from UV-C inactivation in surrogate media (e.g., PBS, DMEM) were extrapolated to more complex matrices (e.g., plasma), acknowledging that matrix opacity and composition modulate inactivation efficacy. These extrapolations were clearly marked in the analysis and contributed to conditional recommendations only. Interpretations emphasized the importance of matrix validation and strain-specific assessments for clinical applicability.

6.7. Document review

Data extraction sheets, risk of bias assessments, and statistical outputs were reviewed independently by at least two reviewers. Each document underwent iterative revisions following feedback from internal team discussions and expert consultations. External reviewers with domain expertise in virology provided additional validation for methodological clarity and relevance. Final recommendations were reviewed for consistency with GRADE criteria, and discrepancies were resolved through structured and documented consensus meetings.

7. How to use these guidelines

These guidelines are designed to support the implementation of UV-C viral inactivation protocols across both laboratory and applied settings. Users should select appropriate UV wavelengths based on the desired outcome (e.g., experimental preservation vs. surface disinfection), and confirm viral reduction using validated infectivity assays. Strong recommendations apply to high-certainty evidence, such as the use of 267–275 nm light in transparent media, while conditional recommendations caution against unverified generalization to complex matrices or 222 nm use without further safety validation. The terminology (“inactivation” vs. “disinfection”) should be applied in line with the context and methodological rigor of the setting. Protocol developers, biosafety committees, and researchers should align procedures with the reporting framework proposed.

8. General issues necessary for correct interpretation and implementation of recommendations

8.1. Assumed Values and preferences

We assume that users value high biosafety standards, methodological transparency, and reproducibility in viral inactivation protocols. We presume a preference for precise experimental documentation (e.g., UV dose, matrix type, viral strain) and a possible commitment to using orthogonal assays for inactivation confirmation. Furthermore, it is assumed that end users prioritize dose-response clarity over convenience in disinfection protocols, and that they support the differentiation of experimental and applied terminology. These preferences reflect a global trend toward harmonization in infection control, laboratory safety, and translational virology research.

8.2. Recommendations for children

No direct recommendations can be made for pediatric applications due to the absence of child-specific clinical or experimental studies in the included literature. However, if UV-C disinfection is applied in environments frequented by children (e.g., schools, pediatric wards), only systems with proven safety profiles should be considered, and only under strict regulatory oversight. Further research is needed to assess tissue-specific risks and safety thresholds in pediatric populations.

8.3. Coexisting conditions

We assume usage in controlled environments without direct human exposure; therefore, no patient-specific clinical comorbidities were analyzed. If applied in settings involving immunocompromised or critically ill individuals (e.g., transfusion, respiratory therapy), extra care must be taken to validate both the sterility assurance level and absence of protein denaturation in medical products. Indirect evidence suggests UV-C can preserve or degrade proteins depending on wavelength and fluence, which may be relevant for coexisting immune or hematologic conditions.

9. Key questions

1. **What is the efficacy of UV-C light (100–280 nm) in inactivating RNA and DNA viruses in liquid media under controlled conditions?**

2. **Which UV-C wavelengths provide the highest $\log_{10}$ viral load reduction, and how do these effects vary by viral strain and matrix composition?**

3. **What experimental variables (e.g., dose, wavelength, exposure duration, matrix) significantly influence viral inactivation outcomes?**

4. **How should the terms "inactivation" and "disinfection" be accurately applied in laboratory versus real-world applications?**

5. **What constitutes “complete viral inactivation,” and how can this be methodologically confirmed across detection systems (e.g., $TCID_{50}$, plaque assay, qPCR)?**

6. **Can surrogate viruses reliably represent pathogenic strains in UV-C efficacy studies, and under what constraints?**

10. Recommendations

10.1. Table summarizing recommendations

**Recommendation**	**Strength**	**Rationale**
Standardize UV-C experimental reporting (dose, matrix, virus strain, etc.)	Strong	Critical for reproducibility and meta-analysis reliability
Use "inactivation" for lab virus studies and "disinfection" for field applications	Strong	Reduces semantic confusion and improves protocol precision
Adopt "absence of infectivity after three blind passages" as the definition for "complete inactivation"	Strong	Ensures rigorous biosafety confirmation
Evaluate matrix transparency and stirring effects during UV-C experiments	Conditional	Influences UV-C effectiveness, though context-dependent
Carefully assess the safety profile of 222 nm UV-C before large-scale human exposure	Conditional	Higher biocidal activity but possible underreported human tissue risks

10.2. <Question 1>

1. **What is the efficacy of UV-C light (100–280 nm) in inactivating RNA and DNA viruses in liquid media under controlled conditions?**

UV-C irradiation is highly effective for inactivating both RNA and DNA viruses in liquid solutions, with a clear, dose-dependent relationship. The meta-regression found that the logarithmic UV dose (LOG_DOSE) significantly predicted log_{10} viral reduction ($\beta = 3.382$, 95% CI [2.946, 3.817], $p < 0.001$), and this held across sensitivity models, confirming high efficacy when exposure is properly controlled.

2. Which UV-C wavelengths provide the highest log_{10} viral load reduction, and how do these effects vary by viral strain and matrix composition?

267 nm and 275 nm yielded the highest efficacy in log_{10} reduction ($\beta = 6.418$ and 3.776, respectively), outperforming the standard 254 nm. Viral strain affected outcomes significantly—e.g., P.1 and Omicron BA.2 showed lower susceptibility than USA-WA1/2020. Matrix composition, particularly opacity and turbidity (e.g., plasma vs. PBS), significantly impacted effectiveness due to light absorption differences.

3. What experimental variables (e.g., dose, wavelength, exposure duration, matrix) significantly influence viral inactivation outcomes?

Key influencing variables include UV wavelength, dose (especially LOG_DOSE), virus strain, matrix opacity, stirring, and the optical clarity of the suspension medium. Inactivation is significantly enhanced in transparent solutions and diminished in turbid media like red blood cell suspensions. Distance, exposure time, and assay sensitivity also impact outcomes and were heterogeneously reported across studies.

4. How should the terms "inactivation" and "disinfection" be accurately applied in laboratory versus real-world applications?

“Inactivation” should be used for laboratory experiments that maintain viral structures for downstream applications, while “disinfection” refers to applied settings like hospitals or water treatment where the goal is eliminating infectivity. Your analysis found inconsistent usage across studies and proposed this clear distinction to align language with context and biosafety relevance.

5. What constitutes “complete viral inactivation,” and how can this be methodologically confirmed across detection systems (e.g., $TCID_{50}$, plaque assay, qPCR)?

Complete inactivation is defined as the absence of detectable infectivity via highly sensitive, validated assays (e.g., multi-passage $TCID_{50}$ or plaque assay), and corroborated by qPCR evidence of genome degradation. This must fall below the detection threshold across all methods used. This definition addresses the variability seen in prior literature and aligns with WHO standards.

6. Can surrogate viruses reliably represent pathogenic strains in UV-C efficacy studies, and under what constraints?

Surrogates like bacteriophage MS2 or Phi6 are commonly used, but their UV-C susceptibility varies even within strains, limiting generalizability. We found that surrogate-based conclusions must be cautiously interpreted and always validated against the target virus, as structural and genomic differences influence inactivation kinetics significantly.

11. Plans for updating these guidelines

These guidelines should be reviewed and updated every 3–5 years, or sooner if significant new evidence emerges on UV-C inactivation kinetics, new UV technologies (e.g., novel 222 nm systems), or if biosafety regulations change substantially. Updates should follow PRISMA and GRADE methodologies to maintain consistency and reliability.

12. Updating or adapting recommendations locally

These recommendations should be revisited periodically in response to new scientific evidence, technological advances in UV-C delivery systems (e.g., pulsed xenon, filtered far-UVC), or emerging pathogen strains with differing UV susceptibilities. Local adaptation may be required based on region-specific biosafety standards, available disinfection infrastructure, and matrix types (e.g., water, blood products, aerosols). Institutions are encouraged to validate UV-C protocols in situ, ensure assay compatibility, and align terminology with local regulatory expectations. Integration with WHO biosafety levels, MIQE, or ISO standards is strongly advised. Updates should prioritize risk assessment data and reproducibility of dose-response findings under localized environmental conditions.

13. Conclusions

This guideline emphasizes the urgent need for standardization in UV-C viral inactivation research, particularly in experimental reporting, terminology usage, and biosafety definitions. Adoption of these recommendations will enhance scientific reproducibility, ensure safer application of UV-C technologies, and facilitate more effective policymaking and regulatory approval processes. Transparent, reproducible UV-C protocols will significantly benefit research, healthcare, and public health sectors in their efforts against viral threats.

14. References

15. Appendices

15.1. Appendix 1

Upon request we can make the following available:

- Full PICO search strings and search strategies across PubMed, Embase, Scopus, Web of Science, and Ovid databases (search date: April 10, 2024).
- Data extraction sheet template (Excel-based, including virus strain, UV-C wavelength, dose, viral reduction data, and experimental setup details).
- Risk of bias assessment details using ROBINS-I V2 with domain-specific judgments per study.
- Supplementary material on semantic analysis results ("inactivation" vs. "disinfection" word frequencies and contexts).
- Additional graphical figures generated via Power BI illustrating viral inactivation patterns, dose-response models, and experimental parameter distributions.
